# From *In Situ* to satellite observations of pelagic *Sargassum* distribution and aggregation in the Tropical North Atlantic Ocean

**DOI:** 10.1371/journal.pone.0222584

**Published:** 2019-09-17

**Authors:** Anouck Ody, Thierry Thibaut, Léo Berline, Thomas Changeux, Jean-Michel André, Cristèle Chevalier, Aurélie Blanfuné, Jean Blanchot, Sandrine Ruitton, Valérie Stiger-Pouvreau, Solène Connan, Jacques Grelet, Didier Aurelle, Mathilde Guéné, Hubert Bataille, Céline Bachelier, Dorian Guillemain, Natascha Schmidt, Vincent Fauvelle, Sophie Guasco, Frédéric Ménard

**Affiliations:** 1 Aix-Marseille Univ., Université de Toulon, CNRS, IRD, MIO UM 110, Marseille, France; 2 Université de Bretagne Occidentale (UBO), Institut Universitaire Européen de la Mer (IUEM), LEMAR UMR 6539, Technopôle Brest-Iroise, Plouzané, France; 3 IRD DR-OUEST, US191 IMAGO, Technopole de Brest-Iroise—Site de la Pointe du Diable, Plouzané, France; 4 Université des Antilles, UMR BOREA, Campus de Fouillole, BP 592, Pointe-à-Pitre, Guadeloupe, France; 5 IRD, IRD-Images, Marseille, France; 6 Aix Marseille Univ, CNRS, IRD, IRSTEA, OSU PYTHEAS, Marseille, France; Universite de Nantes, FRANCE

## Abstract

The present study reports on observations carried out in the Tropical North Atlantic in summer and autumn 2017, documenting *Sargassum* aggregations using both ship-deck observations and satellite sensor observations at three resolutions (MSI-10 m, OLCI-300 m, VIIRS-750 m and MODIS-1 km). Both datasets reported that in summer, S*argassum* aggregations were mainly observed off Brazil and near the Caribbean Islands, while they accumulated near the African coast in autumn. Based on *in situ* observations, we propose a five-class typology allowing standardisation of the description of *in situ Sargassum* raft shapes and sizes. The most commonly observed *Sargassum* raft type was windrows, but large rafts composed of a quasi-circular patch hundreds of meters wide were also observed. Satellite imagery showed that these rafts formed larger *Sargassum* aggregations over a wide range of scales, with smaller aggregations (of tens of m^2^ area) nested within larger ones (of hundreds of km^2^). Match-ups between different satellite sensors and *in situ* observations were limited for this dataset, mainly because of high cloud cover during the periods of observation. Nevertheless, comparisons between the two datasets showed that satellite sensors successfully detected *Sargassum* abundance and aggregation patterns consistent with *in situ* observations. MODIS and VIIRS sensors were better suited to describing the *Sargassum* aggregation distribution and dynamics at Atlantic scale, while the new sensors, OLCI and MSI, proved their ability to detect *Sargassum* aggregations and to describe their (sub-) mesoscale nested structure. The high variability in raft shape, size, thickness, depth and biomass density observed *in situ* means that caution is called for when using satellite maps of *Sargassum* distribution and biomass estimation. Improvements would require additional *in situ* and airborne observations or very high-resolution satellite imagery.

## Introduction

Harmful macroalgal blooms have become a global concern, causing ecological, economic and health problems [1–4]. Among the most widely proliferating seaweeds, some species of the genus *Sargassum* C. Agardh (Phaeophyceae, Fucales) are causing serious threats to coastal ecosystems [5–8]. On the European Atlantic coast and in the Mediterranean Sea, *Sargassum muticum* (Yendo) Fensholt can have an ecological and economic impact (e.g. [9–15]). Benthic species can form floating and drifting rafts on the surface of the oceans, such as *Sargassum horneri* (Turner) C. Agardh, spreading on American and Asian coasts (e.g. [16–20]), and *Sargassum pacificum* Bory in the South Pacific [7,8,21]. These benthic species are however unable to travel very long distances due to the degradation of their thalli. In the North Atlantic, pelagic *Sargassum* thalli float and grow at the sea surface during their entire lifetime. They can aggregate, forming *Sargassum* rafts which can travel long distances under the action of winds, waves and currents (e.g. [22,23]). Pelagic *Sargassum* is common in the Sargasso Sea [24,25] as well as in the Gulf of Mexico where it recurrently strands in large quantities on the coasts [4,26].

Since 2011, massive new strandings of pelagic *Sargassum* have been reported on the coasts of the Caribbean, northern Brazil, French Guiana and West Africa, causing widespread economic and ecological damage (e.g. [2]). Three morphotypes of pelagic *Sargassum* have been reported from the new areas of the Caribbean and Tropical Atlantic [27,28]. These massive strandings on the coasts are associated with the extensive occurrence of *Sargassum* aggregations in the Tropical North Atlantic, as observed through satellite imagery (e.g. [29,30]). Satellite images and direct observations of beaching events show that the phenomenon does not occur every year, making it difficult to forecast these proliferations and to set up suitable management procedures (e.g. [30–32]). A better understanding of the distribution, dynamics, structure and biomass of these pelagic *Sargassum* aggregations based on *in situ* observations and satellite imagery is of primary importance for the assessment of their ecological significance and for improving the forecasting and management of these strandings.

*In situ* ship-deck observations of *Sargassum* rafts have been conducted for many years, together with attempts to quantify *Sargassum* biomass using surface net tows (e.g. [23–25,33–35]). These observations were mostly restricted to the Sargasso Sea and the Gulf of Mexico (see [24] for a review), and for limited periods the Atlantic Ocean. Several categories of *Sargassum* rafts were reported [22,23,25,36,37], but no simple typology has been proposed to fully describe the variety of rafts.

There has been constant progress in the development of remote sensing for floating marine vegetation such as *Sargassum* species, in order to provide accurate information on their distribution and to quantify their coverage area and biomass (e.g. [21,29,30,38–48]). The occurrence of large pelagic *Sargassum* aggregations in the Tropical North Atlantic was first detected from space in 2011 using the MEdium Resolution Imaging Spectrometer (MERIS, on board the ENVISAT satellite), with a spatial resolution of 300 m [29]. After May 2012, *Sargassum* detection in the Tropical North Atlantic was performed using the MOderate Resolution Imaging Spectroradiometers (MODIS, on board the AQUA and TERRA satellites) and the Visible Infrared Imaging Radiometer Suite (VIIRS, on board the SNPP NASA satellite), with a coarser spatial resolution of 1 km and 750 m, respectively (e.g. [30,47]), precluding the fine-scale description of *Sargassum* aggregations. High-resolution sensors on board Landsat platforms with a 30 m resolution were also widely used to map the distribution of *Sargassum* [43,49,50], but images were restricted to coastal areas only.

In this study, we have exploited new data from the recently launched ESA higher resolution satellite sensors, namely the Ocean and Land Colour Instrument (OLCI, 300 m) on board Sentinel-3, and the MultiSpectral Instrument (MSI, 60-20-10 m) on board Sentinel-2, coupled with new *in situ* observations carried out during two oceanographic cruises dedicated to the study of the 2017 *Sargassum* proliferation. First, we propose a simple standardized typology to describe *in situ Sargassum* rafts, such as those established for other invasive seaweeds (e.g. [51,52]). Such a typology, if it proves sufficiently reliable to be adopted, would allow simple and direct description of the raft shapes and sizes. In our work, this typology is then used to describe the various *Sargassum* rafts encountered during the two cruises. Secondly, we compare and interpret remote-sensing observations of *Sargassum* aggregations, provided at various nested spatial scales by the MODIS, VIIRS, OLCI and MSI satellite sensors, in the light of these *in situ* observations. These two datasets provided a basis for discussion of (i) the distribution and structure of *Sargassum* aggregations at various scales in relation with their environment, and (ii) the ability of the four sensors to provide accurate information on the distribution and the structure of S*argassum* aggregations at each spatial scale. Finally, implications for *Sargassum* quantification and biomass estimation from space are addressed.

## Material and methods

### Field cruises

**Fig 1:**
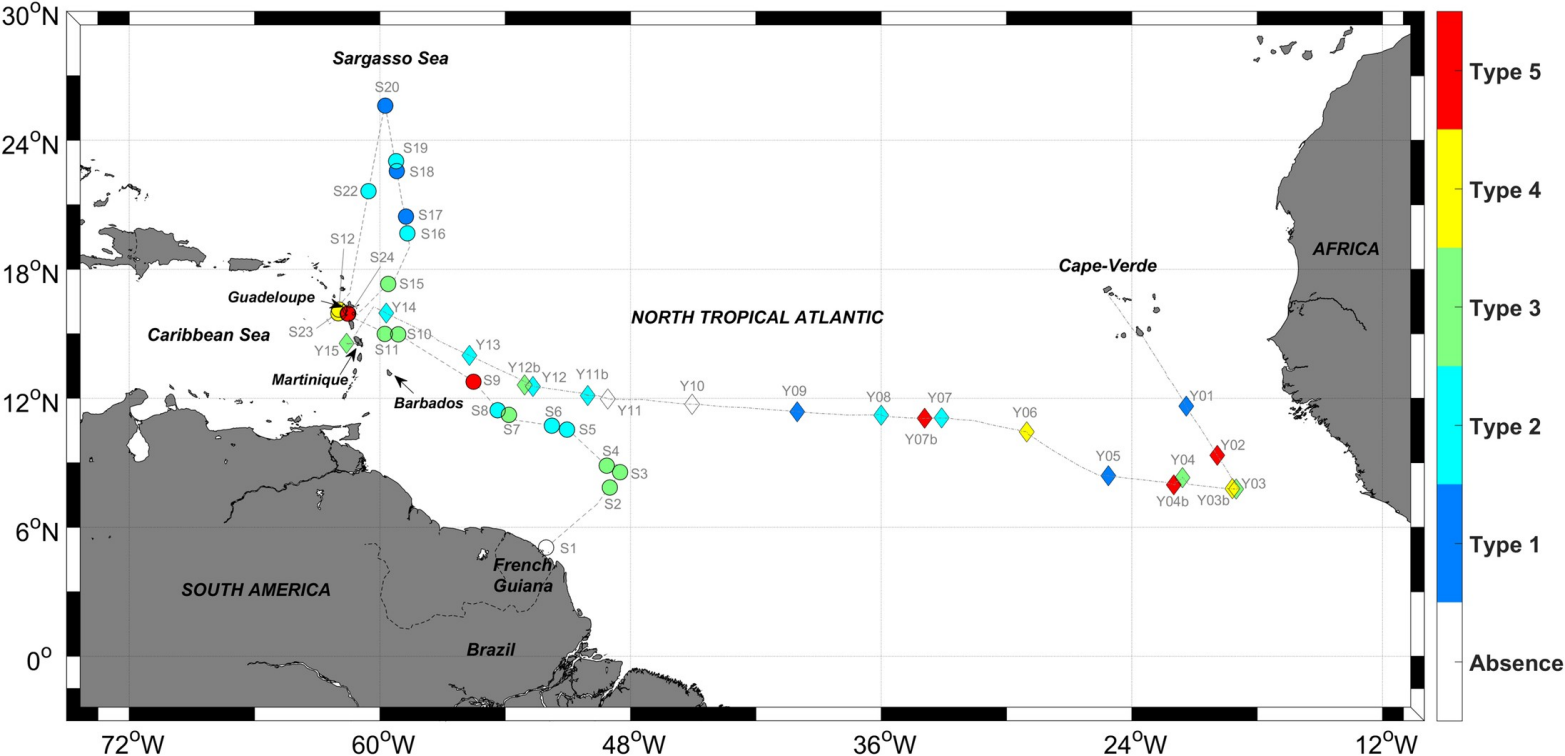
*West Atlantic* and *Transatlantic* cruise tracks and stations. *West Atlantic* cruise stations are represented with circles and *Transatlantic* cruise stations with diamonds. Colors correspond to the *Sargassum* raft type (highest, described in Fig 2) observed at each station.

Two cruises were carried out in the Tropical North Atlantic in 2017 to investigate the *Sargassum* distribution in the field, to collect samples for genetic analyses, to obtain an overview of the population structure of *Sargassum*, to assess the contamination by trace metals in *Sargassum* tissues and to study the composition of *Sargassum*-associated faunal assemblages and trophic web through stable isotope measurements (Fig 1). The *West Atlantic—Sargassum Expedition* (http://dx.doi.org/10.17600/17004300) took place on board the N/O ANTEA from June 19^th^ to July 13^th^ 2017, and explored the new high *Sargassum* abundance region situated between the north-east coast of Brazil and the Caribbean arc, as well as the historical Sargasso Sea (25°N). The *Transatlantic—Sargassum Expedition* (http://dx.doi.org/10.17600/17016900) took place on board the M/V YERSIN from October 6^th^ to 24^th^ 2017 from the Cape Verde Islands, crossing the Atlantic between 8 and 12°N, to the island of Martinique. The aim of the latter cruise was to sample the western part of the Tropical North Atlantic, where *Sargassum* commonly accumulates from September to November [29], and to cover a transatlantic transect. Both cruises were routed using satellite images and Lagrangian simulations provided by our onshore team at the laboratory (MIO) in order to guide the ship to areas of high *Sargassum* abundance. The locations of the sampling stations were first identified based on satellite-derived maps of the *Sargassum* distribution, then chosen on the basis of the *in situ* observations of *Sargassum*, as well as the weather, the sea conditions and the distance from the previous station. If no *Sargassum* were observed, at least one station per day was performed for water sampling. In addition, on the *Transatlantic* cruise, one additional station was scheduled every day for water contamination sampling [53]. It should be noted that no station could be done outside international and French territorial waters.

### Terminology used to describe *Sargassum* observations

We use hereafter the word 'raft' as a generic term to describe an *in situ* continuous aggregation of *Sargassum* thalli. Round-shaped *Sargassum* thalli aggregations are referred to as ‘patches’. Line-shaped *Sargassum* thalli aggregations are referred to as 'windrows'. A raft can be made of an assemblage of windrows and patches. We use the term ‘raft shape’to define this assemblage.

As most satellite sensors do not discriminate between individual rafts, the term '*Sargassum* aggregation' is used instead of ‘*Sargassum* raft’ for the interpretation of *Sargassum* detections on satellite images. A *Sargassum* aggregation can then represent either individual *Sargassum* rafts (for high-resolution sensors) or aggregations of several rafts into a larger *Sargassum* structure. We use the term ‘aggregation structure’ to define the spatial organisation of these aggregations. When *Sargassum* aggregations detected from space have an elongated structure, we use the term '*Sargassum* filament'.

### Typology of *Sargassum* rafts from *in situ* observations

*Sargassum* observations were made during the two cruises in daylight from the ship deck, generally from ~ 6 m above water level, by different observers.

Pelagic *Sargassum* algae are made of more or less branched axes with blades and air bladders, but do not show a defined structure (holdfast, main axis, secondary branches) as reported for benthic taxa. The observed *Sargassum* thalli were of various sizes, from a tiny axis with a few fronds to an axis more than 1 m long. *Sargassum* thalli were sometimes found isolated and scattered, but we mostly observed aggregated *Sargassum* rafts of variable shape and size.

**Fig 2:**
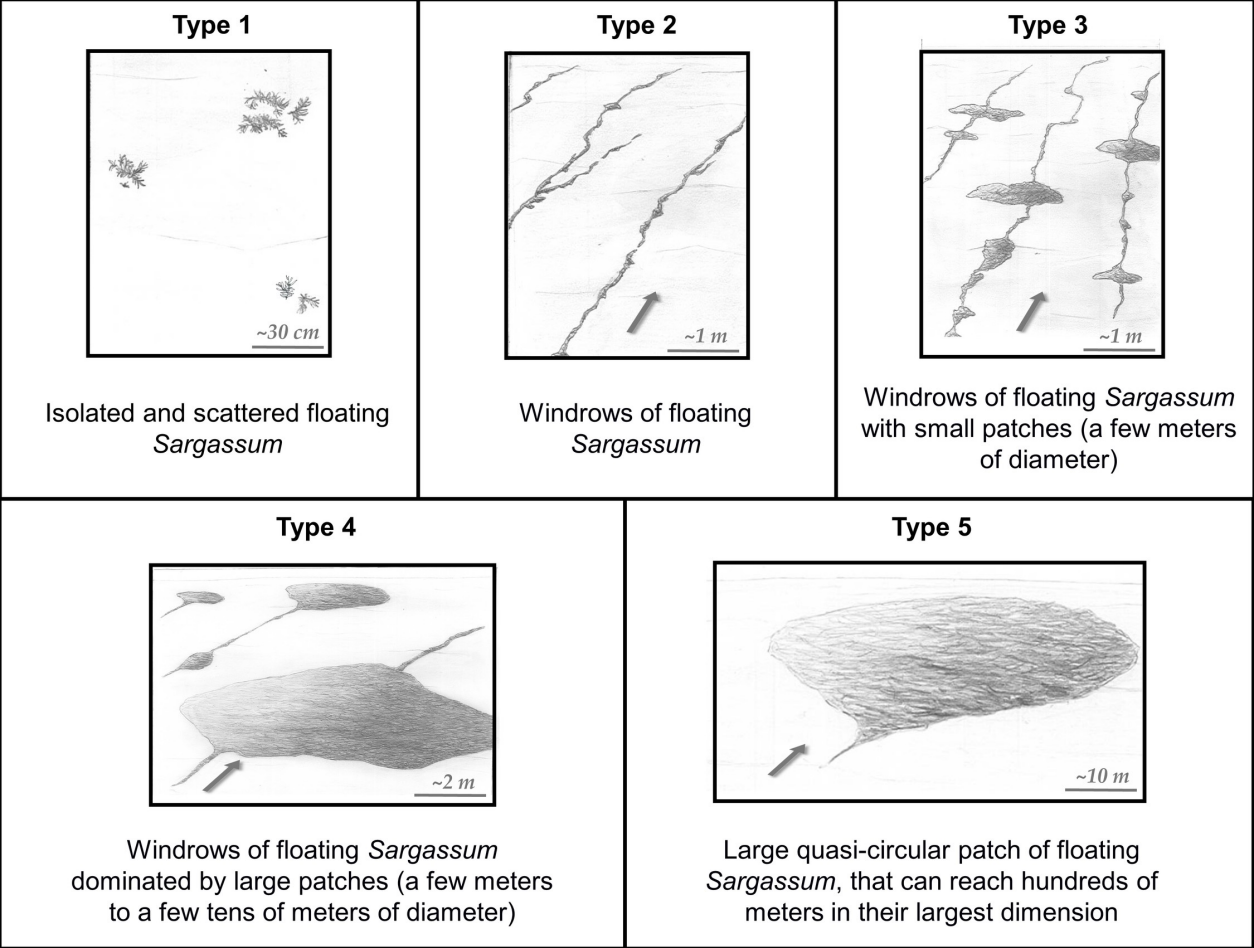
Five-class typology of *Sargassum* rafts illustrated with schematic drawings. Drawings are based on observations from the ship deck ~ 6 m above water level. Wind direction is indicated by an arrow. Original drawings by Emma Rozis.

Based on our observations and the literature concerning the Sargasso Sea (e.g., [22,23,25]), we developed a five-class typology to provide a fast, simple and standardized method to report valuable scientific information regarding the characteristics of *in situ Sargassum* rafts. Types are numbered from 1 to 5. Type 1 corresponds to isolated floating *Sargassum*, while types 2 to 5 correspond to *Sargassum* rafts, according to their shape and size. This typology is described in Fig 2.

We assigned a *Sargassum* raft type to stations from the two cruises. As the typology was defined at the end of the *West Atlantic* cruise, the *Sargassum* raft type for each station was assigned later using observations reported in the cruise logbook, including pictures (information was missing for three stations (S13, S14, S21) not reported on Fig 1). For the *Transatlantic* cruise, the type was directly assigned during observations. If more than one of the *Sargassum* raft types were observed next to each other at a given station, we only reported the type with the highest number (1 to 5) in our scale, which corresponds to the largest observed rafts.

### Satellite imagery

We detected *Sargassum* aggregations using satellite imagery at different spatial resolutions during the two cruises. Table 1 displays the satellite sensor specifications as well as the algae indexes used to detect *Sargassum*.

**Table 1:** Characteristics of the satellite sensors and algae indexes used in this study.

	MODIS (A & T)	VIIRS	OLCI (A)	MSI (A & B)
Spatial Resolution	1 km	750 m	300 m	10 m
Temporal Resolution	1 day	1 day	4 days	5 days
Cross Track	2 330 km	3 040 km	1 270 km	290 km (coastal areas)
Algae Index	AFAI^1^	AFAI^1^	MCI^2^	MSI-MFAI^3^
Radiometric data*	Rayleigh-corrected reflectance**	Rayleigh-corrected reflectance**	Top-of-atmosphere radiance***	Top-of-atmosphere reflectance****
Wavebands	λ_1_ = 667 nm	λ_1_ = 671 nm	λ_1_ = 681 nm	λ_1_ = 665 nm
	λ_2_ = 748 nm	λ_2_ = 745 nm	λ_2_ = 709 nm	λ _2_ = 833 nm
	λ_3_ = 869 nm	λ_3_ = 862 nm	λ_3_ = 754 nm	λ_3_ = 940 nm

^1^ [30]

^2^ [54]

^3^Adapted from [55]

*Because of the high NIR reflectance, the traditional Ocean Colour atmospheric correction scheme cannot be applied, and spectral indexes had to be derived from top-of-atmosphere radiances (Lt) or reflectance (Rt) or from Rayleigh-corrected reflectance (Rrc).

**Downloaded from the Ocean colour website (https://oceancolor.gsfc.nasa.gov/) and processed using Seadas Software (L2gen function)

*** Downloaded from the Copernicus S-3 data Hub (https://scihub.copernicus.eu/s3/#/home)

**** Downloaded from the Copernicus S-2 data Hub (https://scihub.copernicus.eu/dhus/#/home).

Algae indexes are based on the red-edge effect of floating vegetation, i.e. an increase in the *Sargassum* radiometric signal in the near-infrared (NIR) wavelength range (650–1200 nm) (e.g. [43]). All algae indexes used in this study are defined as the difference between the reflectance (or radiance; *r*) in the central waveband (*λ*_*2*_), corresponding to the maximum of the red-edge effect and a linear baseline drawn between surrounding bands (*λ*_*1*_ and *λ*_*3*_ respectively) [30,54,55], and can be written as:

$$Algae\;Index=r(\lambda_{2})-\left[r(\lambda_{1})+\left(r(\lambda_{3})-r(\lambda_{1})\right)\times \frac{\lambda_{2}-\lambda_{1}}{\lambda_{3}-\lambda_{1}}\right]$$

The satellite sensors MODIS-A, MODIS-T and VIIRS, on board the AQUA, TERRA and SNPP NASA polar orbital platforms, respectively, provided images with a cross track of 2 330 km and a spatial resolution of 1 km, 500 m or 250 m (depending on the spectral bands) for MODIS, and a swath width of 3 040 km and a spatial resolution of 750 m or 350 m (depending on the spectral bands) for VIIRS. MODIS and VIIRS ensure quasi-global coverage of the Earth each day. Two algae indexes, the Floating Algae Index (FAI) and the Alternative Floating Algae Index (AFAI), are currently used to detect *Sargassum* using MODIS data. The FAI uses the MODIS high-resolution spectral bands (250 m; [55]) but is very sensitive to clouds, making it difficult to differentiate *Sargassum* from clouds and other artifacts [30]. Therefore, we used the AFAI index based on the MODIS 1 km OC spectral bands instead [30]. This index is computed from the Rayleigh-corrected reflectance (Rrc) using the l2gen function of the Seadas Software (https://seadas.gsfc.nasa.gov/). We also used the AFAI index to detect *Sargassum* from VIIRS data using its 750 nm spectral band.

The OLCI sensor on board the Sentinel-3A ESA satellite provides images with a cross track of 1 270 km at a spatial resolution of 300 m, and with a revisiting time of ~4 days at low latitude (< 30°). We used the Maximum Chlorophyll Index (MCI) to detect *Sargassum* with OLCI data [54]. The MCI index is directly computed from the provided top-of-atmosphere radiance without any pre-processing.

The MSI sensor on board the Sentinel-2 (A & B) ESA platforms provides images at a very high resolution reaching 10 m, but with a revisiting time of 5 days. These images, with a cross track of 290 km, were acquired in coastal areas (20 km) only, and thus provided very low ocean coverage. The high resolution of this sensor allowed us to observe pixels with quasi-total *Sargassum* coverage, leading to a very strong red-edge signal, with a maximum reflectance shifted to 800 nm (compared to a maximum around 700 nm for pixel observing *Sargassum* mixed with water; [38,43]). The FAI index, based on the 833 nm MSI spectral band, associated with the 665 nm and 1610 nm bands, thus seems to be the best suited to detect *Sargassum* with MSI. However, this index is highly sensitive to the sensor parallax effect caused by a difference in the viewing angle of sensor detectors (S1 Fig, [56]). This effect leads to wide stripe patterns in surface reflectance across images, precluding the attribution of a common threshold for the entire image (S1 Fig). Using the 940 nm band instead of the 1610 nm band considerably diminished this effect. The 940 nm spectral band is located on the atmospheric water vapour absorption band, and is thus not intended for use in surface observation. However, atmospheric water vapour shows large-scale patterns that cannot be confused with *Sargassum* aggregations. Moreover, comparison of images produced using the FAI and images using the 940 nm band shows that the use of the latter enables the detection of all *Sargassum* aggregations with greater accuracy and visibility than with the FAI (S1 Fig). This index, referred to as MSI-MFAI for MSI-Modified Floating Algae Index hereafter, was thus pragmatically used to detect *Sargassum* aggregations with the MSI sensor. This index benefits from the 10 m resolution of the 833 and 665 nm bands allowing the detection of *Sargassum* with the highest resolution provided by the MSI sensor. The 940 nm MSI spectral band has a native spatial resolution of 60 m, and is thus up-sampled to a 10 m resolution by bilinear interpolation (using the SNAP software S2 Resampling tool) before computing the index. This index is directly computed from the provided top-of-atmosphere reflectance.

On all satellite images, we masked clouds. On MODIS and VIIRS images, we applied the SeaDAS cloud masking algorithm, using the Rrc at 2130 nm (MODIS) or 2250 nm (VIIRS), with a threshold set at 0.03, enabling us to mask clouds without masking *Sargassum* detection. With OLCI, we used the cloud flag provided with the radiometric data. With MSI, clouds were masked using the criteria R_t_(665) > 0.15 adapted from [30] (where R_t_ is the top-of-atmosphere reflectance). To deal with cloud edge and/or cloud shadows, the cloud mask edges were dilated (1 pixel for MODIS and VIIRS, 2 pixels for OLCI and 3 pixels for MSI).

Algae index values are correlated with the *Sargassum* coverage within the pixel. They are nevertheless also sensitive to atmospheric effects (not corrected) and illumination conditions. For all satellite images, the index range was thus adapted to highlight the *Sargassum* signal (signal increases from green to yellow in the figures) with respect to the background low signal corresponding to pure water (mapped in blue). Clouds were mapped in black.

To extend the satellite sensor coverage over the study area, or to stack together multiple observations from different days, we computed composites using multiple MODIS and/or VIIRS images. Each image was re-projected on a common regular grid with a resolution of 0.01° x 0.01° (corresponding to about 1 km x 1 km). For pixels where several AFAI values were available, only the maximum value was kept.

The MODIS, VIIRS, OLCI and MSI images matching each station are reported in Tables 2 and 3 and in S2 and S3 Figs. For MODIS and VIIRS, we looked for data available on the day of the station. As OLCI has a lower temporal resolution, we looked for data from +/- 1 day. For MSI we looked for data from +/- 3 days.

**Table 2:** *In situ* dataset for the West Atlantic cruise, including *Sargassum* raft characterization, station metadata and availability of satellite imagery. Satellite availability is reported as A = Available; NA = Not available; BD = Bad data (cloud, glint, straylight, etc.); for OLCI and MSI, first symbol refers to availability on the day of the station and symbol in brackets to availability at +/– 1 day (OLCI) and +/– 3 days (MSI).

Station	date (dd/mm/yyyy)	Hour UTC	Latitude (°N)	Longitude (°W)	Wind Speed (m s^-1^)	Wind Direction (°N)	Sea State (Douglas Scale)	*Sargassum* raft Type (Highest)	Raft disaggregation state (vertical /horizontal)	MODIS-A	MODIS-T	VIIRS	OLCI	MSI
S1	21/06/2017	15:41	05°03.390’	52°02.580’	4.1	263.8	Slight	absence	no	A	BD	A	A	NA
S2	22/06/2017	20:18	07°50.950’	48°59.550’	6.6	245.2	Moderate	3	no	BD	BD	BD	NA (NA)	NA
S3	23/06/2017	10:35	08°33.710’	48°29.940’	5.5	254.0	Slight	3	no	BD	BD	BD	NA (BD)	NA
S4	23/06/2017	20:04	08°51.950’	49°08.380’	6.1	251.8	Slight	3	no	BD	A	BD	NA (A)	NA
S5	24/06/2017	12:24	10°32.630’	51°02.530’	8.4	257.8	Moderate	2	no	BD	BD	BD	A	NA
S6	24/06/2017	19:22	10°43.540’	51°46.030’	9.0	259.5	Moderate	2	no	BD	BD	BD	A	NA
S7	25/06/2017	12:08	11°13.690’	53°49.410’	8.8	246.5	Moderate	3	no	NA	BD	BD	NA (A)	NA
S8	25/06/2017	19:59	11°24.579’	54°14.634’	9.1	245.8	Moderate	2	no	BD	BD	BD	NA (BD)	NA
S9	26/06/2017	12:50	12°46.160’	55°31.040’	9.2	241.0	Moderate	5	no	BD	BD	BD	NA (BD)	NA
S10	27/06/2017	19:06	14°58.440’	59°07.230’	8.7	246.7	Moderate	3	no	BD	A	BD	A	NA
S11	28/06/2017	11:57	14°59.620’	59°45.870’	8.7	265.9	Moderate	3	no	BD	BD	BD	NA (A)	BD
S12	29/06/2017	13:58	15°57.270’	61°59.300’	7.8	276.9	Slight	4	no	BD	BD	BD	BD	NA
S15	03/07/2017	12:39	17°19.134’	59°36.044’	8.6	255.7	Moderate	3	no	BD	BD	A	NA (A)	NA
S16	04/07/2017	12:02	19°40.327’	58°41.059’	8.8	267.4	Moderate	2	vertical	BD	BD	BD	A	NA
S17	04/07/2017	20:39	20°27.113’	58°45.029’	8.6	270.0	Moderate	1	vertical	BD	BD	BD	BD	NA
S18	05/07/2017	11:32	22°34.038’	59°11.416’	7.2	269.6	Slight	1	vertical	BD	BD	BD	NA (A)	NA
S19	05/07/2017	18:35	23°01.740’	59°13.367’	7.0	273.6	Slight	2	no	BD	A	A	NA (A)	NA
S20	06/07/2017	11:40	25°36.575’	59°44.544’	5.1	273.3	Slight	1	no	A	A	A	NA (BD)	NA
S22	07/07/2017	18:02	21°38.080’	60°32.705’	8.0	267.1	Moderate	2	vertical	NA	A	A	BD	NA
S23	09/07/2017	14:44	16°07.366’	61°57.353’	3.6	314.1	Smooth	4	no	BD	BD	BD	NA (A)	NA
S24a	09/07/2017	16:30	15°56.023’	61°30.721’	4.1	288.1	Slight	5	no	BD	BD	BD	NA (A)	NA (A)
S24b	10/07/2017	12:54	15°56.087’	61°32.602’	5.5	260.2	Smooth	4	no	BD	NA	BD	NA (A)	NA

**Table 3:** *In situ* dataset for the *Transatlantic* cruise, including *Sargassum* raft characterization, station metadata and availability of satellite imagery. Satellite availability is reported as A = Available; NA = Not available; BD = Bad data (cloud, glint, stray light, etc.); for OLCI and MSI, first symbol refers to availability on the day of the station and symbol in brackets to availability at +/- 1 day (OLCI) and +/- 3 days (MSI).

Station	date (dd/mm/yyyy)	Hour (UTC)	Latitude (°N)	Longitude (°W)	Wind Speed (m s^-1^)	Wind Direction (°N)	Sea State (Douglas Scale)	*Sargassum* raft Type (Highest)	Raft disaggregation state (vertical /horizontal)	MODIS-A	MODIS-T	VIIRS	OLCI	MSI
Y01	07/10/2017	15:00	11°37.880’	21°23.610’	3.7	169.7	Slight	1	no	BD	BD	BD	BD	NA
Y02	08/10/2017	07:30	9°20.520’	19°55.060’	3.2	339.6	Slight	5	no	BD	BD	BD	BD	NA
Y03	09/10/2017	10:00	7°46.315’	18°59.865’	3.2	69.5	Slight	3	horizontal	BD	BD	BD	NA (A)	NA
Y03b	09/10/2017	15:00	7°47.464’	19°10.818’	3.2	64.8	Slight	4	horizontal	BD	BD	A	NA (A)	NA
Y04	10/10/2017	12:00	8°18.146’	21°34.633’	3.1	144.3	Slight	3	no	NA	NA	BD	BD	NA
Y04b	10/10/2017	18:00	7°58.917’	21°59.113’	3.1	140.8	Slight	5	no	NA	NA	BD	BD	NA
Y05	11/10/2017	10:00	8°23.260’	25°07.000’	4.2	221.6	Slight	1	no	BD	BD	BD	NA (A)	NA
Y06	12/10/2017	12:00	10°25.792’	29°02.017’	6.9	248.1	Slight	4	no	BD	A	BD	NA (BD)	NA
Y07	13/10/2017	10:00	11°04.908’	33°06.668’	7.3	266.6	Slight	2	no	BD	A	BD	BD	NA
Y07b	13/10/2017	19:30	11°04.036’	33°54.808’	7.4	266.1	Slight	5	no	BD	BD	A	A	NA
Y08	14/10/2017	11:30	11°13.086’	35°59.970’	7.2	255.7	Moderate	2	vertical	BD	BD	BD	NA (BD)	NA
Y09	15/10/2017	11:00	11°22.701’	40°01.604’	7.1	252.1	Slight	1	no	BD	BD	BD	A	NA
Y10	16/10/2017	12:00	11°43.499’	45°03.126’	8.8	256.5	Moderate	absence	no	BD	BD	BD	NA (BD)	NA
Y11	17/10/2017	12:00	11°57.953’	49°05.291’	9.4	256.5	rough	absence	no	NA	BD	BD	NA (A)	NA
Y11b	17/10/2017	17:45	12°07.644’	50°02.548’	9.4	259.5	Moderate	2	vertical	NA	BD	BD	BD	NA
Y12	18/10/2017	12:00	12°33.091’	52°40.157’	11.0	262.8	rough	2	vertical	BD	BD	BD	BD	NA
Y12b	18/10/2017	14:41	12°37.539’	53°03.779’	10.9	263.4	rough	3	vertical	BD	BD	BD	BD	NA
Y13	19/10/2017	13:00	13°59.327’	55°42.738’	10.0	259.4	rough	2	vertical	BD	A	BD	NA (A)	NA
Y14	20/10/2017	13:00	15°57.628’	59°41.706’	8.4	266.7	Slight	2	no	BD	BD	BD	A	NA
Y15	21/10/2017	14:05	14°33.295’	61°35.655’	9.4	262.2	Slight	3	no	BD	A	A	NA (A)	NA(A)

### Sea state, wind, current datasets and raft disaggregation state

In order to interpret the *Sargassum* aggregation distribution and structure with respect to their dynamic environment, we also examined the sea state, the surface wind and surface current, as well as the disaggregation state of the *Sargassum* rafts (absent, horizontal or vertical) (Tables 2 and 3). Vertical disaggregation means that *Sargassum* thalli are mixed down in the water column. Horizontal disaggregation means that *Sargassum* thalli are scattered at the surface. The sea state data came from ship deck observations (Douglas scale). No *in situ* current measurements were taken during the cruise so we used current fields at 1/12° provided by the Operational Mercator global ocean analysis and forecast system PSY4V3R1 [57]. The 10 m wind vectors were provided by the ECMWF HRES forecast product [58] at 0.09° resolution. *In situ* wind measurements, consistent with wind vector products, were also reported in the Beaufort scale for each station. Nevertheless, to be consistent between *in situ* and satellite datasets, we only used the wind vector products in this study.

## Results

### Wind and sea conditions during cruises

During the *West Atlantic* cruise, the trade winds were rather constant in speed (from 3.6 to 9.2 m s^-1^, average 7.6 m s^-1^) and direction (NE), with a sea state generally slight to moderate (Table 2). During the *Transatlantic* cruise, the trade winds were more variable, with speeds from 3 to 11 m s^-1^ (average 6.6 m s^-1^) and sea state from smooth to rough (Table 3).

### *In situ Sargassum* raft observations

Whatever the raft type, we observed floating *Sargassum* every day on the sea surface along the ~ 10 000 km tracks of the two cruises (except near the French Guiana coast on the first day of the *West Atlantic* cruise, close to station S1). *Sargassum* was the only floating algae observed at offshore stations but has been observed mixed with other marine vegetation near the Caribbean coasts (*Syringodium filiforme* Kützing, *Thalassia testudinum* Banks & Solander ex König). The *Sargassum* raft types determined for each station (42 in total) are reported in Tables 2 and 3 and Fig 1. Tables 2 and 3 also includes additional information on possible raft disaggregation, wind conditions, sea states and on satellite data availability. Furthermore, pictures taken at each station and satellite images are provided in the supplementary material (S2 and S3 Figs).

Concerning sightings at stations, floating *Sargassum* thalli were absent at three stations only (Fig 1): close to the French Guiana coast (station S1), and in the center of the Tropical North Atlantic (stations Y10, Y11). These stations highlight the occurrence of low *Sargassum* density areas. Nevertheless, for stations Y10 and Y11, we observed floating *Sargassum* of type 1 or 2 later in the day (i.e. station Y11b).

As already mentioned, we frequently observed *Sargassum* rafts of different types close to each other at the same station, but only the highest type observed for each station is reported in Fig 1 and Tables 2 and 3 and discussed hereafter. Type 1 represented 14% of the observed rafts, and was mainly found in the Sargasso Sea and in the central Tropical North Atlantic (-35°E to -50°E). Types 2 and 3 were the most common (55% among all stations) and were observed everywhere in the western and eastern Tropical Atlantic, near the Caribbean and African coasts. These *Sargassum* windrows were always aligned with the wind direction. *Sargassum* windrows with larger patches (Type 4), reaching tens of meters, were also observed a few times (12%). These large patches were often observed downwind of windrows, forming a front of *Sargassum* large patches with a trail of *Sargassum* windrows. We observed very large quasi-circular rafts with diameters ranging from ~ 50 m to several hundred meters (type 5) in 12% of the cases. Two of them were observed during the *West Atlantic* cruise, off Barbados (S9), and in the Guadeloupe Island coastal area (S24). Three were observed during the *Transatlantic* cruise, mainly near the African coast at about -20°E (Y02, Y04b), and one further west at about -35°E (Y07b). These large rafts were always observed alone (i.e. without other Type 5 rafts) in our field of view but can be downwind of—and/or surrounded by—*Sargassum* windrows. It may be noted that additional observations, made occasionally between stations by Scientifics that were present on the ship’s deck (and not reported in Tables 2 and 3), were consistent with the percentage of each *Sargassum* raft type observed at station locations and described above.

Scuba diving observations showed that *Sargassum* rafts have a typical thickness generally ranging from 10 to 50 cm. The large raft encountered off Barbados was, however, about 7 m thick (S9; based on scuba diving observation).

**Fig 3:**
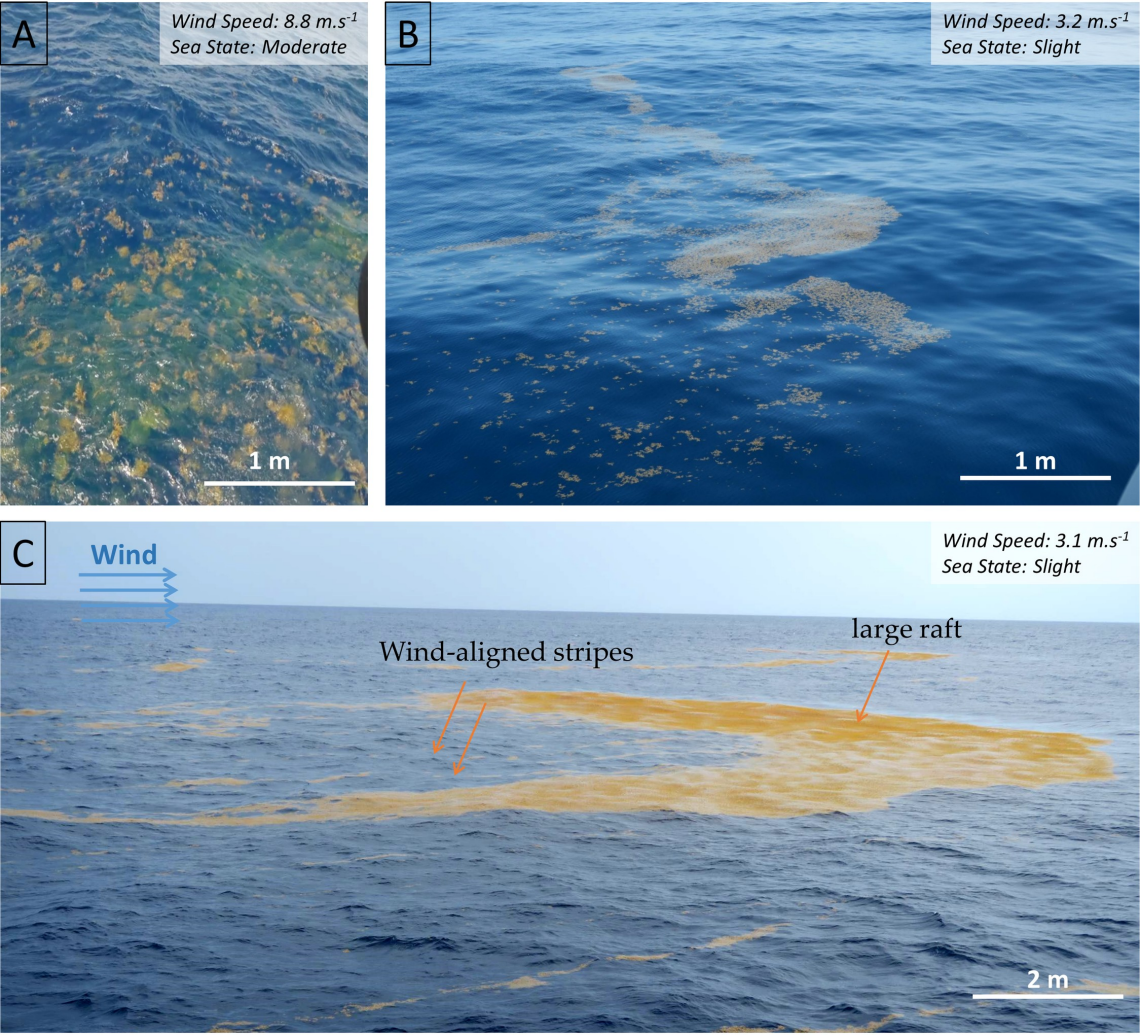
Three cases of *Sargassum* raft disaggregations. A) Vertically and horizontally disaggregated windrows at station S16. B) Horizontally disaggregated windrows observed at station Y03. C) Type 4 rafts in the process of disaggregation into windrows observed at station Y04b. In C, the wind was blowing from the left of the image.

During both cruises, we observed disaggregated rafts. Three typical cases are highlighted hereafter. During rough sea and strong wind conditions, we observed disaggregated rafts with some *Sargassum* mixed down to a depth of about 1 m (Fig 3A, stations S16-S18, Y11b-Y13). Under light wind conditions, some *Sargassum* rafts were observed to be horizontally disaggregated with scattered *Sargassum* thalli (Fig 3B, stations Y03-Y03b). Finally, a large raft (type 4) was observed curved into a crescent shape, with the concave side facing the wind direction and the convex side disaggregated into windrows (Fig 3C, close to station Y04b).

### Multi-scale satellite observations of *Sargassum* aggregations

**Fig 4:**
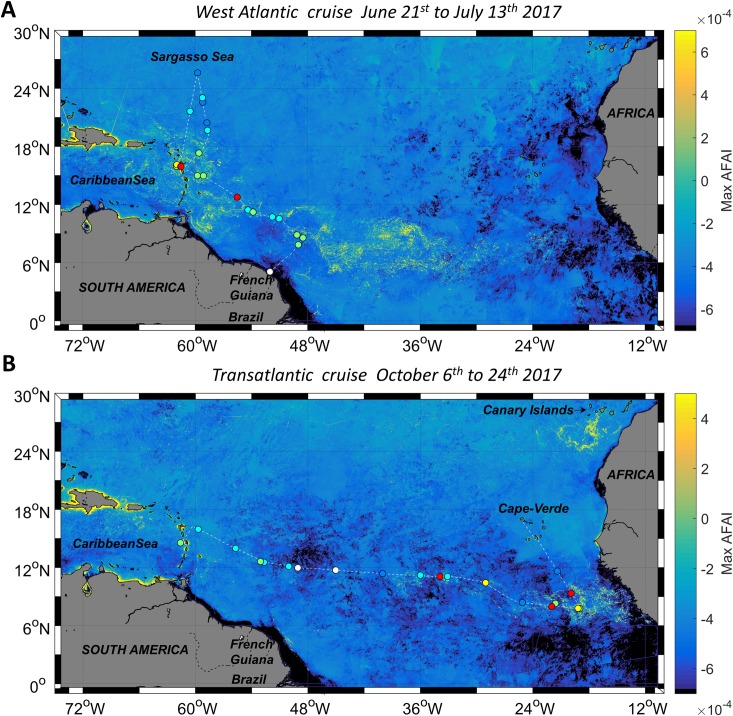
**MODIS AFAI composites for the *West Atlantic* (A) and *Transatlantic* (B) cruises (1 km resolution).** Composites were computed using all the available MODIS AFAI images for a zone encompassing the cruise track over its duration. The cruise track is represented by a white dashed line and stations are symbolized by dots with colors indicating the observed raft types (as in Fig 1). The detection area observed north of 24°N near the Canary Islands in the *Transatlantic* cruise MODIS composite is attributed to the presence of *Trichodesmium* that has a red edge signature similar to *Sargassum*. Observations of abnormally high concentrations of these cyanobacteria were indeed reported on Canary Islands shores and coastal waters in summer 2017 by [59].

**Fig 5:**
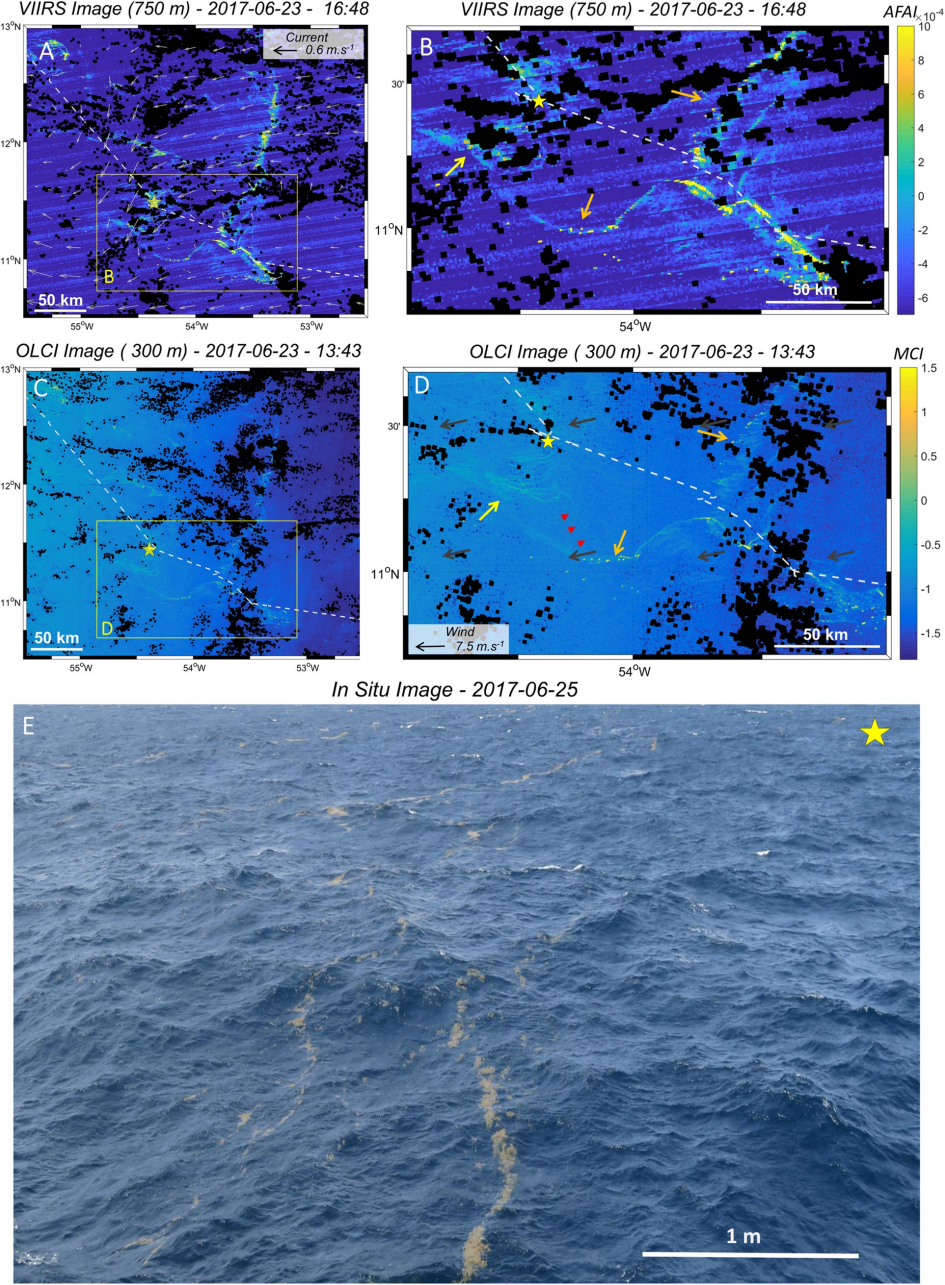
Satellite images (VIIRS and OLCI) and *in situ* pictures of *Sargassum* aggregations and rafts observed east of the Caribbean arc during the *West Atlantic* cruise (S8). A) VIIRS-AFAI images of June 23^rd^, 2017, with modeled currents shown as light grey arrows. B) Zoom of A) on a large *Sargassum* filament. C) OLCI-MCI image of the same scene. D) Zoom of C) on a large *Sargassum* filament with modeled wind shown as grey arrows. Note the short time lag between VIIRS acquisition time (16:48) and OLCI (13:43). Orange and yellow arrows indicate some *Sargassum* features discussed in the main text. Red arrows indicate small and isolated *Sargassum* filaments observed with OLCI but not with VIIRS. The cruise track is indicated as a white dashed line. E) *In situ* picture illustrating type 2 *Sargassum* filaments, taken two days after the satellite images acquisition at station S8 (indicated by a yellow star on all panels).

With their different spatial resolution and coverage, the four satellite sensors VIIRS, MODIS, OLCI and MSI were able to capture nested scales of aggregation. Hereafter, we use and compare the abilities of sensors to describe floating *Sargassum* aggregations at various scales. First, at the whole Tropical North Atlantic scale (Fig 4), then at large (>1 km to hundreds of km) and small scales (>10 m to 1 km) over three typical cases (Fig 5 to Fig 7). *In situ* observations made during the two cruises in the same area and time period (+/- 3 days) help in interpreting the satellite detections.

**Fig 6:**
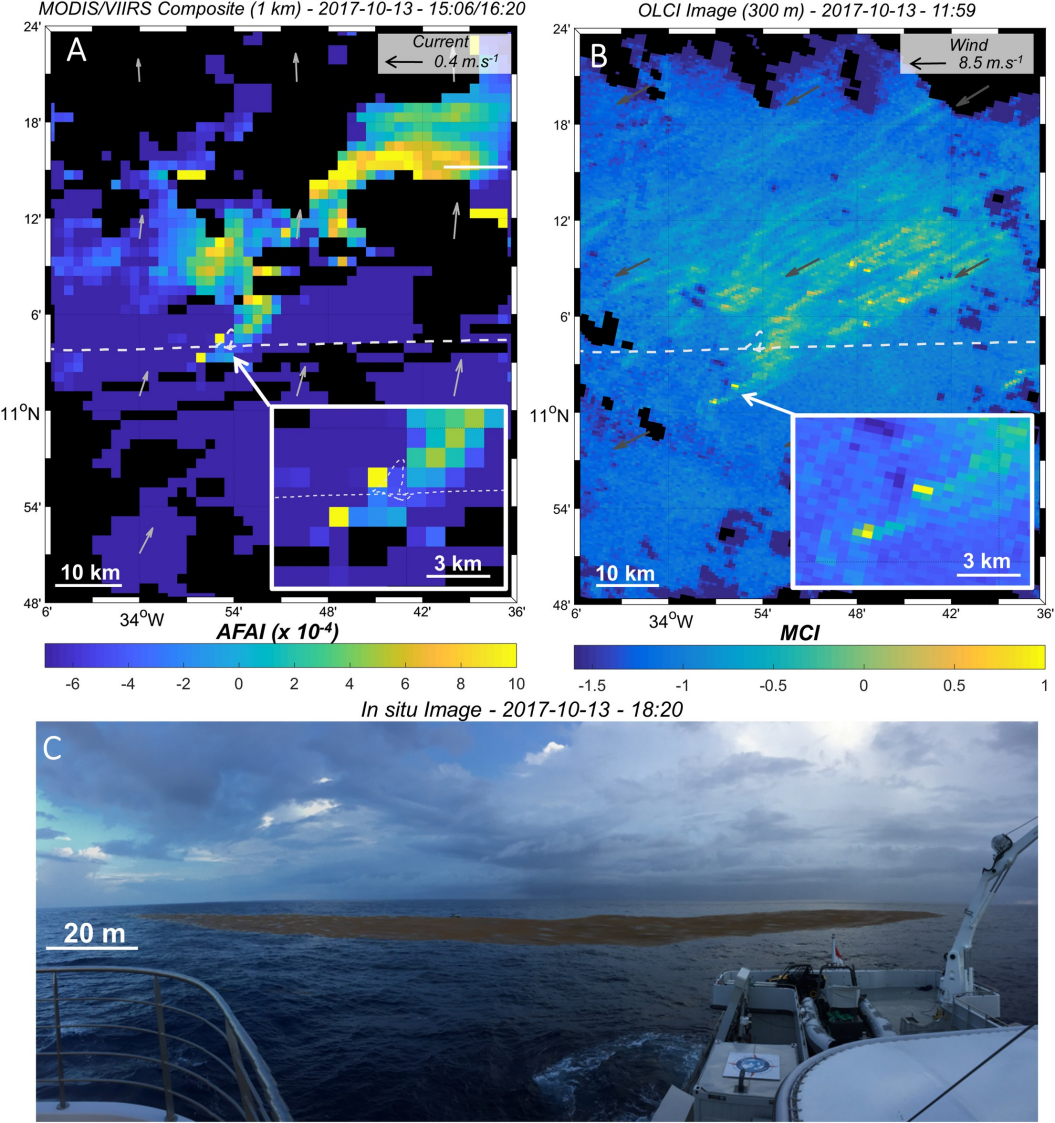
Satellite images (MODIS-VIIRS and OLCI) and *in situ* pictures of *Sargassum* aggregations and rafts observed in the middle of the Tropical North Atlantic during the *Transatlantic* cruise (Y07b). A) MODIS and VIIRS -AFAI composites, with modeled currents as light grey arrows. B) OLCI-MCI image with modeled wind as dark grey arrows. The dense *Sargassum* area (11°N- 33°W) on MODIS-VIIRS composite is composed of *Sargassum* filaments and large aggregations as shown in OLCI-MCI. Zooms on two large *Sargassum* aggregations are shown in lower right insets. The cruise track is indicated as a white dashed line. C) *In situ* picture of one *Sargassum* type 5 raft observed on A) and B) zooms, taken at station Y07b about two hours after the VIIRS and MODIS images.

**Fig 7:**
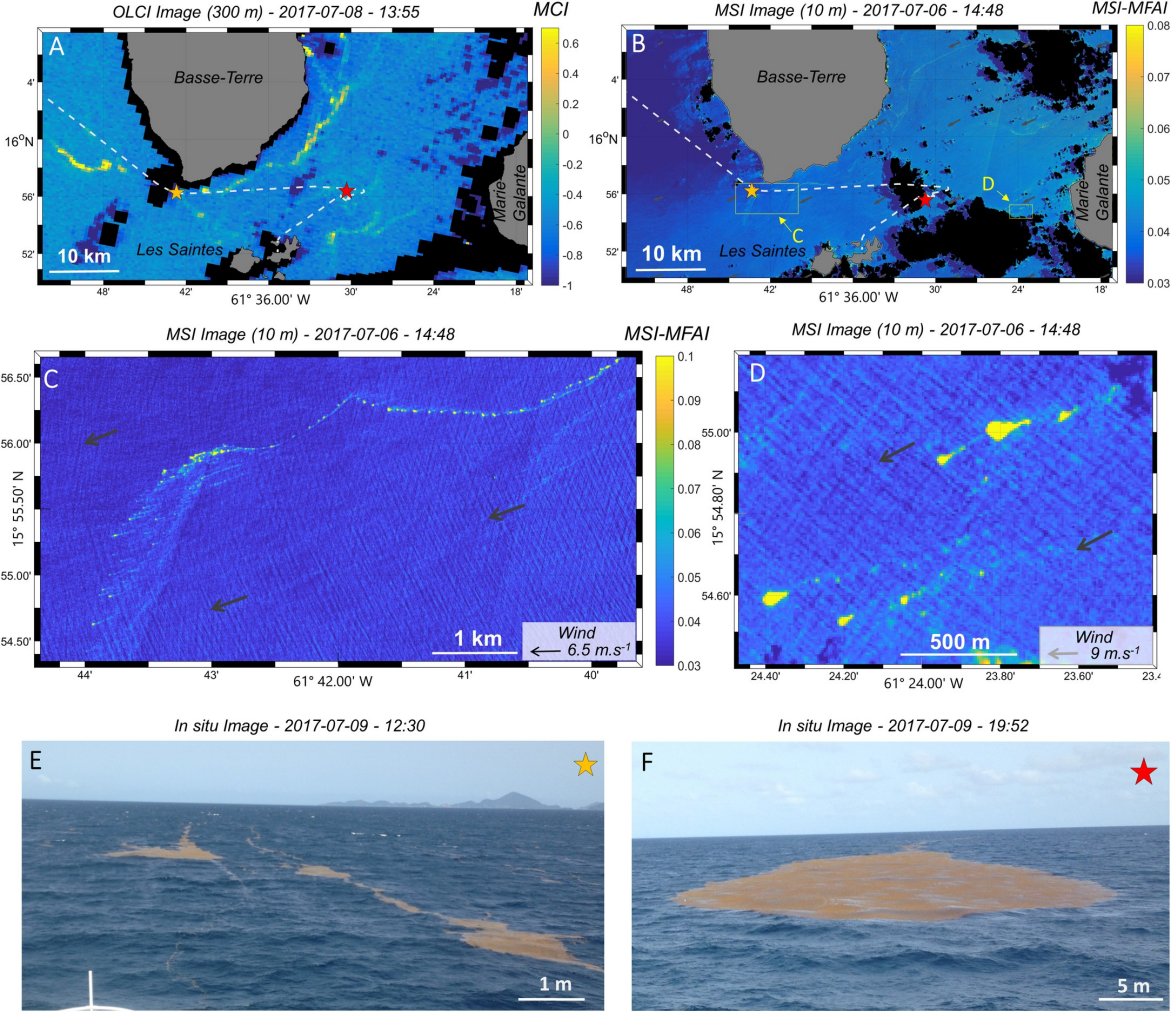
Satellite images (OLCI and MSI) and *in situ* pictures of *Sargassum* aggregations and rafts observed south of Guadeloupe Island during the *West Atlantic* cruise (S24a). A) OLCI-MCI image of July 8^th^. B) MSI-MFAI image of July 6^th^. C) and D) Zooms of MSI-MFAI image corresponding to the yellow rectangles on B) with modeled wind as dark grey arrows. E) and F) *In situ* pictures of Type 4 and Type 5 *Sargassum* rafts taken between station S23 and station S24a and at S24a, respectively (indicated by orange and red stars on A) and B)).

As *Sargassum* was the only floating algae observed during the two cruises, we assume that the satellite algae indexes were detecting only *Sargassum* in the entire Tropical Atlantic [0°-24°N], including Caribbean Islands coastal areas were others floating vegetations were scarce.

### Tropical North Atlantic scale

The MODIS AFAI composites computed for the duration of both cruises (24 days during June-July 2017 and 18 days during October 2017) give an overview of the occurrence of *Sargassum* during these two cruises, and highlight the large-scale *Sargassum* distribution in the Tropical North Atlantic, for two different seasons of the same year (Fig 4). MODIS AFAI composites showed that *Sargassum* was detected along the whole of the cruise track, except in the Sargasso Sea during the *West Atlantic* cruise (Fig 4A) and in the middle of the Tropical North Atlantic (-35°E to -50°E) during the *Transatlantic* cruise (Fig 4B). In June-July 2017, the highest cover of *Sargassum* was detected from off the coast of Brazil to the Caribbean islands, whereas in October 2017 *Sargassum* was mainly detected close to the African coast (-17°E to -35°E) (Fig 4A and Fig 4B). These satellite observations show a distribution pattern consistent with the *in situ* observations reported in Fig 1, with type 2 to type 5 *Sargassum* rafts reported in the areas with highest *Sargassum* cover. Type 1 (i.e., isolated *Sargassum*) was mainly recorded in the Sargasso Sea and in the central Atlantic, areas where satellite imagery does not detect *Sargassum*, at least not at 1 km resolution.

### From large to small scales

MODIS and VIIRS sensors were also able to capture *Sargassum* aggregation distribution and structure at 1–100 km scale (Fig 5 and Fig 6). At this scale, *Sargassum* aggregations were often detected as elongated filaments hundreds of km long (Fig 5A and Fig 5B), but also as large compact areas of thousands of km^2^ (Fig 6A).

MODIS and VIIRS images (1 km and 750 m resolution, respectively) were compared with the higher resolution images provided by the OLCI sensors (300 m) over the same zone and on the same day (Fig 4A–4D and Fig 5A and 5B). In both images, the OLCI sensor is able to capture the *Sargassum* signatures. Moreover, OLCI reveals *Sargassum* aggregation sub-structure (hundred-meter scale; see after) and highlights small and isolated *Sargassum* filaments (e.g., red arrows on Fig 4D) that are not discriminated in the coarser VIIRS and MODIS images.

OLCI images showed that the km-scale filaments (orange arrows on Fig 5B) and large compact areas (Fig 6A) of *Sargassum* detected with VIIRS and MODIS were in fact formed by a front of small parallel filaments aligned with the wind direction (Fig 5D and Fig 6B). Most of these small filaments started with one or two pixels of high *Sargassum* coverage (high MCI index, yellow color), located downwind of the filament, probably representing one or several large *Sargassum* patches. Two of these small areas of high *Sargassum* coverage pixels (~2 pixels) detected by OLCI (zoom on Fig 6B) were observed at sea during the *Transatlantic* cruise, and corresponded to two large rafts of Type 5, the largest of them measuring ~250 m x ~50 m (Station Y07b, Fig 6C). These rafts were also detected by VIIRS but were hardly identifiable as they are represented as a single isolated pixel (zoom on Fig 6A).

Similarly, the diffuse *Sargassum* detections observed on the west side of the main *Sargassum* aggregations seen by VIIRS (yellow arrow on Fig 5B) are formed by weak (low algae index) and narrow *Sargassum* filaments parallel to the wind direction on the OLCI image (yellow arrow on Fig 5D), with spacing close to pixel size (300 m). Several clusters of narrow *Sargassum* windrows (type 2), a few meters apart (5–10 m), were observed at this location during the *West Atlantic* cruise (Fig 5E).

Close to the coasts, MSI images (10 m resolution) were available. Here, we compared OLCI and MSI images acquired over the south of Guadeloupe Island at only two days interval (Fig 7). MODIS images were also available for this area, but coastal pixels were strongly affected by straylight contamination (i.e., contamination from adjacent land pixels [60]), precluding any *Sargassum* detection (S2 Fig [21]). The OLCI image showed *Sargassum* filaments, one or two pixels wide, wrapped around the Basse-Terre coast and between Les Saintes and Marie-Galante Islands (Fig 7A). *Sargassum* detections were hardly distinguishable on the MSI image of this region (Fig 7B) because of their narrowness, but some can be observed in the same areas as those detected on the OLCI image. This suggests that *Sargassum* filaments observed on the OLCI image were already present on the MSI image acquisition two days earlier. The MSI high spatial resolution makes it possible to capture the subpixel details of filaments seen on the OLCI image. Once again, these filaments were actually formed by several shorter and narrower filaments aligned with the wind direction and associated with a front of large patches (Fig 7C). Drop-shaped large *Sargassum* aggregations were also detected by the MSI sensor (Fig 7D). These MSI detections matched *in situ* observations taken in the same area 3 days later with *Sargassum* rafts of Type 4 (Fig 7E) and Type 5 (Fig 7F).

## Discussion

### *Sargassum* rafts and typology

The *Sargassum* raft shapes, observed from the ship deck during the two cruises and classified according to our typology are consistent with historical data mostly collected in the Sargasso Sea ([23,25], summarized in [22]). These historical observations reported more or less circular rafts reaching ~ 50 m across, corresponding to Types 4 and 5 in our typology (Fig 2). However, the most commonly reported description is “small clumps of 10–50 cm diameter, which tend to line up in the direction of the wind in rows spaced 20–50 m apart” [22,23]. This corresponds to our Types 2 and 3 that were the most common types during the 2017 cruises (Fig 1). To our knowledge, historical observations did not report very thick *Sargassum* rafts such as those observed during the *West Atlantic* cruise (S9). This observation may thus be unusual, but caution is needed before drawing conclusions as raft thickness, if observed, was rarely recorded in historical reports. In addition, most of our sampling stations were targeted where satellite indexes showed significant aggregations. This differs from previous works [22,23,25,34] that used systematic net tows along the ship's route. This difference and the reduced spatial overlap hamper a quantitative comparison with historical data.

The typology proposed here goes beyond simpler descriptions already proposed in literature [22,61,62] and in [37] (i.e., three types: lines of *Sargassum*, mats and scattered clumps). It is easier to use for any observer, whether scientists or not, thanks to a numbered scale and drawings. It could be included in the already available *Sargassum* reporting website [37,63]. Moreover, the five types proposed in this typology give size information missing in previous descriptions, with types ordered following raft coverage (e.g., Type 3 < Type 4 < Type 5, a raft area coverage estimation for each raft type is given in S1 Table, see discussion on satellite detection limit following) and thus *Sargassum* quantity. Although raft types can be variable because of changes in current and wind, the available *Sargassum* quantity will be rather stable for a duration shorter than growing and sinking timescales. We thus argue that this typology can provide useful information for *Sargassum* biomass estimations. Nevertheless, to accurately estimate the quantity through the types, additional information is needed, such as windrows spacing, average width and length, and frequency of patches. The best means to access this information would be airborne imagery.

### *Sargassum* aggregation from small to large scale: Relationship with wind and currents

The process of *Sargassum* aggregation occurs across a wide range of scales. At small scale (<1 km), floating *Sargassum* probably aggregate under wind induced drift and current (Langmuir circulation; [64]) to form *Sargassum* rafts, mainly windrows and patches of different sizes (Fig 2). At larger scales, these *Sargassum* rafts still probably aggregate following persistent currents, and form clusters of *Sargassum* aggregations reaching hundreds of km in length (e.g., Fig 5). Satellite images of different resolutions of the same scene (e.g., Fig 5, Fig 6 and Fig 7) clearly evidenced that these scales are nested. Every filament seen at 1 km resolution was made of several smaller aggregations detected at 300 m, and so on, down to the *in situ* scale of the windrows, each row being a few cm wide and spaced from 1 to several tens of meters apart.

During both cruises, *Sargassum* windrows (Types 2, 3, but also associated with Types 4, 5) were ubiquitous, consistent with the significant wind speed occurring at most stations [64]. We observed no significant relationship between the raft types and the wind speed at the time of observation (Kruskal-Wallis test, p = 0.09). As rafts are constantly changing, this may be due to a time offset between the raft observation and the wind effect. Most of the rafts were only half a meter thick and the *Sargassum* were not intertwined. Even in large dense rafts (type 4–5), air bubbles from scuba divers easily dispersed the *Sargassum*, suggesting that disaggregation could be a rapid process. For example, we observed a disintegrating large raft under a wind of 3.1 m s^-1^ at station Y04b (Fig 3). Windrows were formed upwind of the raft that progressively disintegrated. This observation is fully consistent with the 'raft and trail' shape reported by [38], observed under a wind speed of 5 m s^-1^. Nevertheless, large rafts (types 4–5) were observed at wind speeds of 4–11 m s^-1^. This may differ from historical observations [22,25] which report that large rafts were mostly observed during calm conditions. In addition, *Sargassum* rafts were observed horizontally disaggregated during light wind conditions, possibly due to the relaxation of the Langmuir cells maintaining the windrow pattern [64], and supporting the significant effect of wind on raft cohesion (Fig 3B). Wind, through wind-induced wave mixing, can also lead to the vertical disaggregation of *Sargassum* rafts into the water column. This disaggregation seems to appear only under high wind and rough sea conditions (wind speed > 7 m s^-1^, sea-state > moderate), and can lead to *Sargassum* being mixed into the water column down to a depth of about 1 m (Fig 3A). The 8 km horizontal resolution of the modeled surface current used in this study is not suited to discuss the current small scale effect on *Sargassum* distribution. Moreover, we were unable to record *in situ* small scale current measurements during the cruises to compare them with *in situ* observations. Additional observations, such as repeated *in situ* and satellite/airborne observations of one particular raft, associated with high frequency measurements of wind and local current, could provide valuable information enabling a better understanding of the aggregation and disaggregation processes, as well as the dynamics that control the transition from one raft type to another.

From space, 1 km resolution sensors showed aggregations with an elongated structure up to hundreds of km in length (Fig 5) or forming compact areas tens of km wide (Fig 6). These aggregations may arise from (sub)mesoscale circulation features, e.g. eddies [65], as observed for other satellite-derived quantities, such as surface Chl-*a* [66,67]. In particular, convergence areas estimated by Finite Scale Lyapunov Exponents could be favourable to *Sargassum* aggregation [66]. However, no matching patterns were found between 1 km *Sargassum* distribution and the synchronous modeled current field for the examples illustrated in Fig 4A–4C and Fig 5A. This may be due to the low number of available examples, as cloud cover was heavy, as well as to the coarse resolution (8 km) of the modeled current field with respect to the typical aggregation size.

### Basin scale *Sargassum* distribution

In the present study, the *Sargassum* distribution was mapped using satellite observations for two seasons of 2017 (spring and autumn) over the whole Tropical North Atlantic, from the Caribbean islands to the African coast [29]. The large-scale distribution patterns observed from space during the period of the two cruises show that *Sargassum* was not only present off Brazil and near the Caribbean islands during spring and summer but extended eastward as far as the African coast. In October, the highest density of *Sargassum* aggregations was observed off Guinea and Sierra Leone. This distribution was validated by *Sargassum in situ* observations made during the two cruises in spring and autumn 2017. Furthermore, the UNEP Abidjan convention reported beaching of *Sargassum* along the shorelines of Africa in the winter of 2011 [68]. This distribution pattern is consistent with the 2011 *Sargassum* distribution mapped with MERIS [29], and with the *Sargassum* distribution mapped with MODIS in the western part of the Tropical North Atlantic from 2011 to 2015 [30].

In the Western Tropical North Atlantic, the *Sargassum* spatial extent observed in June-July 2017 is one of the highest observed since 2011 and seems to be intermediate between the ranges observed in 2014 and 2015 [30]. This is consistent with the high *Sargassum* biomass stranding on the Caribbean coast in 2017 (AO, TT, SR, TC, AB, JB, DA observations July 2017). In 2018, massive strandings have also been reported since January [69]. Satellite imagery bulletins reported for 2018 the highest *Sargassum* spatial range ever recorded since 2011, with quantities that exceed the 2015 and 2017 records [39,70]. This follows the inter-annual variability pattern observed these past seven years: an alternation of two years of high abundance, followed by one year of lower abundance, associated with an overall increase in *Sargassum* abundance since 2011 [39].

*Sargassum* presence and abundance in the Tropical North Atlantic is most likely linked to both transport and growth processes. [71] showed that circulation alone could not explain the satellite-derived *Sargassum* distribution pattern at the seasonal scale. Adding a simple *Sargassum* growth representation in their model, they were able to maintain a realistic high abundance in the tropics.

MODIS composites did not detect *Sargassum* inside the Sargasso Sea either in June-July or in October 2017, at least as far as 30°N. This agrees with the very low abundance to quasi-absence of *Sargassum* recorded *in situ* during the 2017 *West Atlantic* cruise.

### Match between *in situ* and satellite observations

**Fig 8:**
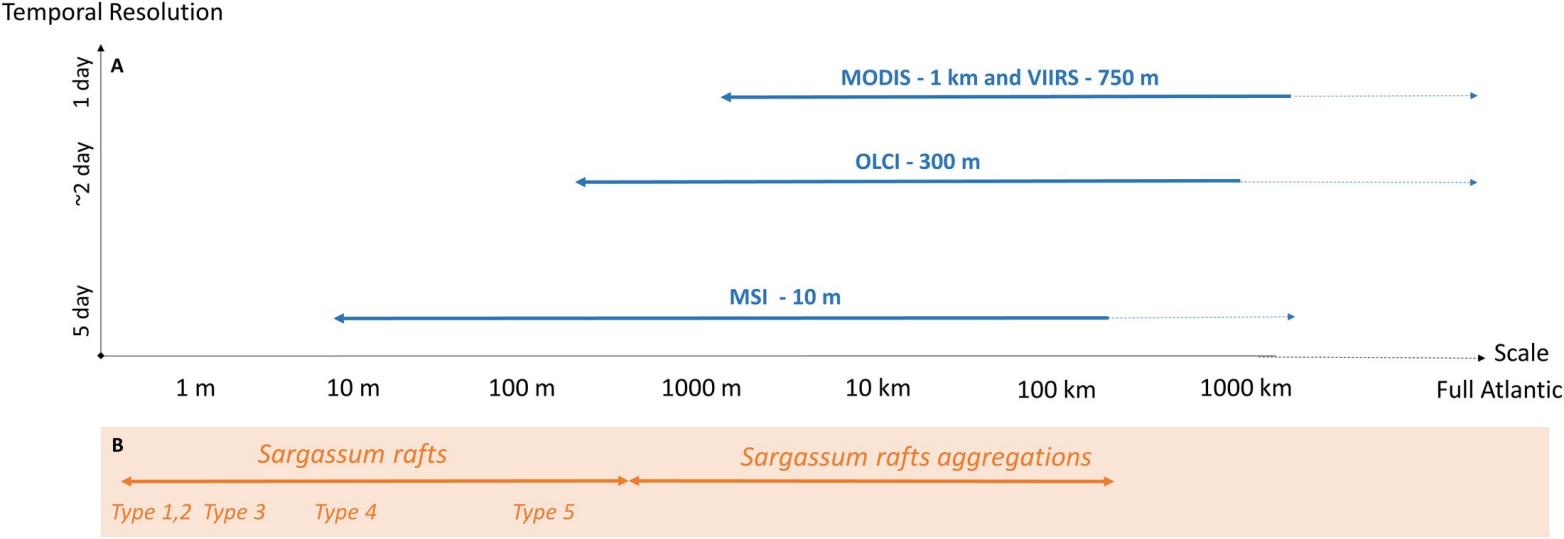
**Capability of the MODIS (Aqua & Terra), VIIRS, OLCI (S3A & S3B) and MSI (S2A & S2B) satellite sensors in terms of spatial resolution, temporal resolution and spatial coverage to describe *Sargassum* aggregations at various scales (B), and comparison with *in situ Sargassum* raft scales (B).** Solid lines on (A) indicate the different scales attained by the sensor with one image. Dotted lines indicate the maximum spatial coverage reached by each sensor, with a frequency limited by its temporal resolution. Note the logarithmic scale of the x-axis.

Match-ups (i.e. observation of the same area at the same time) between *in situ* and satellite observations were rare in our study, for several reasons. Firstly, the timing of the stations was not decided on the basis of satellite overpass but of the presence of *Sargassum*. Secondly, cloud coverage was high and observations not always available because of the satellite temporal resolution. Thirdly, because *Sargassum* distribution is not homogeneous, a full match-up between *in situ* and satellite observations would require the *in situ* observation of the entire pixel area (Fig 8). For 1km, 750m and 300m pixel, ship observations only provide partial coverage of the pixel. Only the 10m pixel resolution would allow an overlap and a full comparison between *in situ* and satellite observations, but no same day match-ups were available with MSI images. Increasing the frequency of very high resolution satellite (such as MSI resolution or higher) observations or using airborne imagery, in order to increase the number of match-ups, should make it possible to bridge *in situ* and satellite observations.

Nevertheless, satellite observations made in the same areas as *in situ* observations within a ± 3 days windows seem consistent with *in situ* observations in terms of *Sargassum* aggregation structure and abundance. Weak and narrow *Sargassum* filaments (low floating algae index) observed with OLCI are consistent with the *in situ* observation of Type 2 *Sargassum* rafts observed 2 days earlier, while bright pixels (high floating algae index), mainly observed at the front of filaments, probably correspond to the presence of *Sargassum* patches at the front of the rafts, as often observed *in situ* (type 4–5). However, no large aggregation extending over several km as suggested by MODIS and VIIRS observations were observed *in situ*. *In situ* and higher resolution satellite imagery suggested that these “very large rafts” are in fact formed of several, nested smaller scale *Sargassum* aggregations. Although these equivalences between *in situ* and satellite observations have to be considered with caution because of the high variability in raft shape and size, we think that they can help to better interpret future *Sargassum* satellite observations in terms of *Sargassum* quantity and aggregation structure.

### Remote-sensing of *Sargassum*, a powerful tool?

Satellite and airborne imagery is widely used to detect and map floating marine vegetation at the sea surface, including benthic and pelagic *Sargassum* species, in many places worldwide (e.g. [21,29–31,38–45,47,48]. *Sargassum* is mainly detected using floating algae indexes based on their red-edge spectral property in the NIR (e.g. FAI [55], AFAI [30], MCI [54]). In this study, we compared floating algae products of four satellite sensors MODIS, VIIRS, OLCI and MSI, providing free and easy-access images at three different spatial and temporal resolutions. These floating algae products are concomitant with *in situ* observations of *Sargassum* rafts, validating the detection of *Sargassum*. Fig 8 summarizes the ability of these four satellite sensors to describe *Sargassum* aggregations across different scales. They are mainly constrained by their spatial and temporal resolution, as well as their spatial coverage. MODIS and VIIRS sensors are well designed to describe the *Sargassum* aggregation distribution patterns at Atlantic scale with a daily frequency, but we demonstrated that the OLCI sensor is better suited to describe their (sub-) mesoscale structure. However, OLCI observations are less frequent: only 45% of the stations during the two cruises were covered using this sensor (Tables 2 and 3). However, this will increase with the recently launched OLCI/Sentinel-3B sensor, which will increase its temporal resolution up to ~2 days (Fig 8). The MSI sensor is also able to provide a fine description of the *Sargassum* aggregation structure, and partly resolves the *in situ* scale (Types 4 to 5), but only in coastal areas and only every 5 days. To get the full picture of *Sargassum* aggregations, we are lacking 0.5–10 m observations, which can be acquired through dedicated images from aircraft, drones, or very high-resolution satellite sensors such as SPOT (1.5–6 m) or Pléiades (0.5–2 m). A better understanding of aggregation and disaggregation processes and dynamics would also benefit from a higher temporal resolution and spatial coverage, especially for the high spatial resolution sensor MSI.

Nevertheless, some limitations have to be considered when using satellite sensors to detect and describe *Sargassum* aggregation:

The *Sargassum* detection limit is constrained by the ratio between the spatial coverage of *Sargassum* aggregations and the spatial resolution of the satellite sensors. [30] indicated that the detection limit for *Sargassum* aggregation with the MODIS/AFAI index is a fractional coverage of 0.2% of the MODIS 1-km pixel size. No data were available in our dataset to test this limit, as it would require visual estimation of the *Sargassum* coverage within a 1 km^2^ area collocated with MODIS pixel, which is difficult to access from a ship. We reported in S1 Table the fractional coverage range reached by each *Sargassum* raft type for the MODIS, VIIRS and OLCI spatial resolution. Fractional coverage for the MSI sensor was not computed. The size of the MSI pixel is of the same order as those of the *Sargassum* rafts, thus fractional coverage would be highly dependent on the *Sargassum* rafts distribution in the area covered by the MSI pixel. Key information such as windrow width, windrow spacing and distance between patches was not always recorded during the cruises as they are highly variable, thus typical values were used to estimate the fractional coverage. For Types 1 to 4, we considered that the *Sargassum* raft type was constant over the pixel. S1 Table shows that based on these typical values and on the detection limit of 0.2%, all types, except Type 1, should be detected by the four sensors. Types 2, 3 and 4 show a fractional coverage of about 3%, 4% and 6% respectively for a windrow width of 30 cm and a spacing of 10 m. This fractional coverage can decrease to 1% for a windrow width of 10 cm. A Type 5 raft with of 250 m x 50 m, such as those encountered during the *Transatlantic* cruise, has a fractional coverage of 1% for MODIS, 2% for VIIRS and 14% for OLCI. Considering a detection limit of 0.2% for all sensors, the minimum size for a Type 5 raft to be detected would be 2000 m^2^ (~50 m × ~40 m) for MODIS, 1125 m^2^ (~25 m x ~45 m) for VIIRS and 180 m^2^ (~20 m x ~9 m) for OLCI. Environmental conditions are also a constraint on *Sargassum* detection. Highly diffusive atmospheric conditions such as the presence of aerosols in high quantity (e.g. Saharan dust is common in the Tropical North Atlantic region) can hamper *Sargassum* detection by decreasing their signal in the NIR [30,38,39,43]. Similarly, we observed during the two cruises submerged disaggregated *Sargassum* rafts (Fig 3A) due to strong wind-induced wave mixing. [38] and [43] estimated a 50% decrease of the signal in the NIR (~750 nm) when *Sargassum* were only 10–15 cm below the surface. Aggregation can also be a key process in *Sargassum* raft detection. Aggregation tends to reduce the proportion of water between *Sargassum* and to increase the number of *Sargassum* at the surface. This is supported by [39] who showed an increase of the *Sargassum* signal with the *Sargassum* biomass density.

Cloud and haze cover, common in tropical regions, as well as strong glint effect (i.e., specular reflection of solar irradiance on the sea surface), can lead to large gaps in satellite images that drastically reduce the satellite sensors’ nominal spatial coverage and frequency of observation (Fig 8). During the two cruises, 73% of the stations were not observed by the MODIS sensor, and 43% by the OLCI sensors (considering all available images at +/-1 day), because of cloud cover and high glint effect (see Tables 2 and 3 and S2 Fig for details on each station). The considerable data loss of the MODIS sensor is probably due to its lower spatial resolution and to the frequent saturation of the used spectral bands under hazy atmospheric conditions and glint. Several methods have been developed to overcome data gap issues, such as (i) image compositing over periods longer than 1 day and/or (ii) satellite data merging (e.g. Fig 5 and Fig 7A), offering the means to resolve some data gaps due to not persistent cloud cover or between satellite orbit tracks (e.g. Fig 7A). Nevertheless, analyses of the year 2017 showed that the MODIS AFAI coverage was only ~20% of the North Atlantic (0–50°N) each day (not shown in this paper). This coverage increases to 60% when compositing images over 7 days (weekly composite), and to about 75% over 30 days (monthly composites) (e.g., Fig 5). Note that near the coast, MODIS and VIIRS images can be affected by straylight contamination due to the proximity of land [60], precluding the use of these data at a distance to the coast of ~ 10 km or less.

The identification of the *Sargassum* algae from space is also not straightforward. Algae indexes used to detect *Sargassum* do not allow discrimination of *Sargassum* from other floating vegetations, from surface cyanobacterial and phytoplankton blooms, or from material such as plastic debris or oil slicks (e.g. [43,54]) that have a spectral signature in the NIR very similar to the red-edge. Discrimination can be done using the full spectra, including spectral bands in the blue-green wavelength range [43,72], but it is still limited by the multispectral resolution of current sensors. Near the coast, other floating vegetation (e.g. benthic vegetation, land vegetation) as well as plastic debris and oil can be present and mixed with *Sargassum* making their identification even more challenging. Off the coast, especially within the studied zone, other floating vegetation and materials are rare (only a few plastic debris items mixed in *Sargassum* rafts were observed during the cruises), and no blooms of *Cyanobacteria* (*Trichodesmium* spp.) were observed during the two cruises, allowing greater confidence to be attributed to *Sargassum* identification in this area (except north of 24°N near the Canary Islands (see Fig 4)). Nevertheless, interpreting algae index products, such as AFAI or MCI products, in terms of *Sargassum* detection calls for the analysis of additional information such as *in situ* observations or comparison with previous year *Sargassum* climatology (e.g., [29,30]). Otherwise, detection could be improved by the use of the future Hyperspectral sensors (e.g.,[42,43]).

A challenge for remote-sensing is now to quantify the *Sargassum* biomass present in the Atlantic and available for strandings in coastal areas, but the nesting of spatial scales makes this estimation complex. Comparison between observations at various scales (Fig 5 to Fig 7) shows that one '*Sargassum*-containing' satellite sensor pixel is actually composed of many sub-pixel sized *Sargassum* aggregations mixed with seawater. Estimating *Sargassum* abundance on the basis of a *Sargassum* coverage of 100% for all pixels would thus lead to an overestimation, especially for low resolution sensors. To overcome this factor, several methods have been used to estimate *Sargassum* fractional coverage and biomass density (g.m^-2^) within a satellite pixel (e.g., [30,38,39,43,44,47]). Most of these methods are based on a relation with the algae index value [30,39]. These estimations show that the mean *Sargassum* fractional coverage inside a 0.5° × 0.5° (~50 × 50 km^2^) box is mainly <0.1% (<6% for a 1 km-size MODIS pixel), with a biomass density in the surface layer mainly ranging from 0 to 2 g.m^-2^ (up to 100 g.m^-2^ within a 1 km-size MODIS pixel) [30,39]. Once again, these estimations could not be validated during the two cruises as biomass estimation was not possible over a full pixel area. Nevertheless, the estimation of *Sargassum* biomass was made *in situ* for 2 rafts of Type 4 and Type 5 during the West Atlantic cruise. *Sargassum* was collected with a 1m^2^ sieve from below during scuba diving. Then the collected mass was weighed on the ship. We recorded biomass density ranging from 5.26 ± 1.59 (S12) to 7.60 ± 0.77 kg.m^-2^ (S9) for the upper 0.5 m layer, which is close to the 3.34 ± 1.34 kg.m^-2^ measured *in situ* by [39]. Scuba-diving observations during the cruises showed that rafts were up to 0.5 m thick, so depth integrated biomass could be 5 times higher than that estimated for the upper 0.1 m. The composition of rafts was also very variable, with size (and biomass) of the *Sargassum* thalli ranging from a tiny air bladder to a 1-m fragment with axes, blades and bladders. In addition, the distribution and density of the thalli within the water column were heterogeneous. The biomass of the surface layer, like that estimated by satellite, may thus not be representative of a whole raft and only represent a a minimum estimation [39]. This calls for additional observations of raft thicknesses and *Sargassum* thalli composition and density in order to better estimate their variability and impact on biomass estimation.

## Conclusion

In 2017, two cruises dedicated to the study of *Sargassum* algae were carried out in the North Atlantic Ocean allowing the observation of numerous *Sargassum* rafts of various types offshore and near the coast. These observations led us to define a five-class typology that simplifies and standardizes *Sargassum* raft descriptions. These two cruises were routed using satellite observations enabling us to draw recurrent parallels between *in situ* and space-based *Sargassum* observations. However, match-ups (i.e. observation of the same area at the same time) between satellite and *in situ* observations were very difficult to achieve, mainly because of (1) the high cloud cover, (2) the difficulties of coordinating cruise stations and satellite overpass, and (3) the differences in observation scales (a few meters around the ship vs pixel size). A full comparison between satellite and *in situ* observations would require detailed information about observed raft types, such as the windrows width, spacing and patch frequency and size, over the whole satellite pixel area, which is not possible from a ship. This kind of information could be obtained and detailed comparisons made using airborne imagery or very high resolution satellite images and would be essential to validate satellite estimations of *Sargassum* fractional coverage and biomass. Other parameters limit *Sargassum* detection and quantification from space, such as the submerged depth of S*argassum* rafts and the raft thickness. Nevertheless, the comparison made in this work between data from satellite sensors and *in situ* observations, showed that free and easy-access satellite imagery datasets with various spatial, spectral and temporal resolutions provided by the MODIS, VIIRS, and the new OLCI and MSI satellite sensors (i) are able to successfully detect *Sargassum* abundance consistently with *in situ* observations, and (ii) are powerful tools to map the *Sargassum* distribution at large scale (e.g. North Atlantic scale), and to describe the (sub-)mesoscale structure of *Sargassum* aggregations, as well as the *Sargassum* raft shapes, sizes and patterns of distribution near the coast.

## Supporting information

S1 Fig **Comparison between MSI *Sargassum* maps computed using the (A) FAI and (B) MSI-MFAI floating algae index.** Zooms of A and B are presented in C and D respectively. Large stripe pattern in surface reflectance is observed across the FAI-derived map (A), caused by sensor parallax effect [56]. This precluded the attribution of a common threshold for the entire image, making it difficult to highlight all *Sargassum* aggregations (red arrows show *Sargassum* aggregation poorly visible on the FAI-derived map compared to the MSI-MFAI one).
(TIF)

S2 Fig Satellite sensors map (MODIS-A, MODIS-T, VIIRS, OLCI) and *in situ* pictures of *Sargassum* rafts observed for each station during the *West Atlantic* and *Transatlantic* cruise. For each station, the following information is reported (see Table 2): cruise name, date, time (UTC), Latitude (°N), Longitude (°W), Wind Speed (WS) and Wind Direction (WD) and Sea State (SS). Satellite sensors maps are computed with images acquired on the day of the station for MODIS and VIIRS and for the day +/- 1 day for the OLCI sensor and in an area of 0.2° x 0.2° around the station. The date, time and name of sensor observations are reported as well as the algae index used to map *Sargassum*.
(PDF)

S3 Fig MSI images matching stations S11 and S24b near Guadeloupe island (*West Atlantic* cruise) and station Y15 near Martinique island (*Transatlantic* cruise). Matches are made within a +/- 3 days interval and in an area of 0.1° x 0.1° around the station. For the three stations, the following information are reported (see Tables 2 and 3): cruise name, date, time (UTC), Latitude (°N), Longitude (°W), Wind Speed (WS) and Wind Direction (WD) and Sea State (SS). The date, time and name of MSI observations are reported.
(PDF)

S1 Table Fractional coverage estimates for each raft type and for MODIS, VIIRS and OLCI sensors. For these estimations, Type 1 is characterized by one Sargassum thalli of width SW and length SL each SS meters. Types 2, 3 and 4 are characterized by Sargassum windrows with a width ranging between 10 and 30 cm and windrow spacing varying between 10 and 2 m (the 2 m spacing is used to estimate the maximum fractional coverage limit reached by each type, as this spacing was never encountered over large areas during the two cruises). For Types 3 and 4, we considered patches of 1 mdiameter each 10 m for Type 3 and 4 m diameter each 50 m for Type 4. For Type 5, we considered only one big patch of a size similar to the Type 5 raft encountered during the *Transatlantic* cruise (station Y07b) (Patch Width (PW) = 50m and Patch Length (PL) = 250m).
(TIF)
