# Supplementary figures and images for: From In Situ to satellite observations of pelagic Sargassum distribution and aggregation in the Tropical North Atlantic Ocean
Source: PLoS One. 2019 Sep 17;14(9):e0222584. doi: 10.1371/journal.pone.0222584 (PMC6748567; doi:10.1371/journal.pone.0222584)

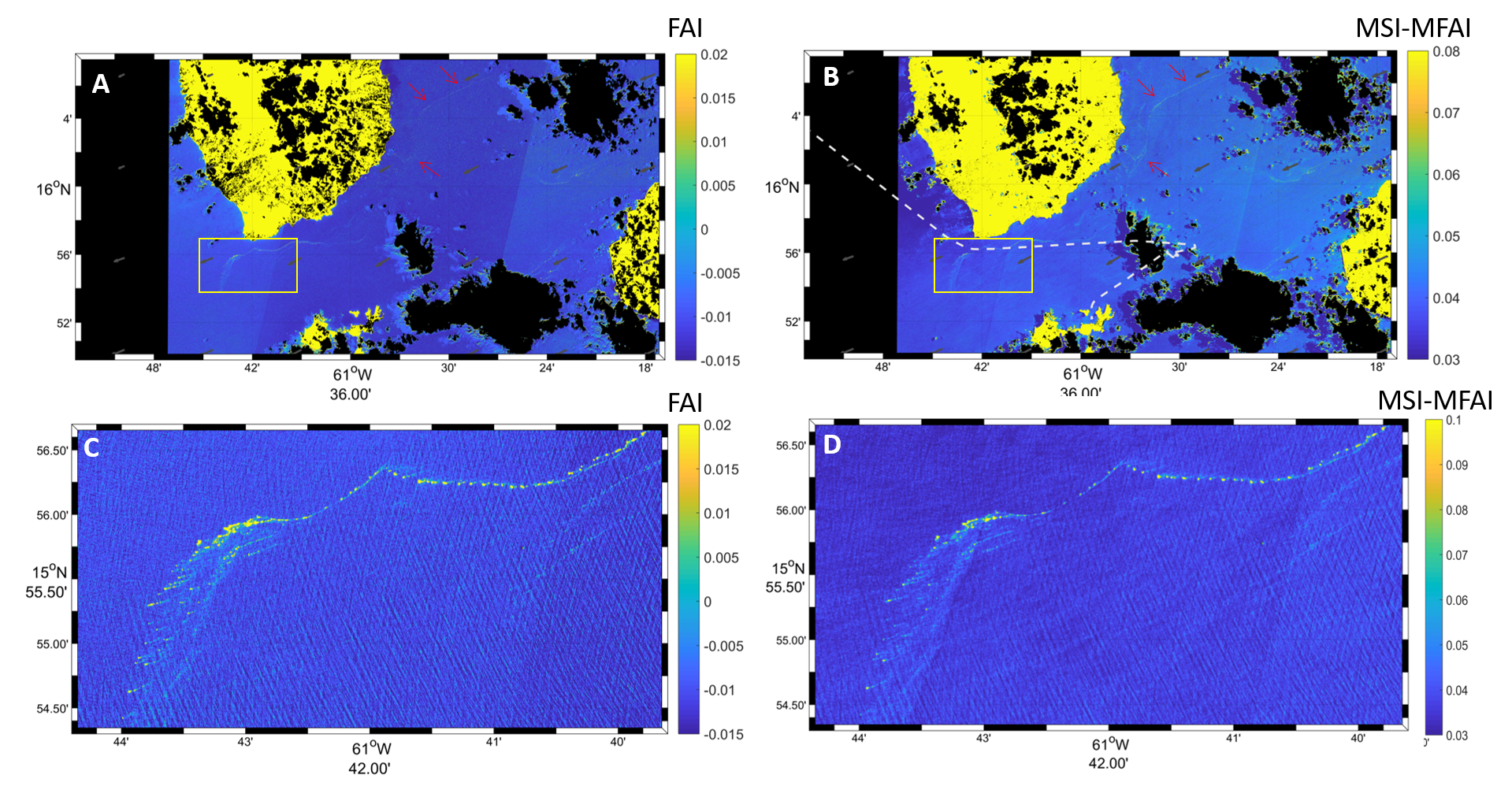

Supplement: S1 Fig — Comparison between MSI Sargassum maps computed using the (A) FAI and (B) MSI-MFAI floating algae index. Zooms of A and B are presented in C and D respectively. Large stripe pattern in surface reflectance is observed across the FAI-derived map (A), caused by sensor parallax effect [56]. This precluded the attribution of a common threshold for the entire image, making it difficult to highlight all Sargassum aggregations (red arrows show Sargassum aggregation poorly visible on the FAI-derived map compared to the MSI-MFAI one). (TIF) [file pone.0222584.s001.tif]

*MSI (10m)*

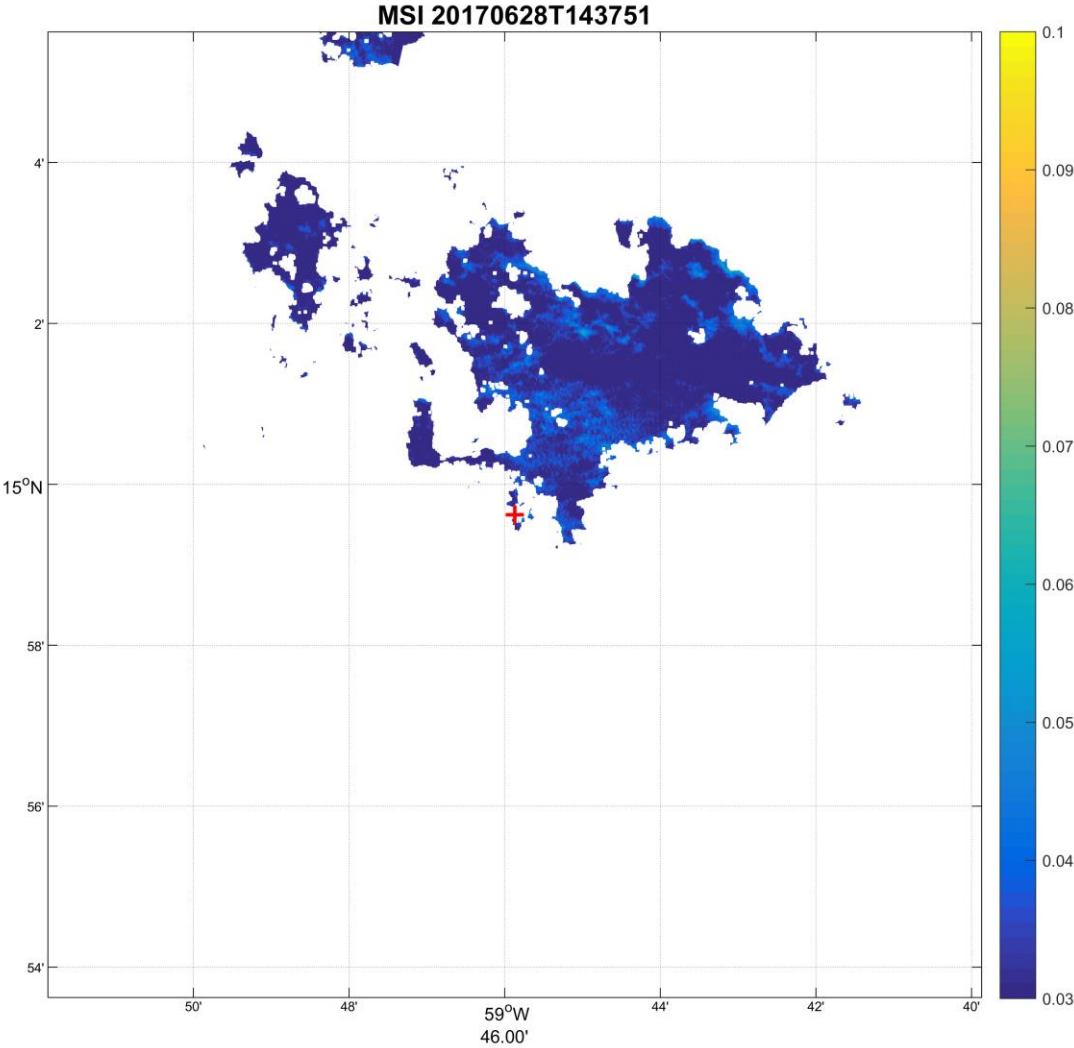

*In situ - Type 3*

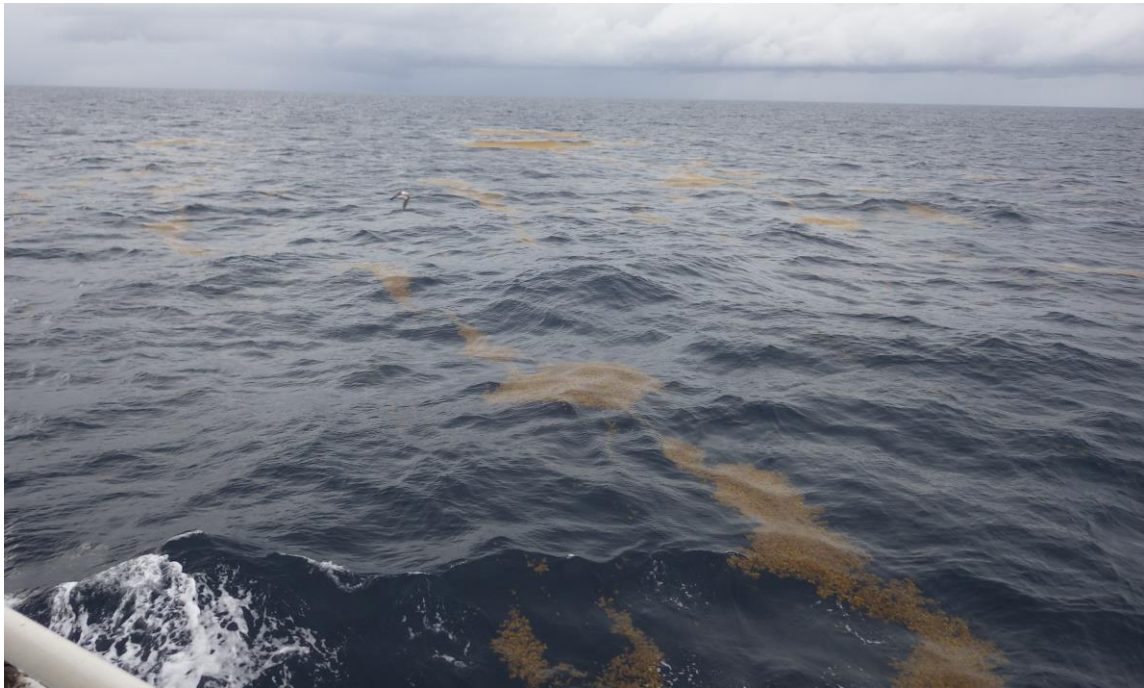

*MSI (10m) - 3 days*

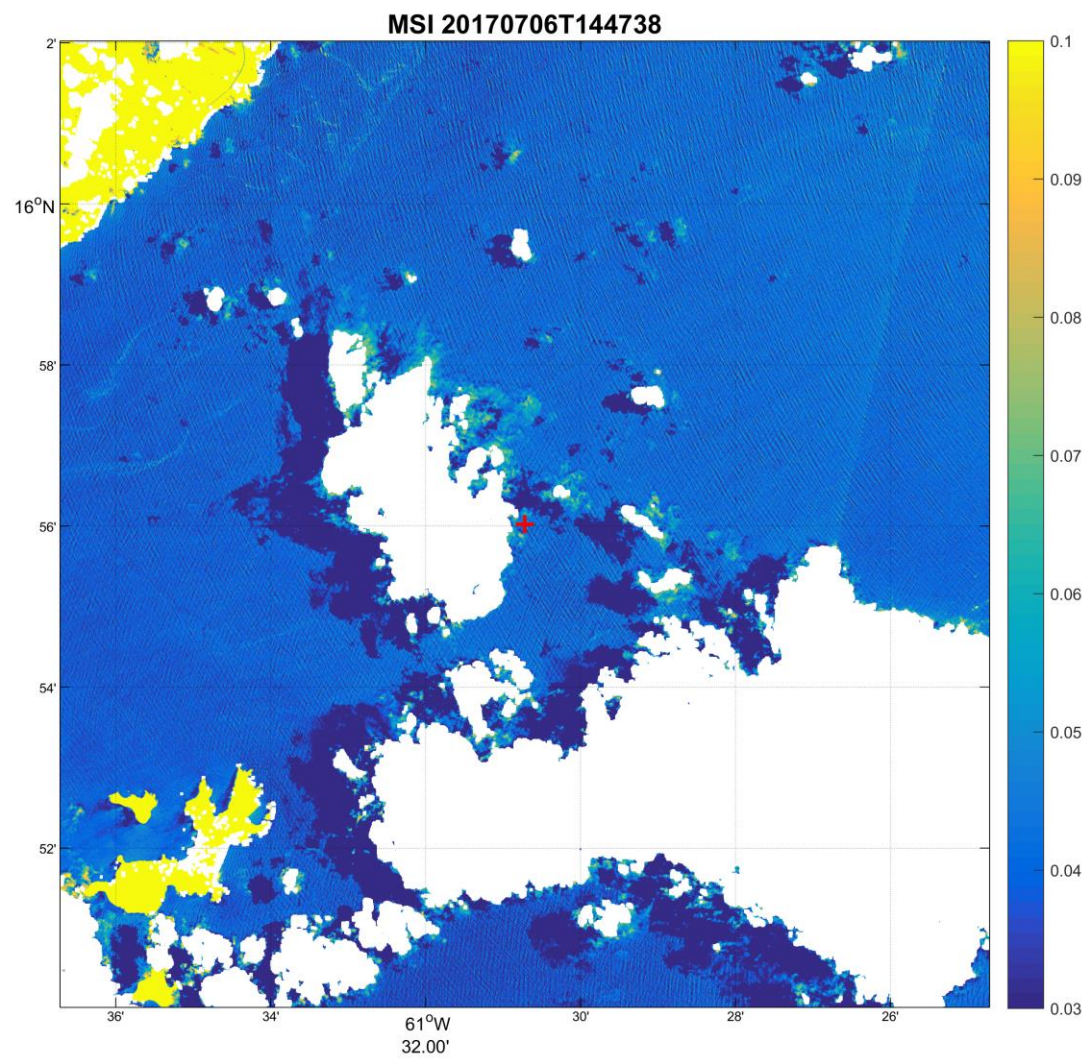

*In situ - Type 5*

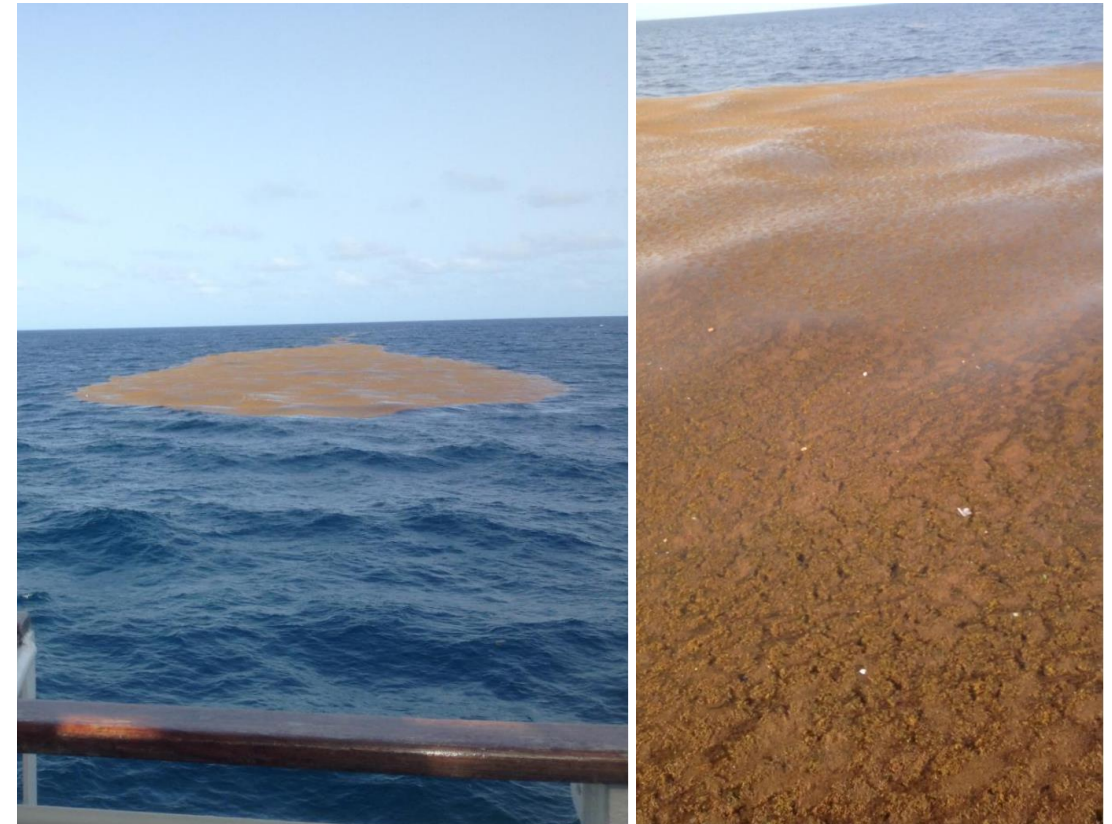

*MSI (10m) - 2 days*

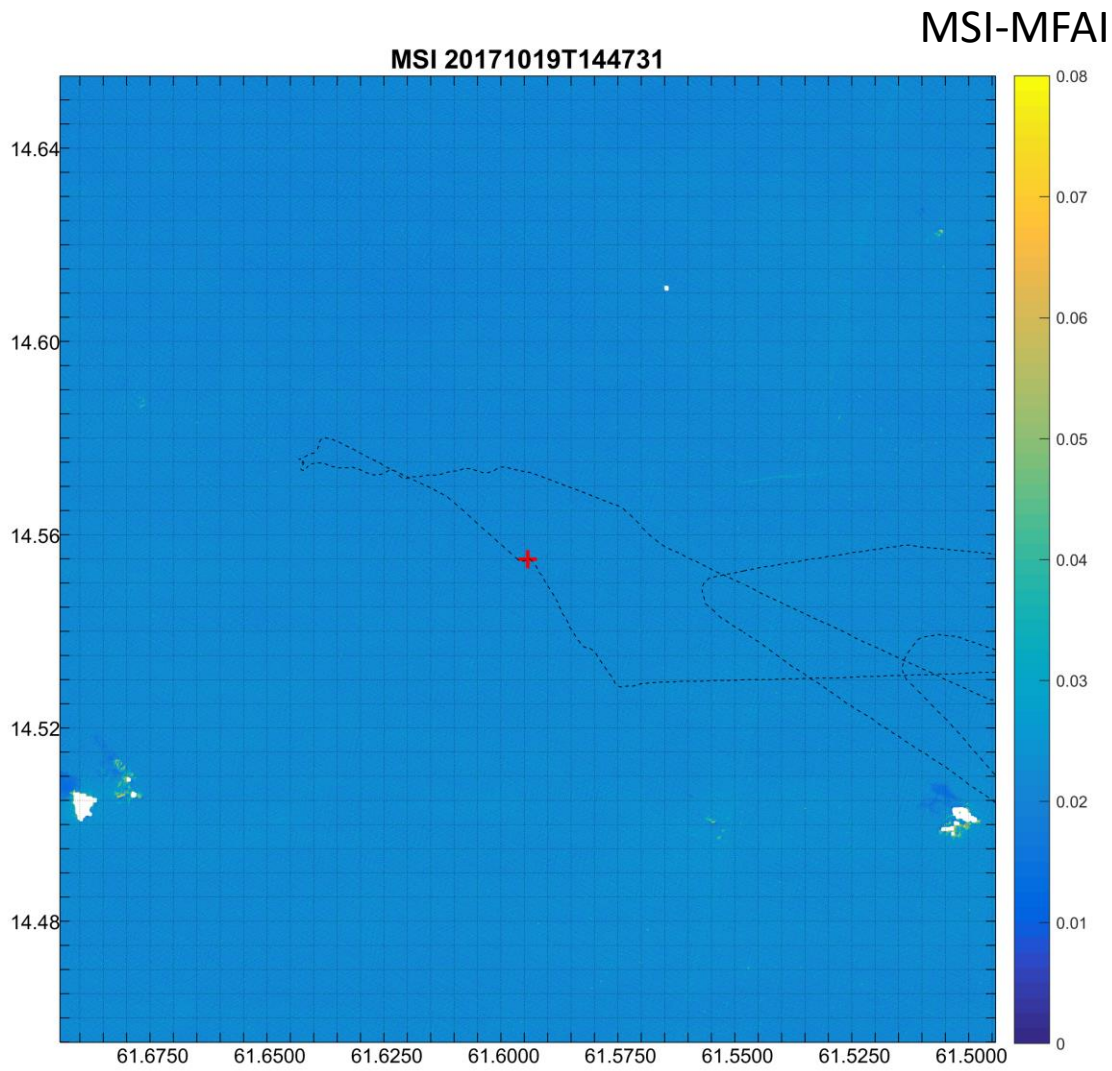

*In situ - Type 3*

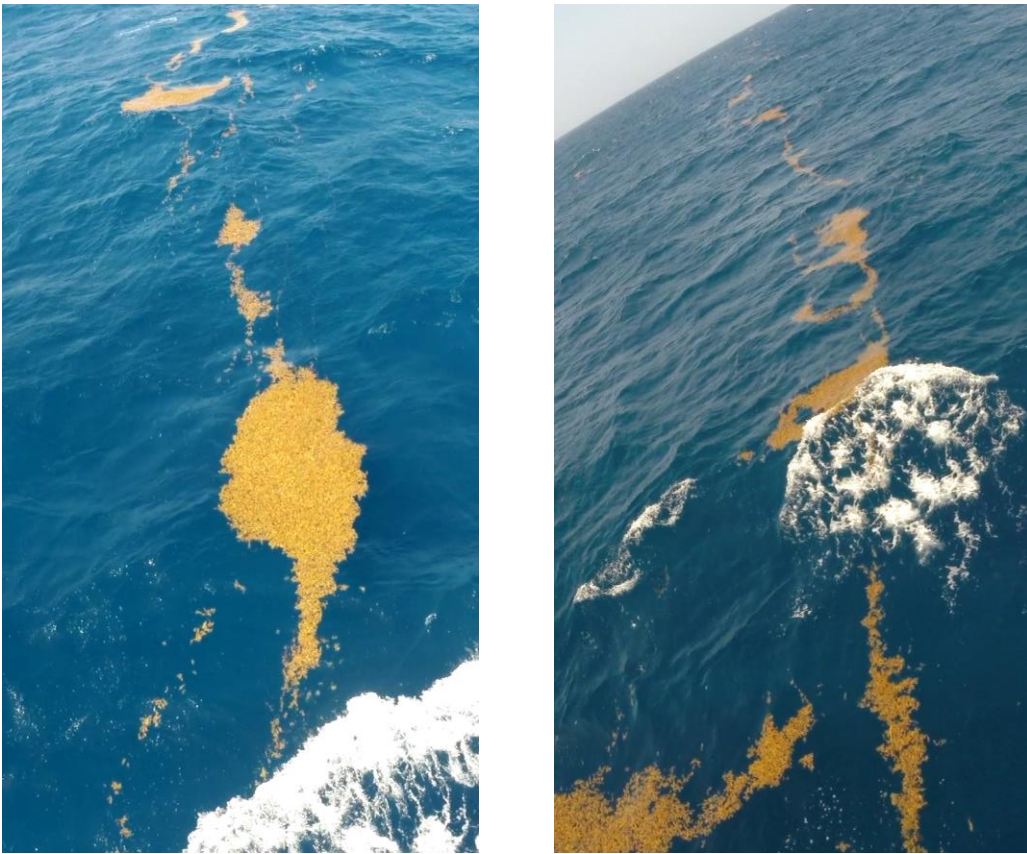

Supplement: S3 Fig — Matches are made within a +/- 3 days interval and in an area of 0.1° x 0.1° around the station. For the three stations, the following information are reported (see Tables 2 and 3): cruise name, date, time (UTC), Latitude (°N), Longitude (°W), Wind Speed (WS) and Wind Direction (WD) and Sea State (SS). The date, time and name of MSI observations are reported. (PDF) [file pone.0222584.s003.pdf]

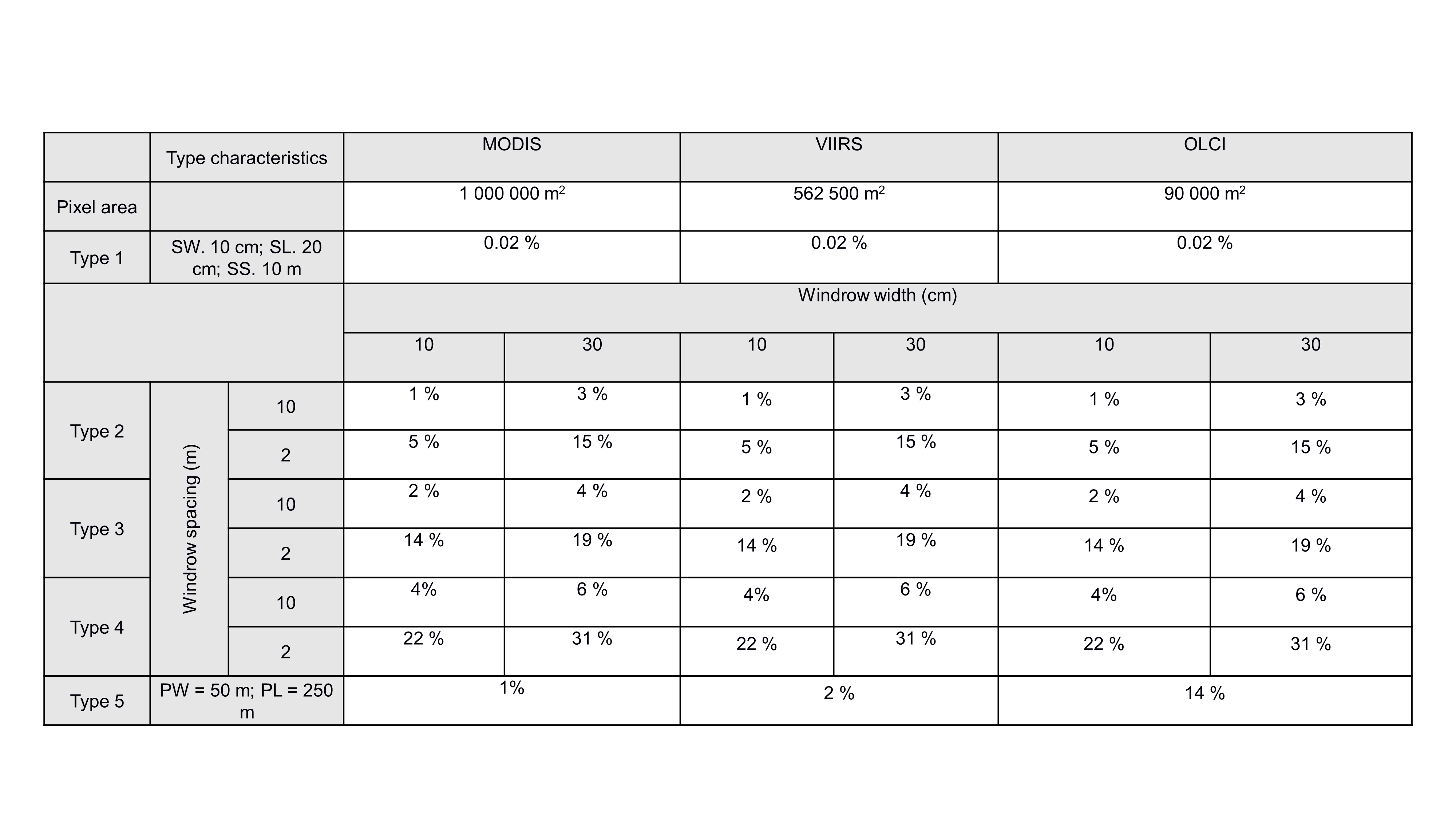

Supplement: S1 Table — For these estimations, Type 1 is characterized by one Sargassum thalli of width SW and length SL each SS meters. Types 2, 3 and 4 are characterized by Sargassum windrows with a width ranging between 10 and 30 cm and windrow spacing varying between 10 and 2 m (the 2 m spacing is used to estimate the maximum fractional coverage limit reached by each type, as this spacing was never encountered over large areas during the two cruises). For Types 3 and 4, we considered patches of 1 mdiameter each 10 m for Type 3 and 4 m diameter each 50 m for Type 4. For Type 5, we considered only one big patch of a size similar to the Type 5 raft encountered during the Transatlantic cruise (station Y07b) (Patch Width (PW) = 50m and Patch Length (PL) = 250m). (TIF) [file pone.0222584.s004.tif]
