# Supplementary material for: From In Situ to satellite observations of pelagic Sargassum distribution and aggregation in the Tropical North Atlantic Ocean
Source: PLoS One. 2019 Sep 17;14(9):e0222584. doi: 10.1371/journal.pone.0222584 (PMC6748567; doi:10.1371/journal.pone.0222584)

S2 Fig – 1. WEST ATLANTIC - S1 - 2017-06-21 15.41 UTC - 05°03.390' N 52°02.580' W - WS = 4.1 $m.s^{-1}$ WD = 264° SS = Slight

MODIS-A (1km)

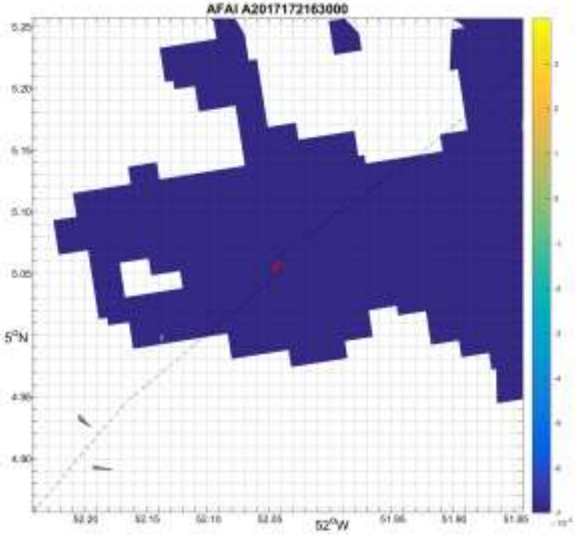

MODIS-T (1km)

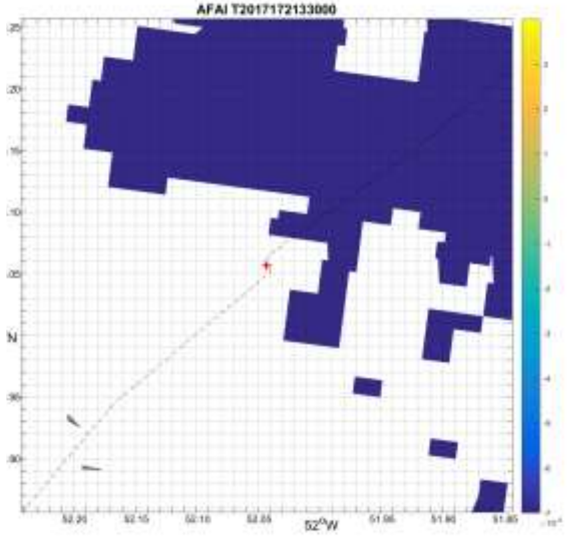

VIIRS (750 m)

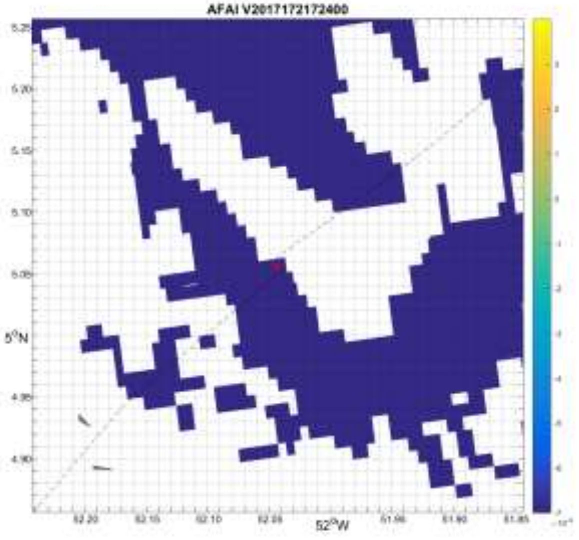

OLCI (300m)

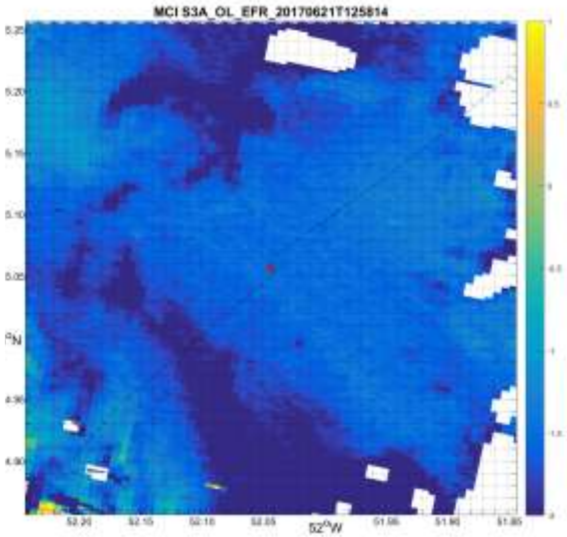

In situ - absence

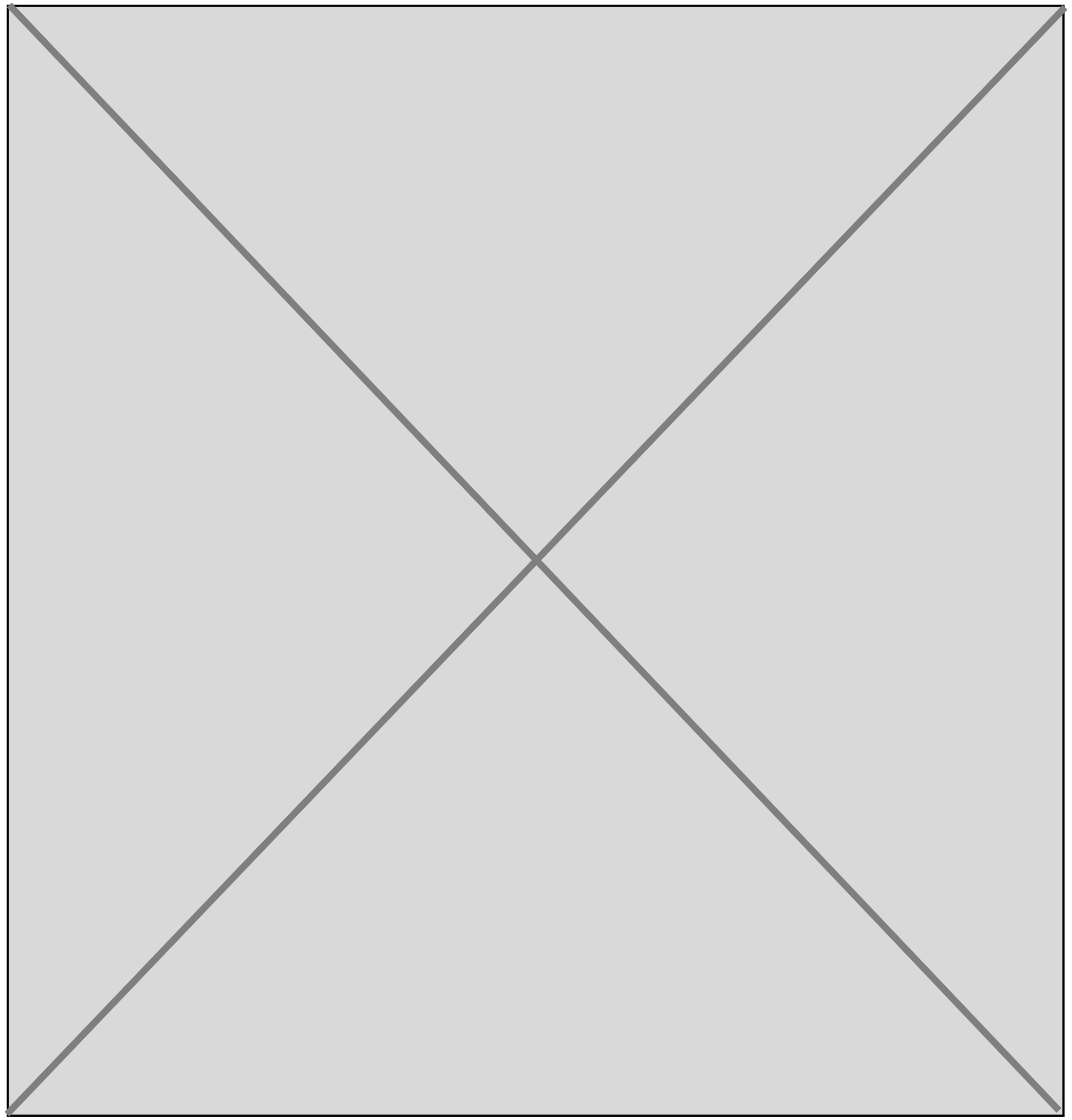

S2 Fig – 2. WEST ATLANTIC - S2 - 2017-06-22 20.18 UTC - 07°50.950' N 48°59.550' W - WS = 6.6 $m.s^{-1}$ WD = 245° SS = Moderate

MODIS-A (1km)

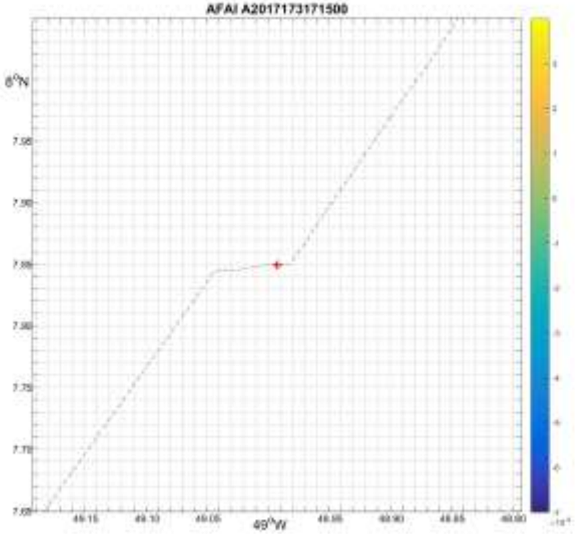

MODIS-T (1km)

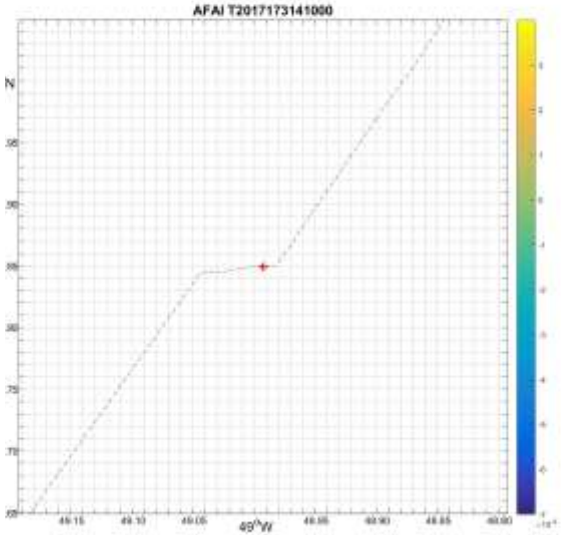

VIIRS (750 m)

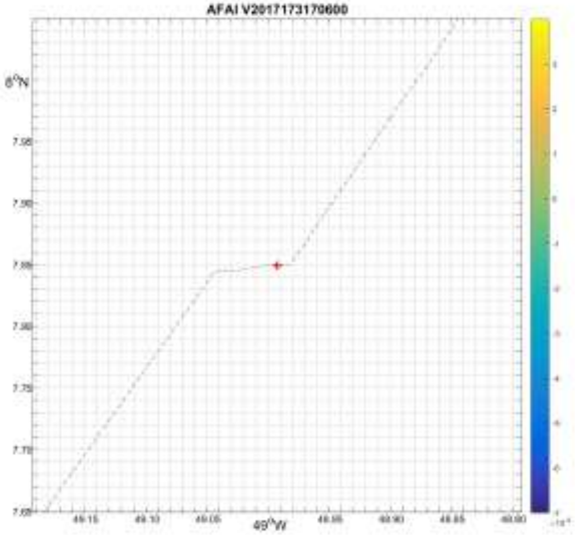

OLCI (300m)

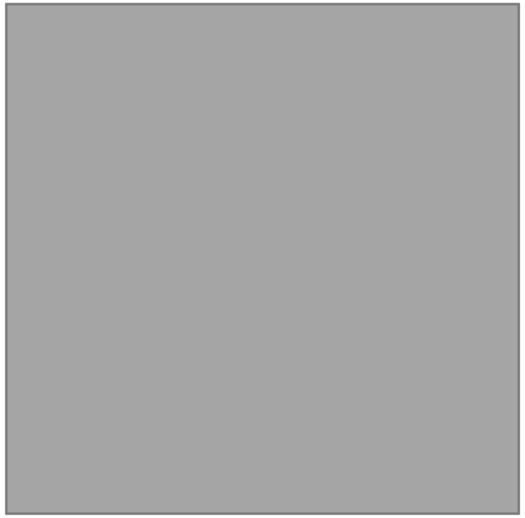

In situ - Type 3

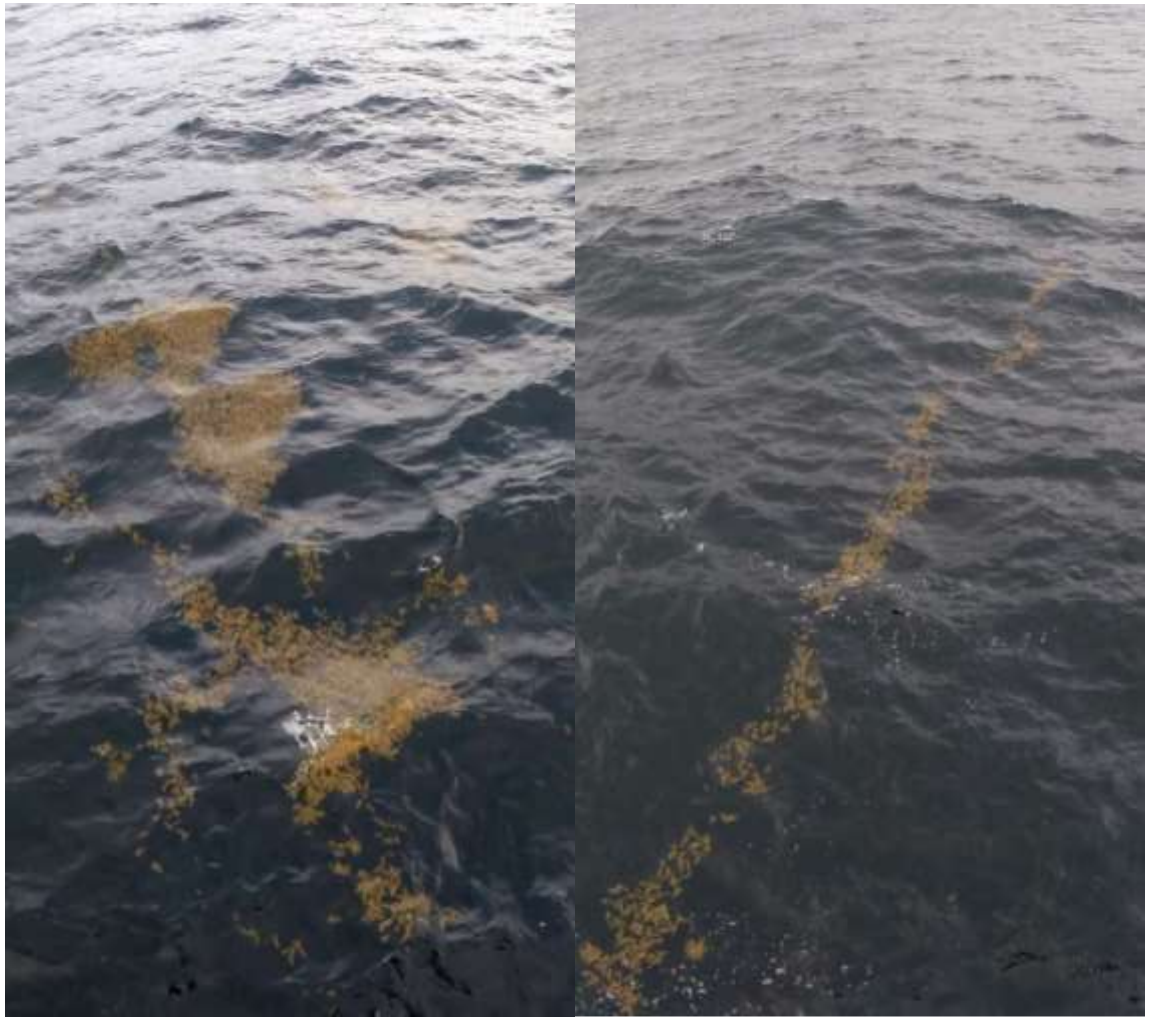

S2 Fig – 3. WEST ATLANTIC - S3 - 2017-06-23 10.35 UTC - 08°33.710' N 48°29.940' W - WS = 5.5 m.s^{-1} WD = 254° SS = Slight

MODIS-A (1km)

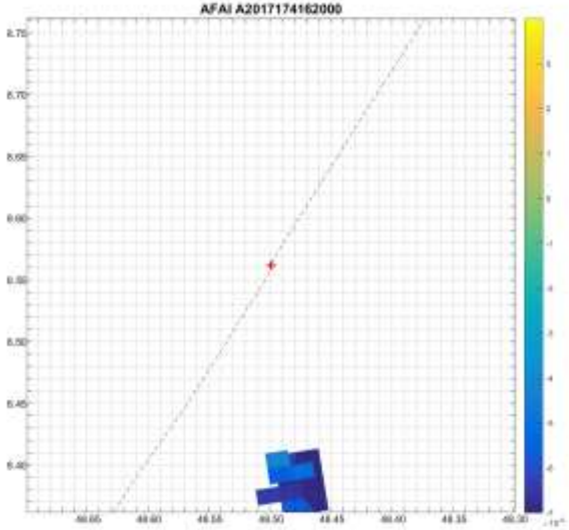

MODIS-T (1km)

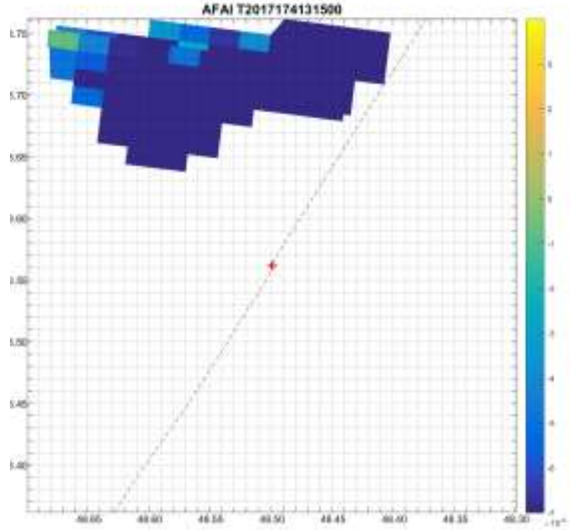

VIIRS (750 m)

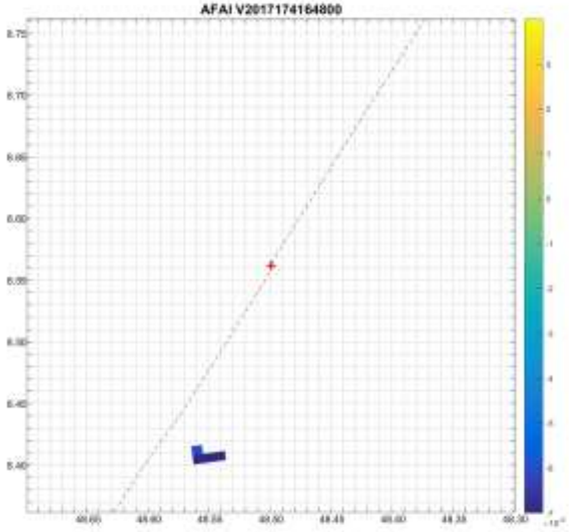

OLCI (300m) + 1 jour

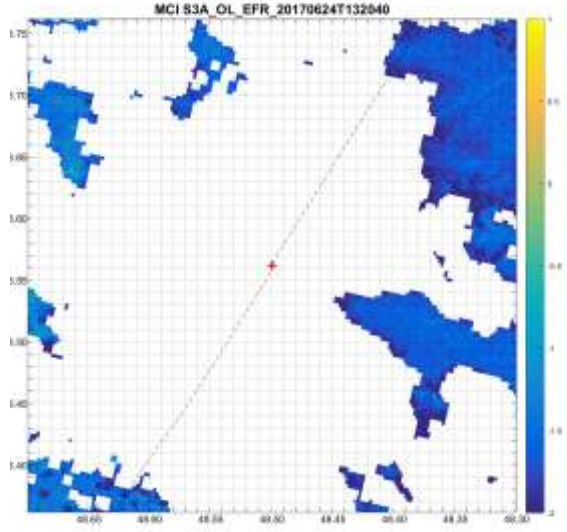

In situ - Type 3

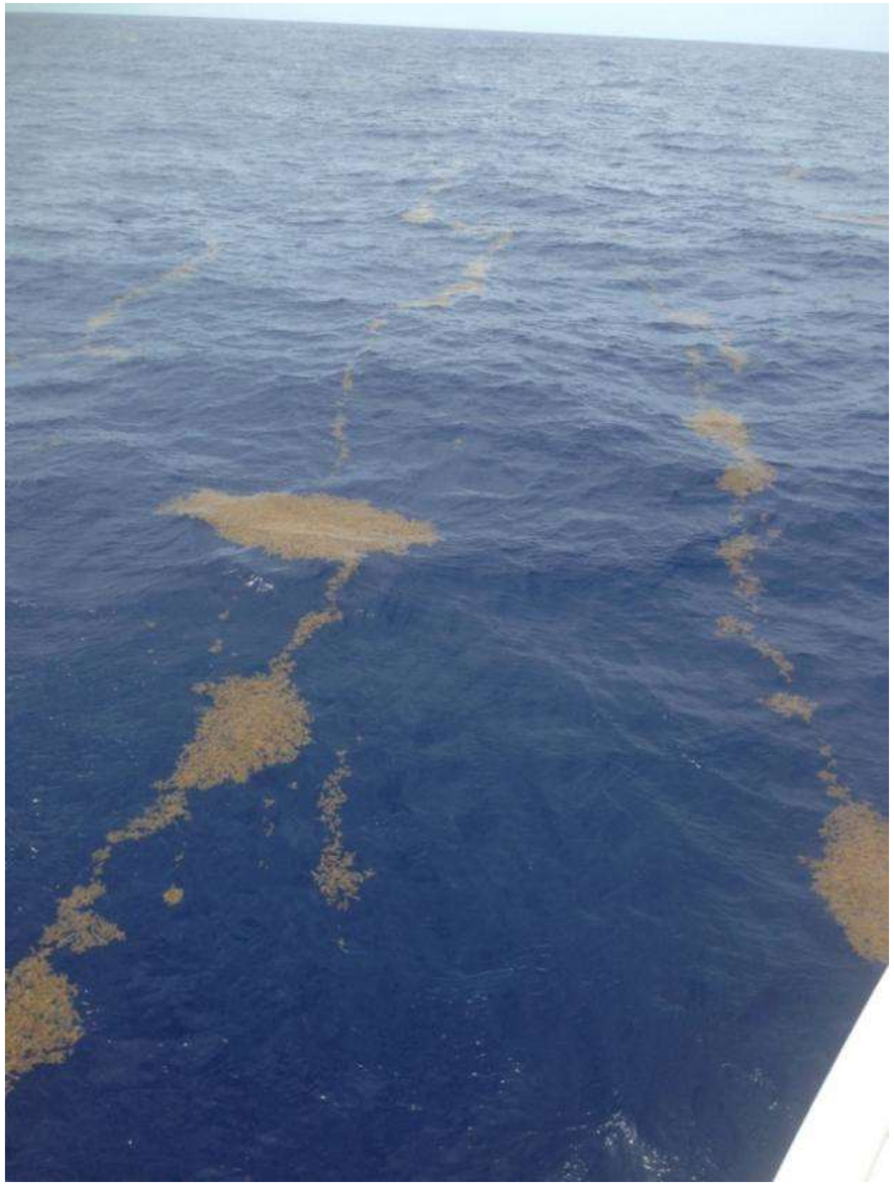

S2 Fig – 4. WEST ATLANTIC - S4 - 2017-06-23 20.04 UTC - 08°51.950'N 49°08.380'W - WS = 6.1 m.s^{-1} WD = 252° SS = Slight

MODIS-A (1km)

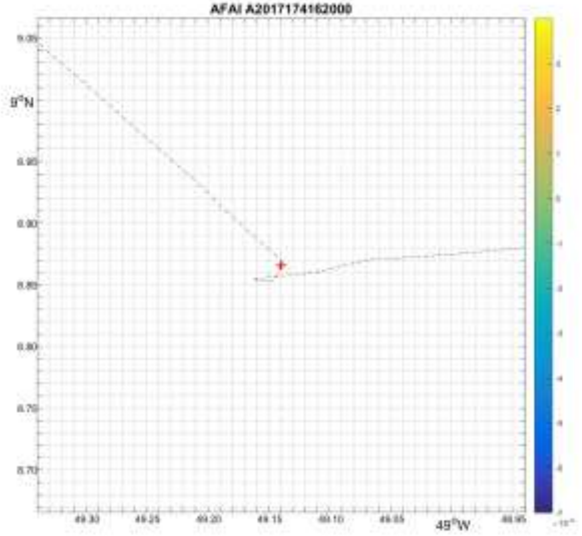

MODIS-T (1km)

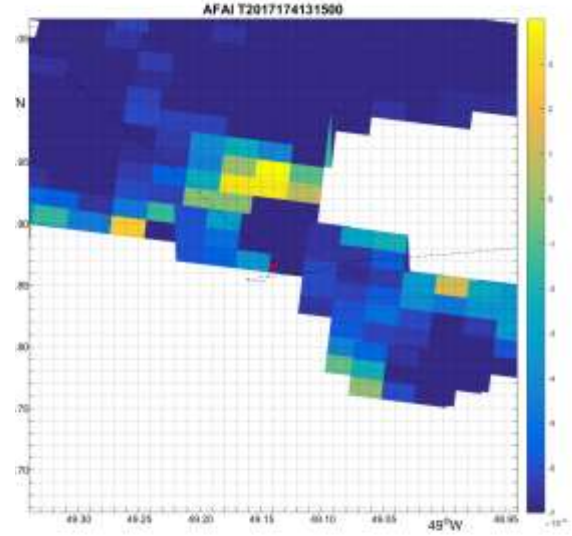

VIIRS (750 m)

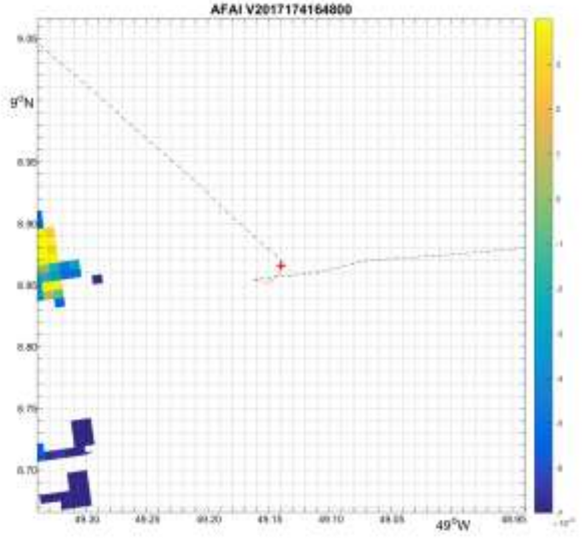

OLCI (300m) + 1 day

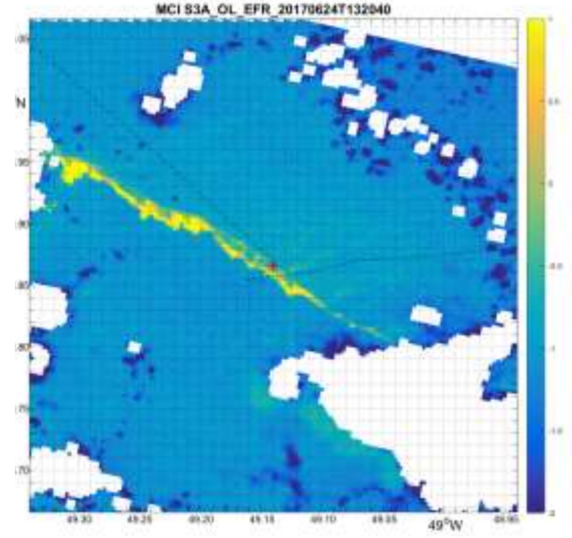

In situ - Type 3

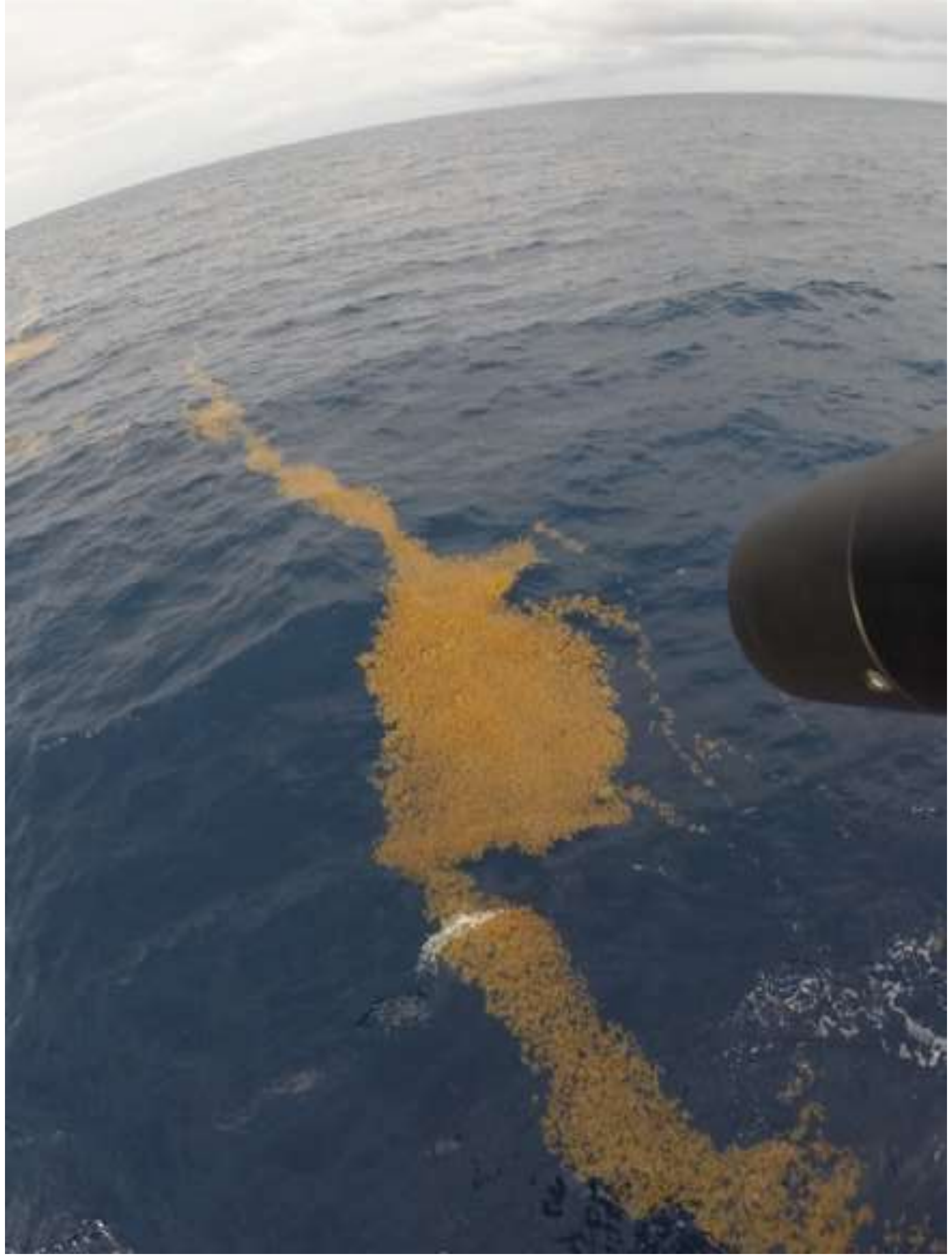

S2 Fig – 5. WEST ATLANTIC - S5 - 2017-06-24 12.24 UTC - 10°32.630'N 51°02.530'W - WS = 8.4 m.s^{-1} WD = 258° SS = Moderate

MODIS-A (1km)

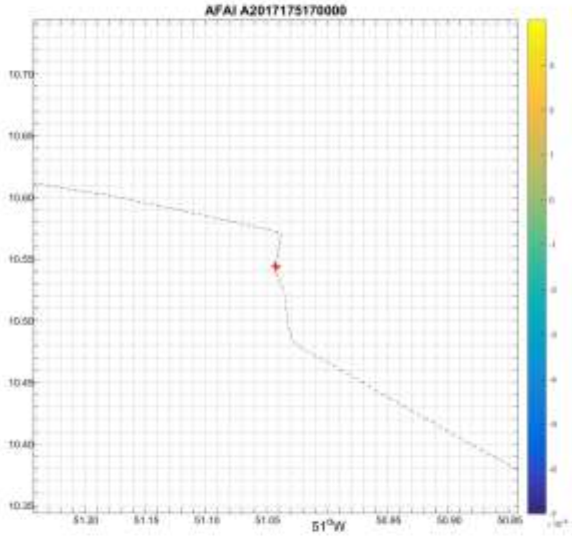

MODIS-T (1km)

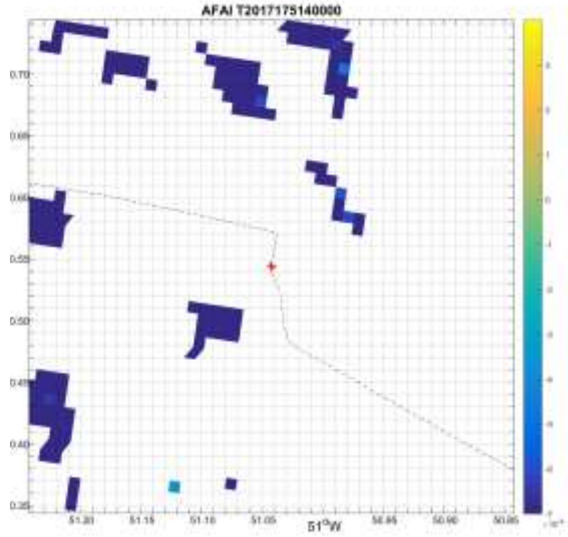

VIIRS (750 m)

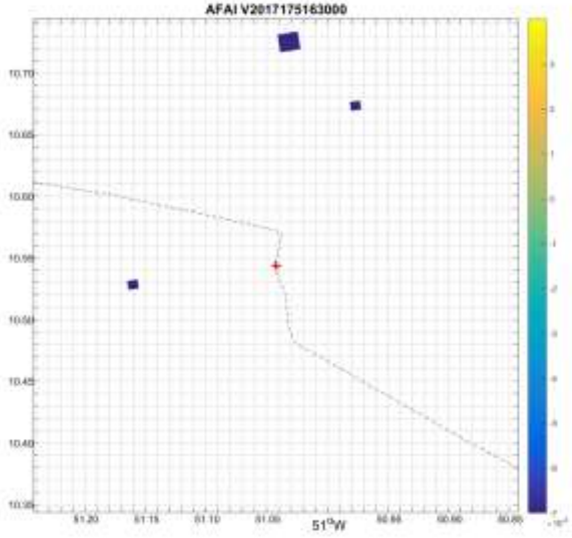

OLCI (300m)

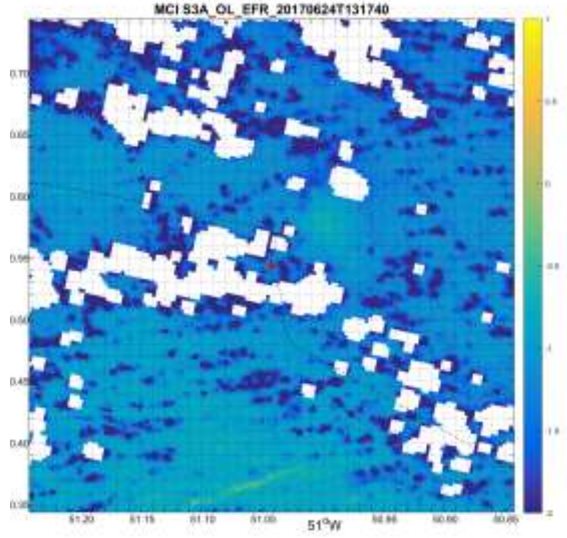

In situ - Type 2

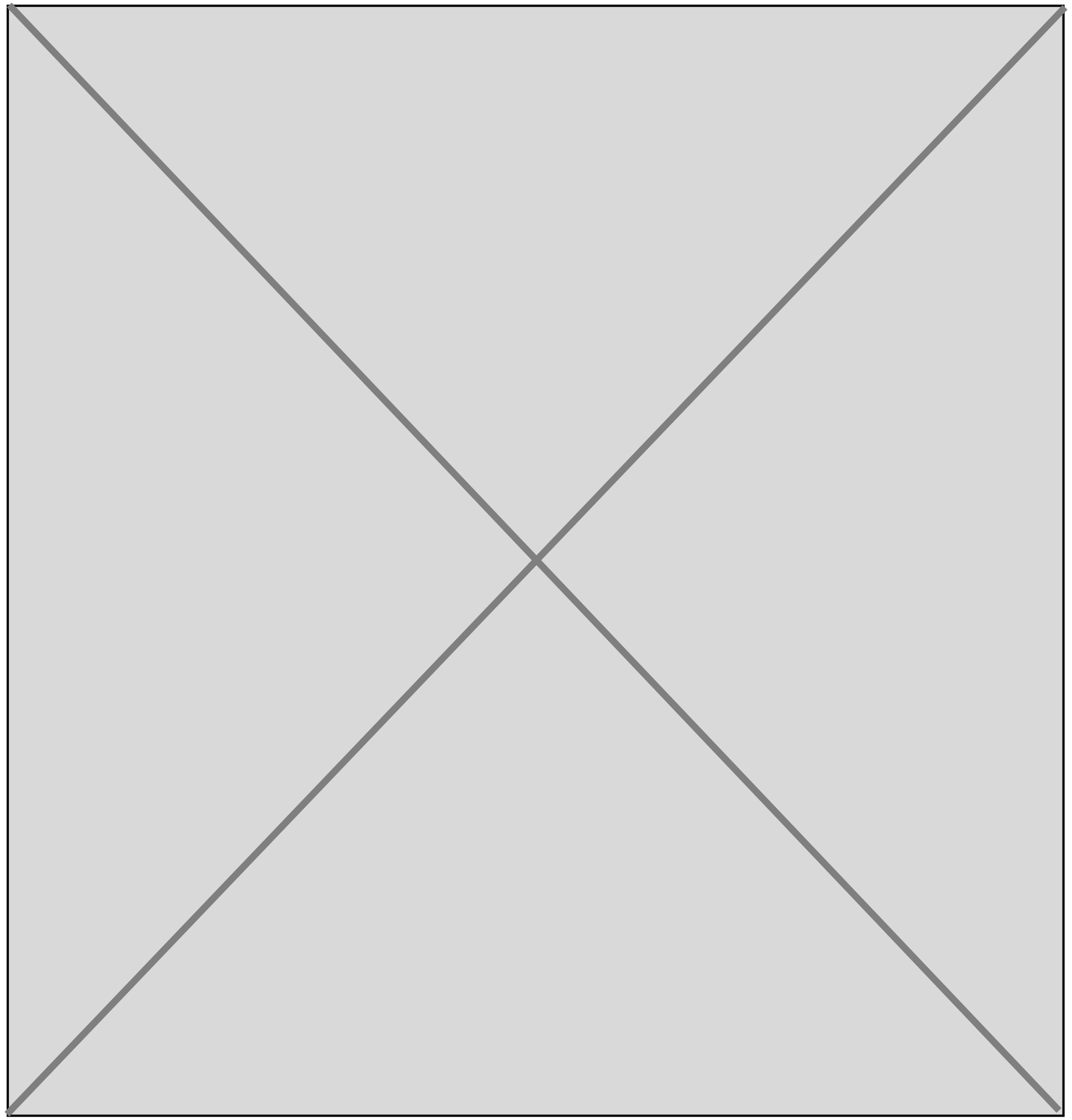

S2 Fig – 6. WEST ATLANTIC - S6 - 2017-06-24 19.22 UTC - 10°43.540'N 51°46.030'W - WS = 9.0 m.s^{-1} WD = 260° SS = Moderate

MODIS-A (1km)

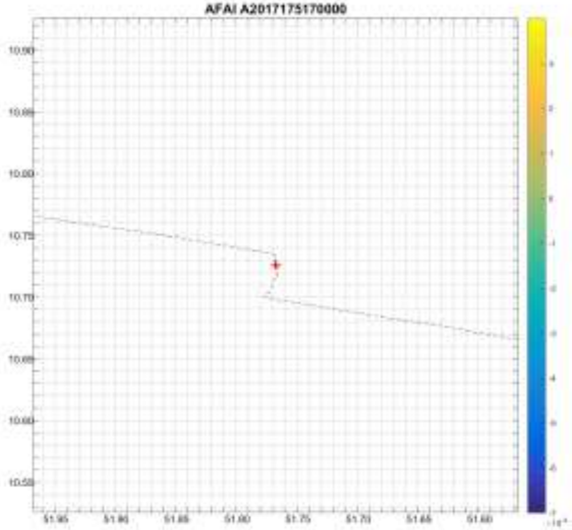

MODIS-T (1km)

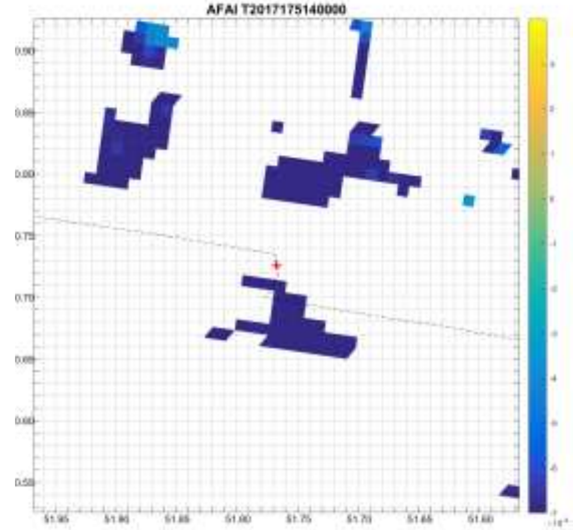

VIIRS (750 m)

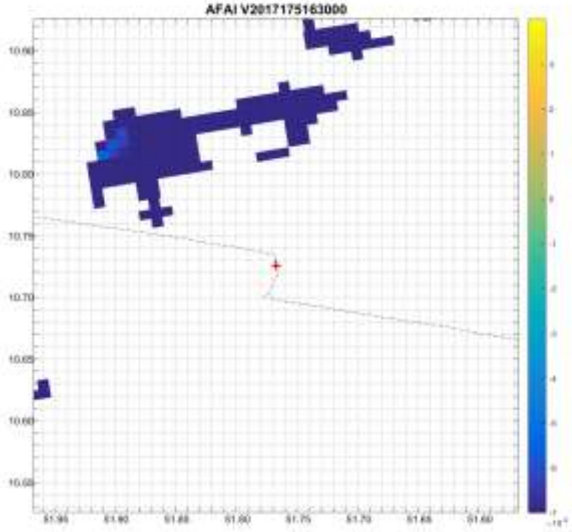

OLCI (300m)

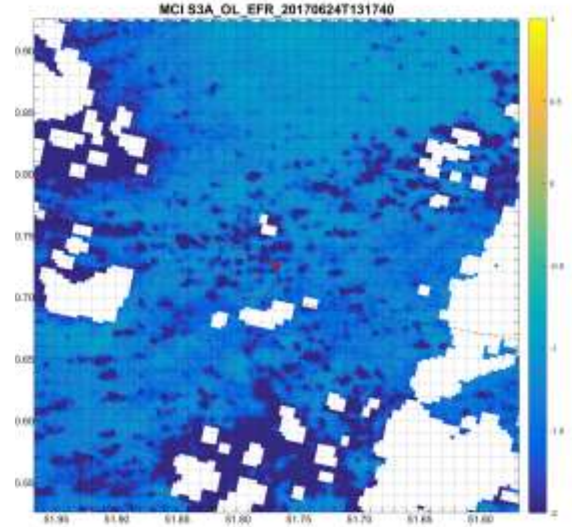

In situ - Type 2

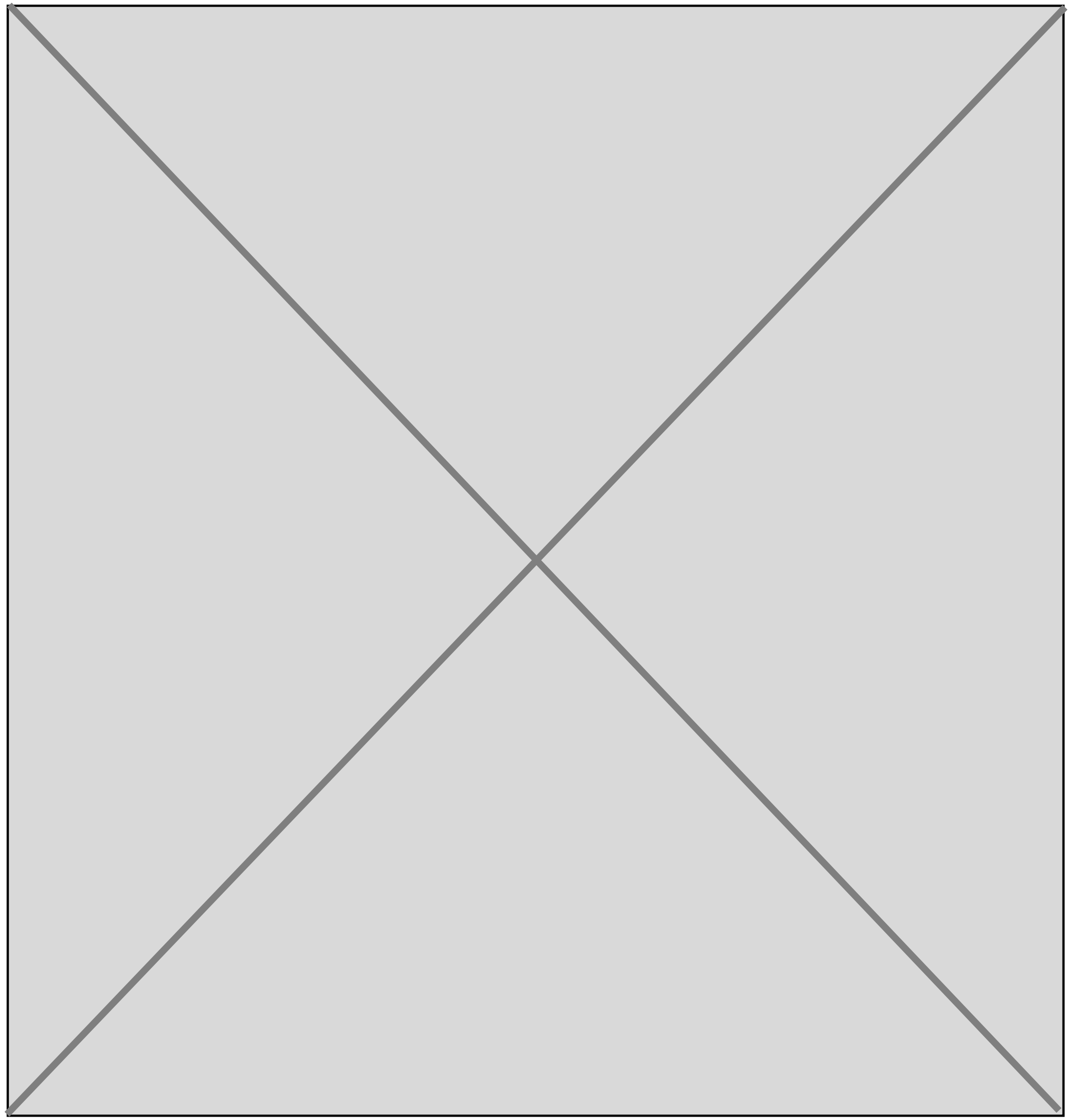

S2 Fig – 7. WEST ATLANTIC - S7 - 2017-06-25 12.08 UTC - 11°13.690'N 53°49.410'W - WS = 8.8 m.s^{-1} WD = 247° SS = Moderate

MODIS-A (1km)

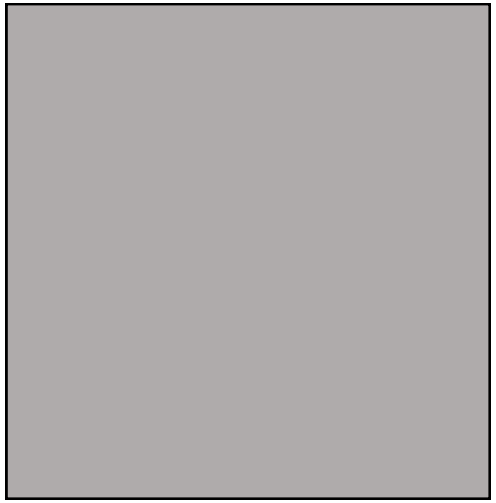

MODIS-T (1km)

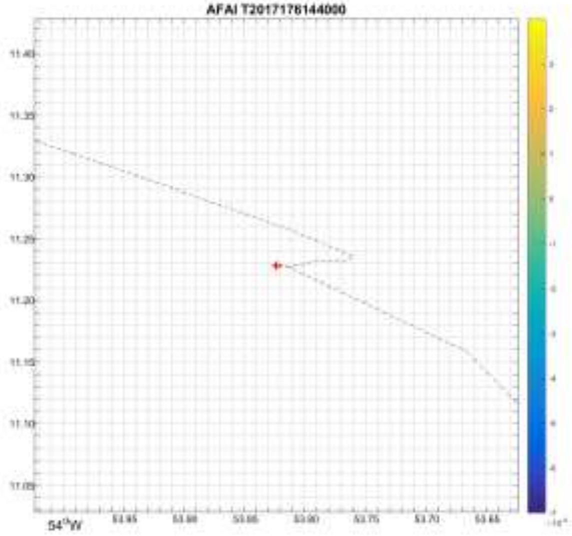

In situ - Type 3

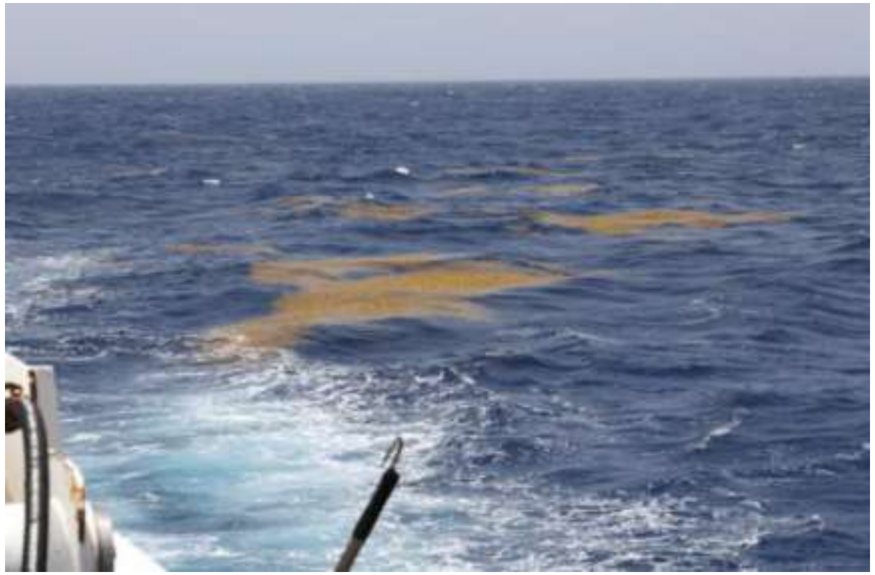

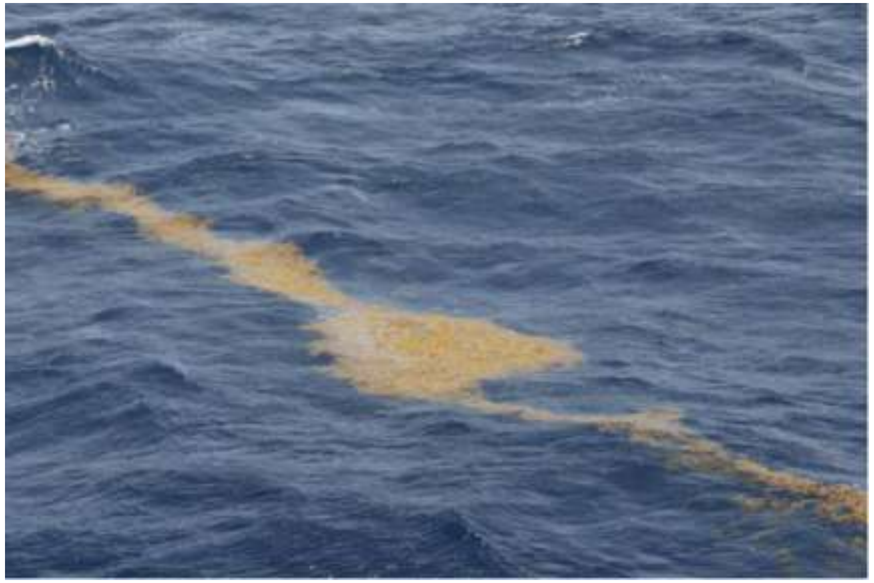

VIIRS (750 m)

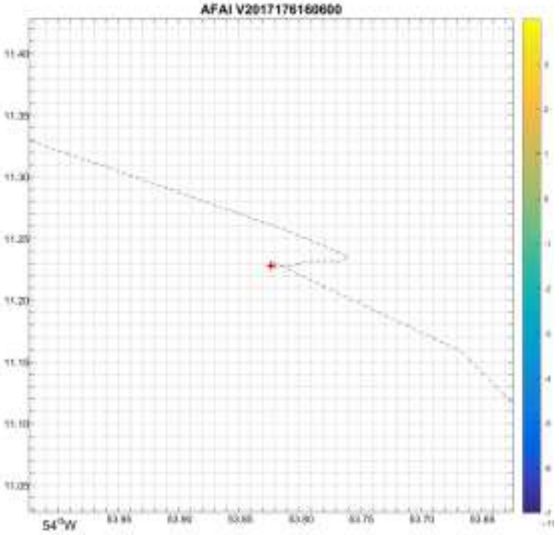

OLCI (300m) - 1 day

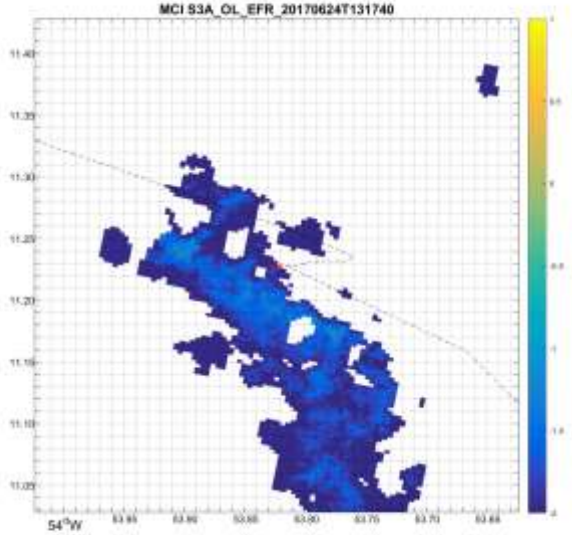

S2 Fig – 8. WEST ATLANTIC - S8 - 2017-06-25 19.59 UTC - 11°24.579'N 54°14.634'W - WS = 9.1 m.s^{-1} WD = 246° SS = Moderate

MODIS-A (1km)

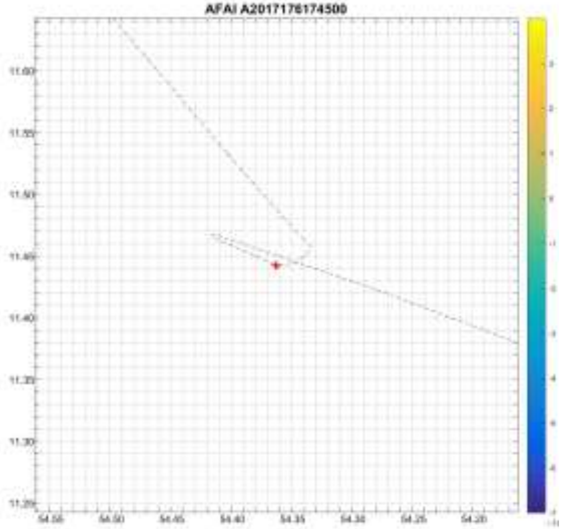

MODIS-T (1km)

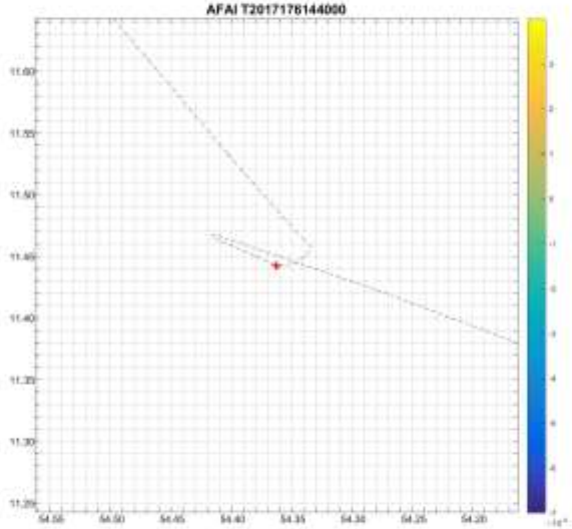

VIIRS (750 m)

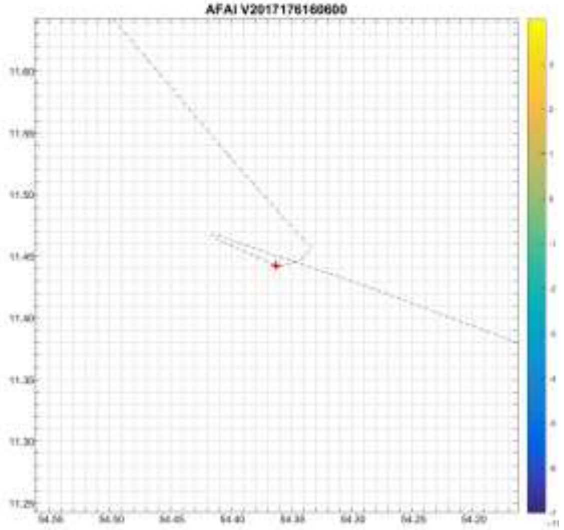

OLCI (300m) - 1 day

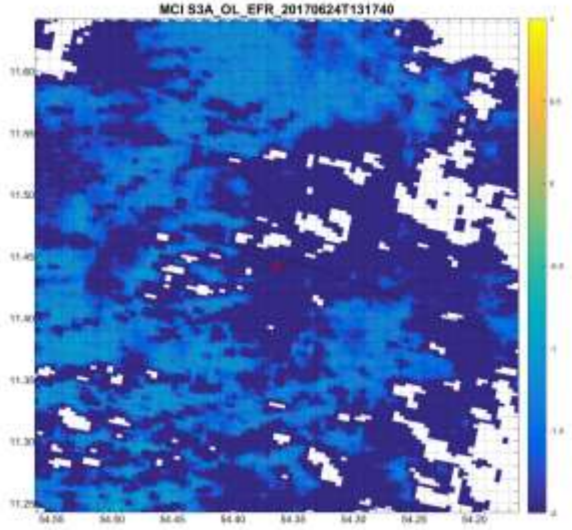

Affected by haze (diminish the MCI value)

In situ - Type 2

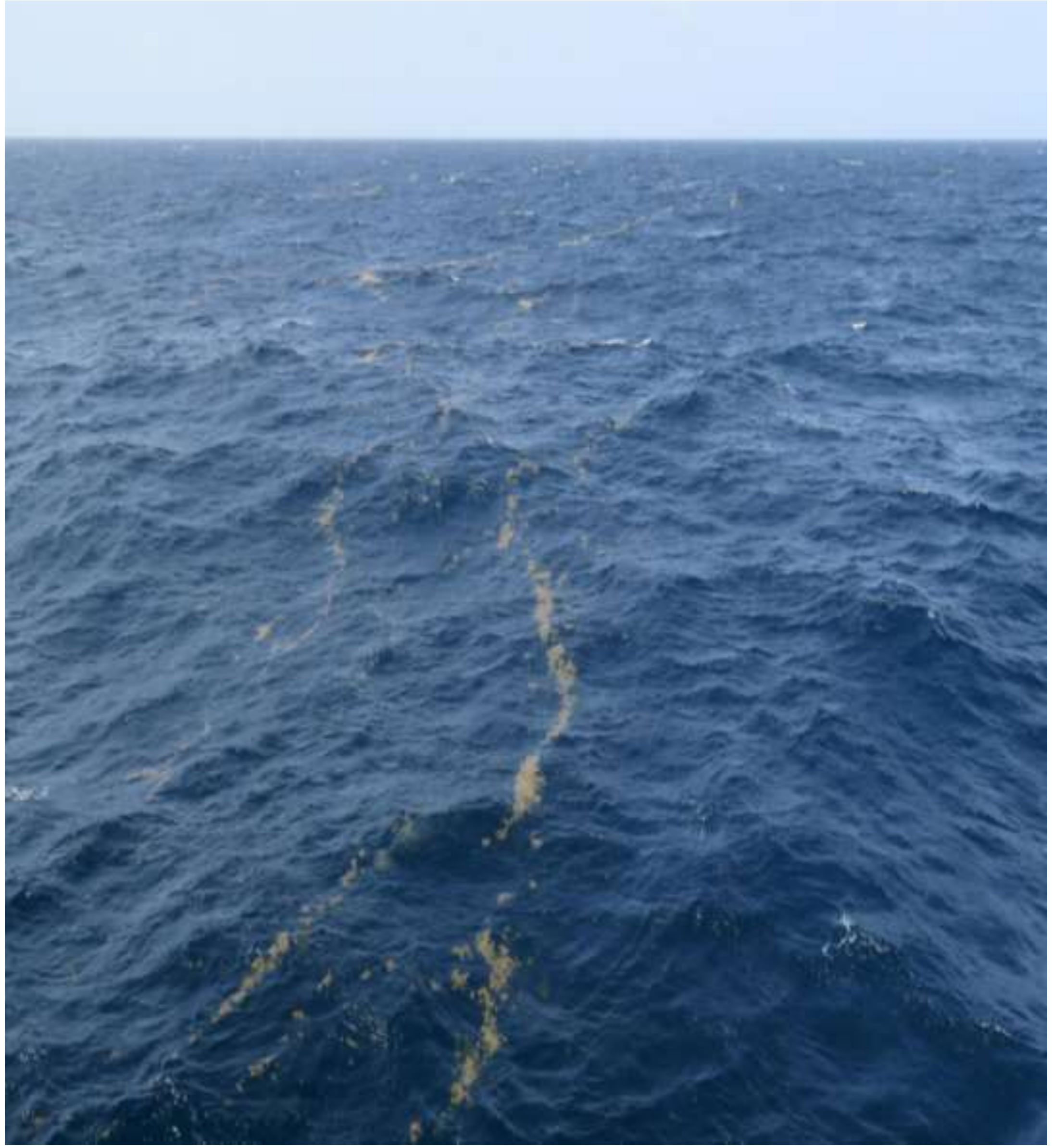

S2 Fig – 9. WEST ATLANTIC - S9 - 2017-06-26 12.50 UTC - 12°46.160'N 55°31.040'W - WS = 9.2 $m.s^{-1}$ WD = 241° SS = Moderate

MODIS-A (1km)

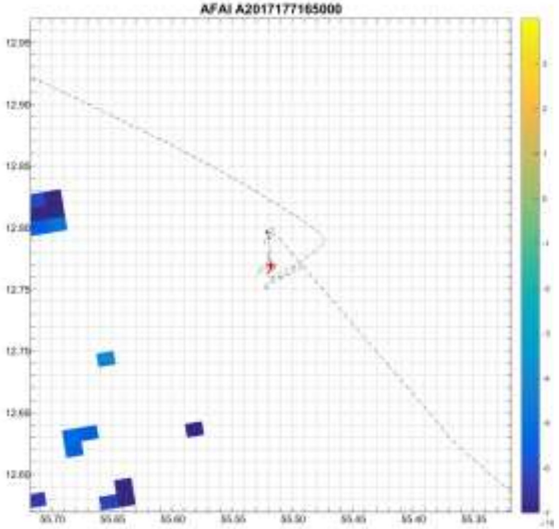

MODIS-T (1km)

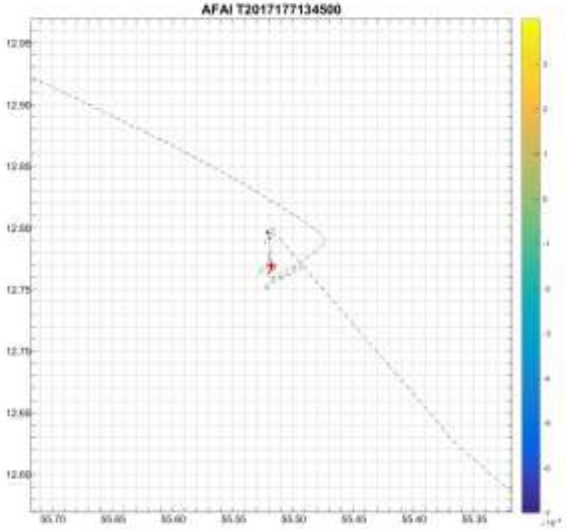

VIIRS (750 m)

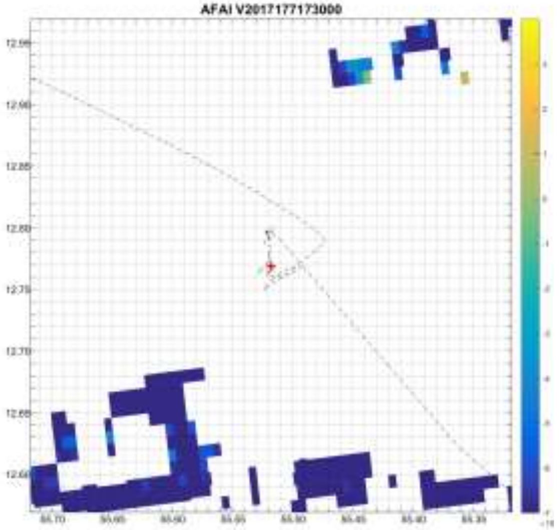

OLCI (300m) + 1 day

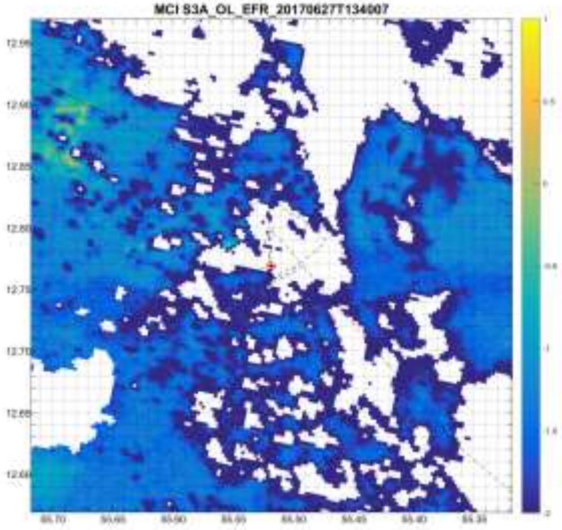

In situ - Type 5

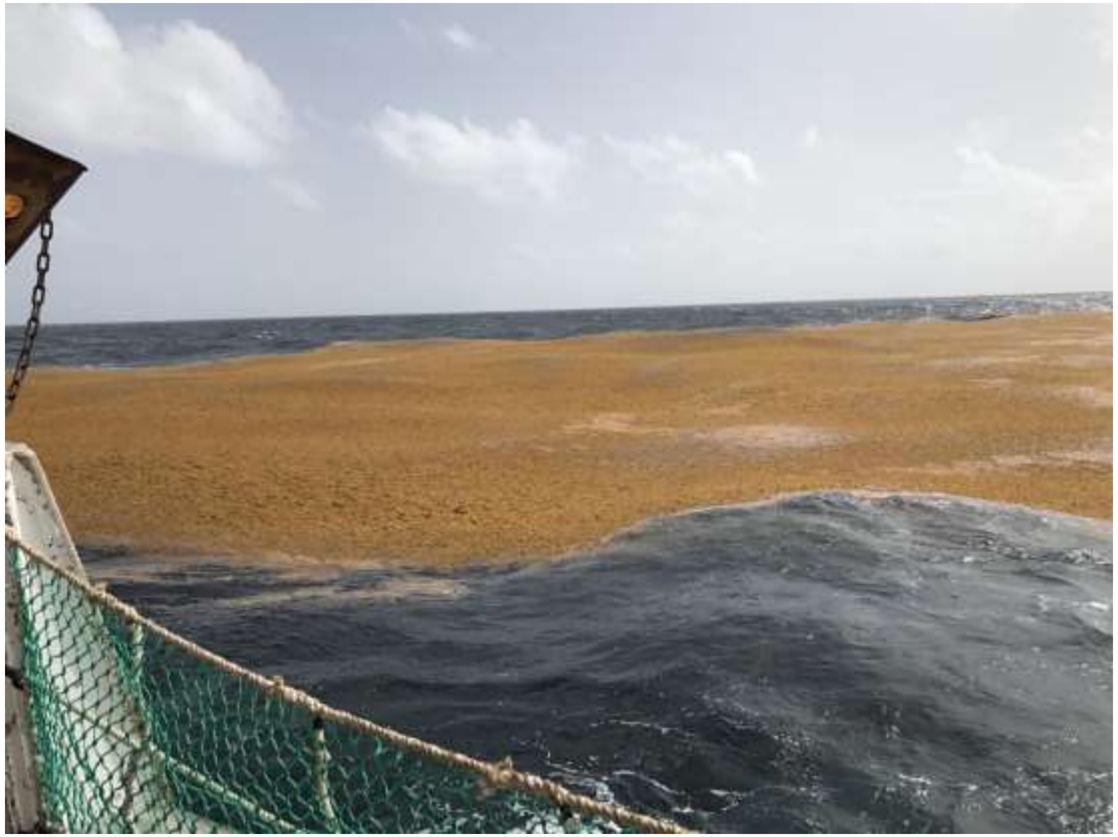

S2 Fig – 10. WEST ATLANTIC - S10 - 2017-06-27 19.06 UTC - 14°58.440'N 59°07.230'W - WS = 8.7 m.s^{-1} WD = 247° SS = Moderate

MODIS-A (1km)

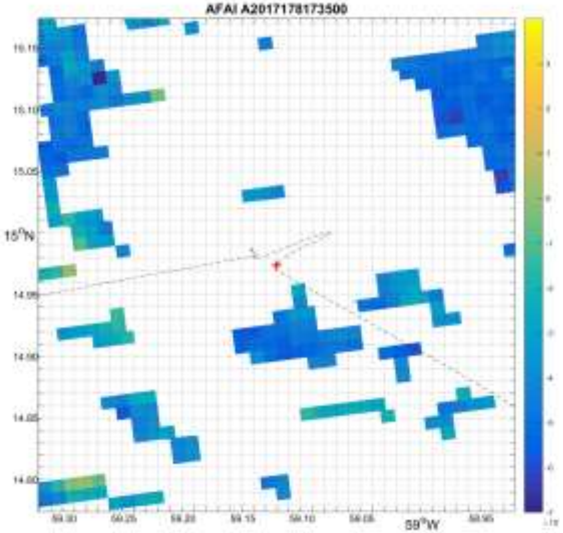

MODIS-T (1km)

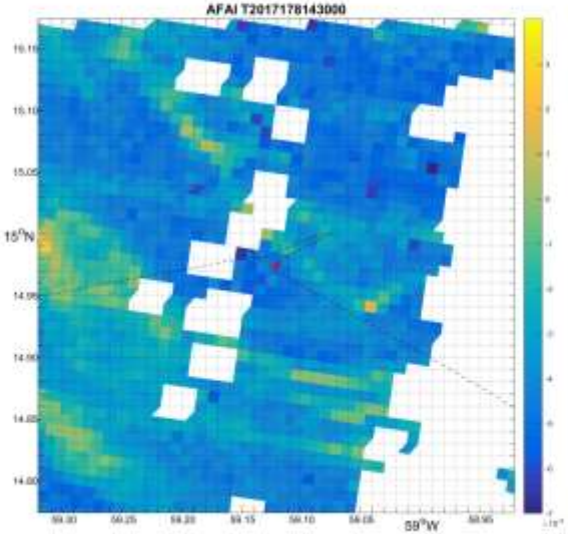

VIIRS (750 m)

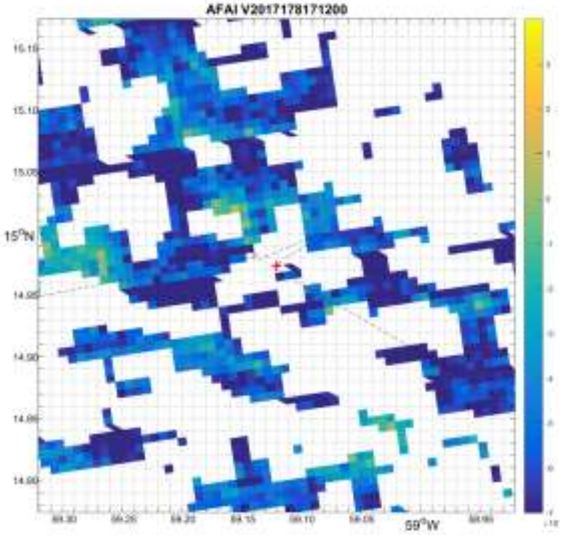

OLCI (300m)

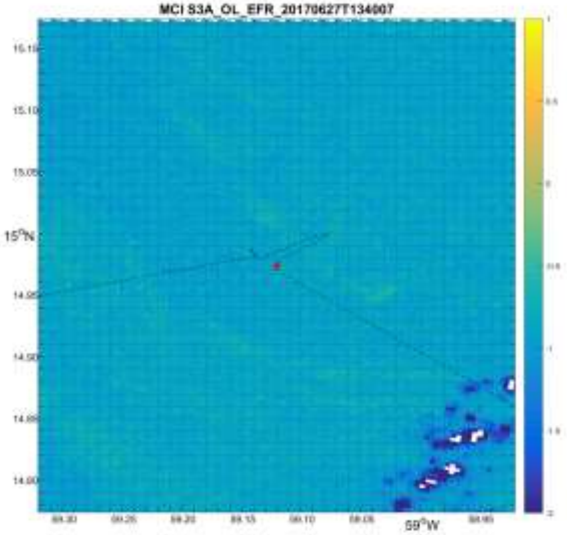

In situ - Type 3

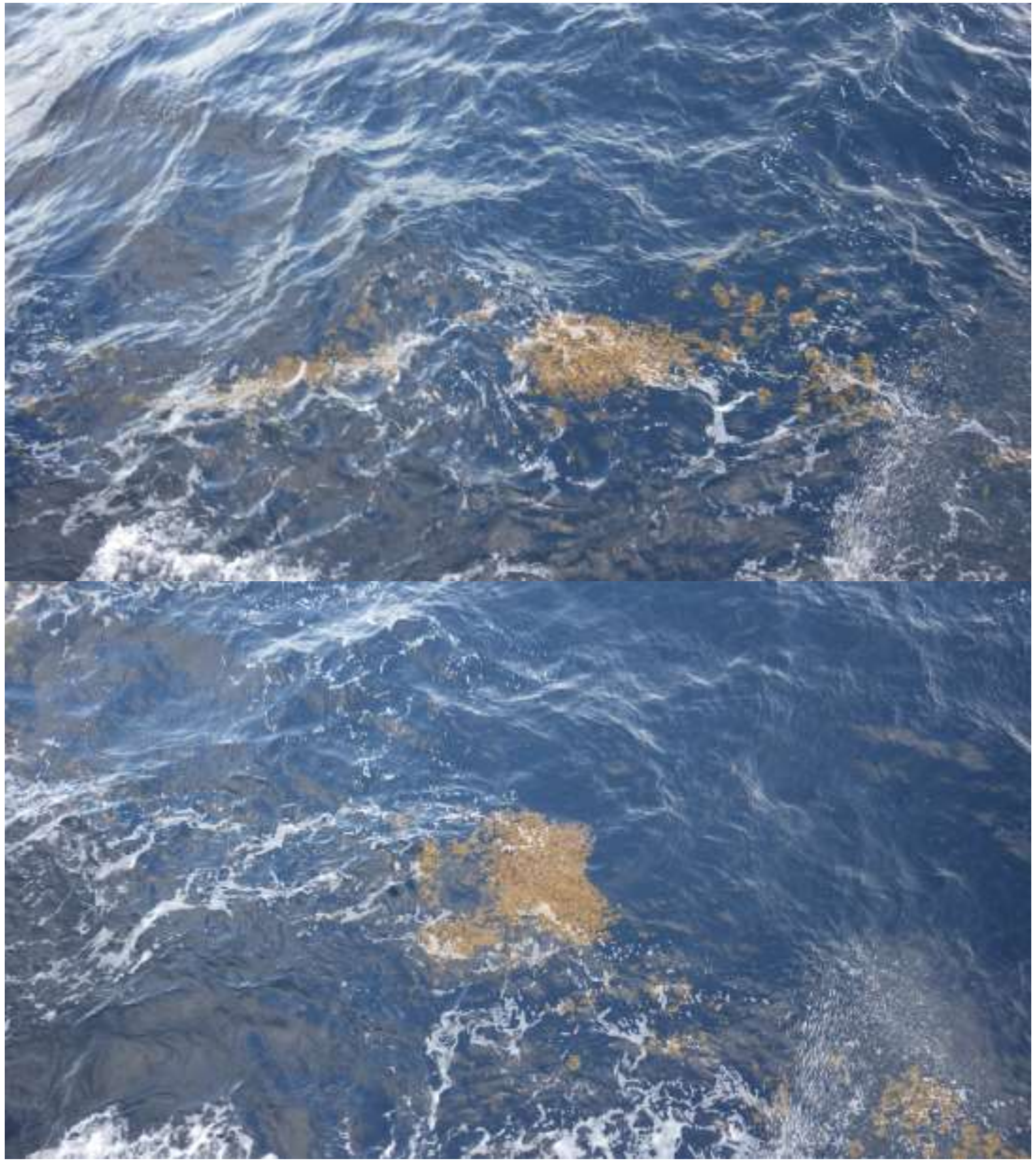

S2 Fig – 11. WEST ATLANTIC - S11 - 2017-06-28 11.57 UTC - 14°59.620'N 59°45.870'W - WS = 8.7 m.s^{-1} WD = 266° SS = Moderate

MODIS-A (1km)

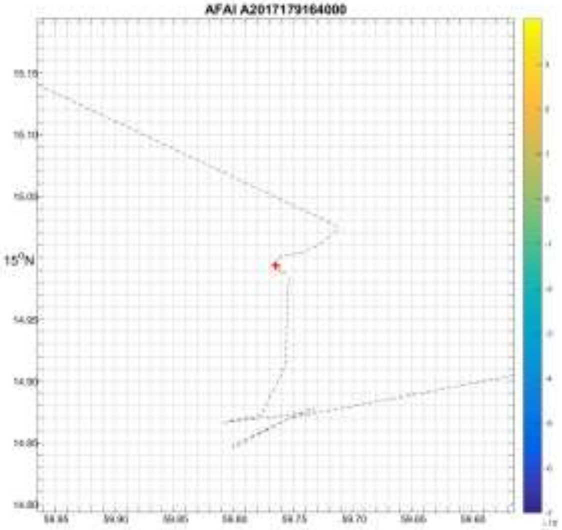

MODIS-T (1km)

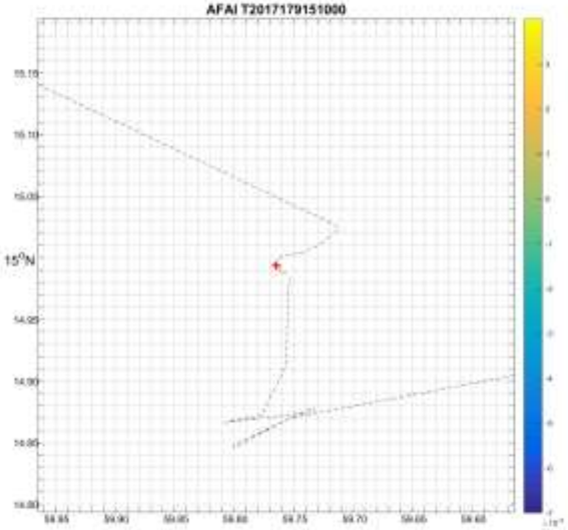

In situ - Type 3

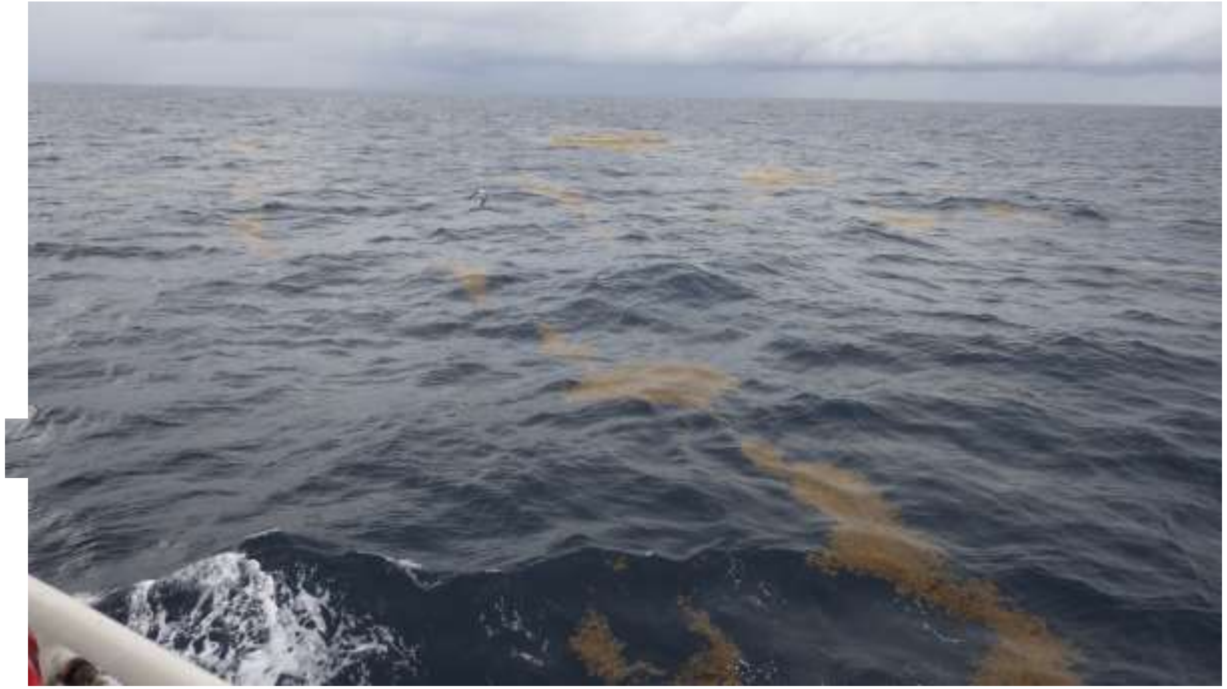

VIIRS (750 m)

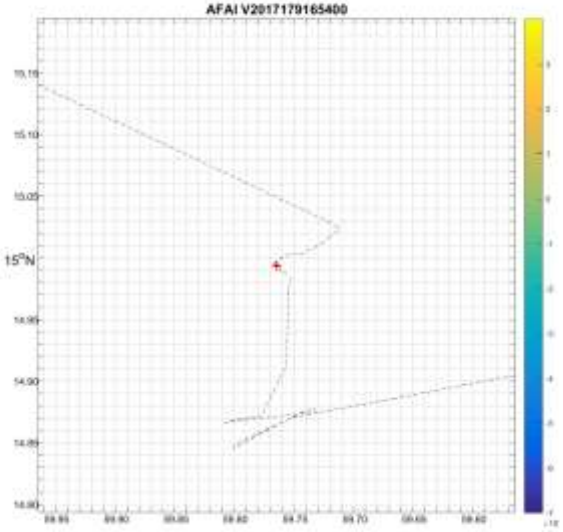

OLCI (300m) - 1 day

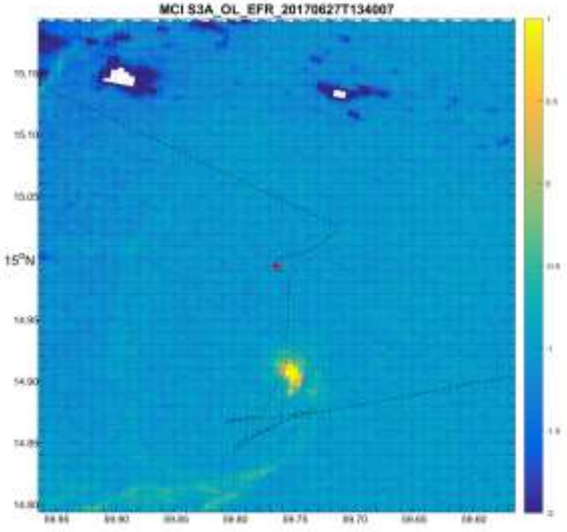

S2 Fig – 12. WEST ATLANTIC - S12 - 2017-06-29 13.58 UTC - 15°57.270'N 61°59.300'W - WS = 7.8 $m.s^{-1}$ WD = 277° SS = Slight

MODIS-A (1km)

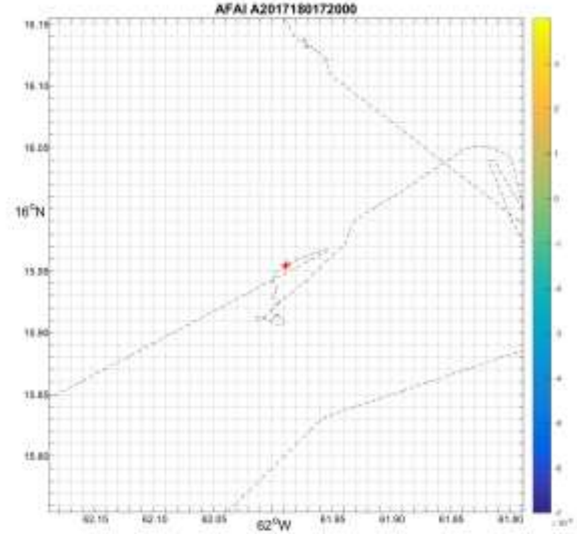

MODIS-T (1km)

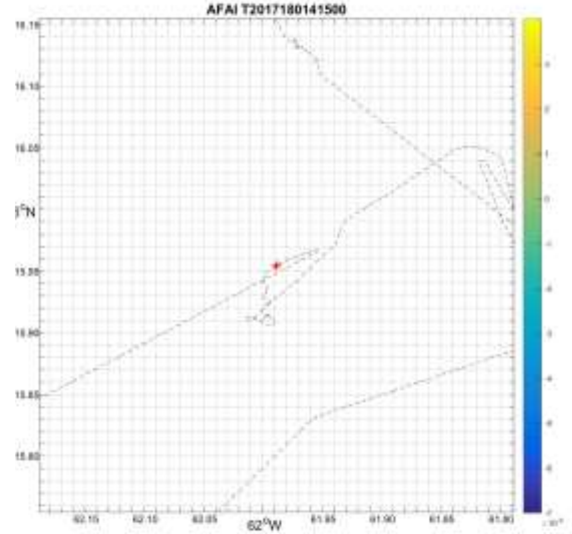

VIIRS (750 m)

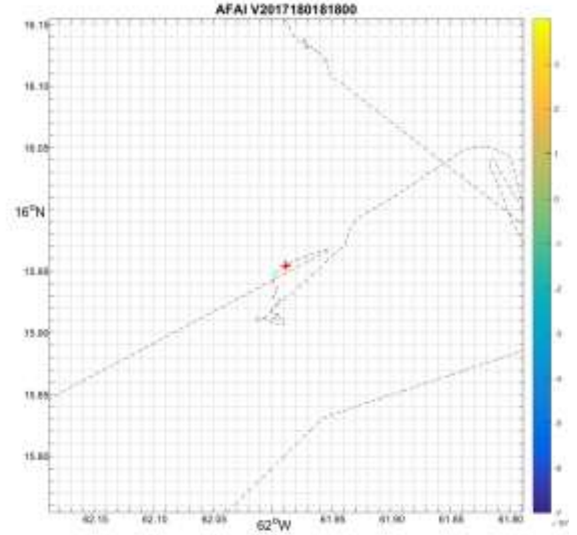

OLCI (300m)

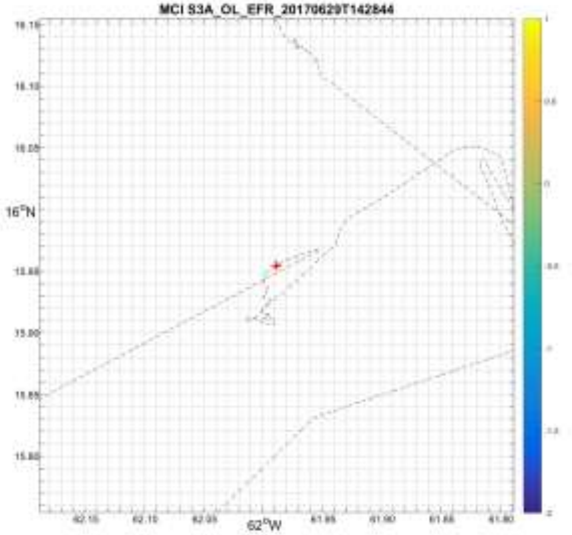

In situ - Type 4

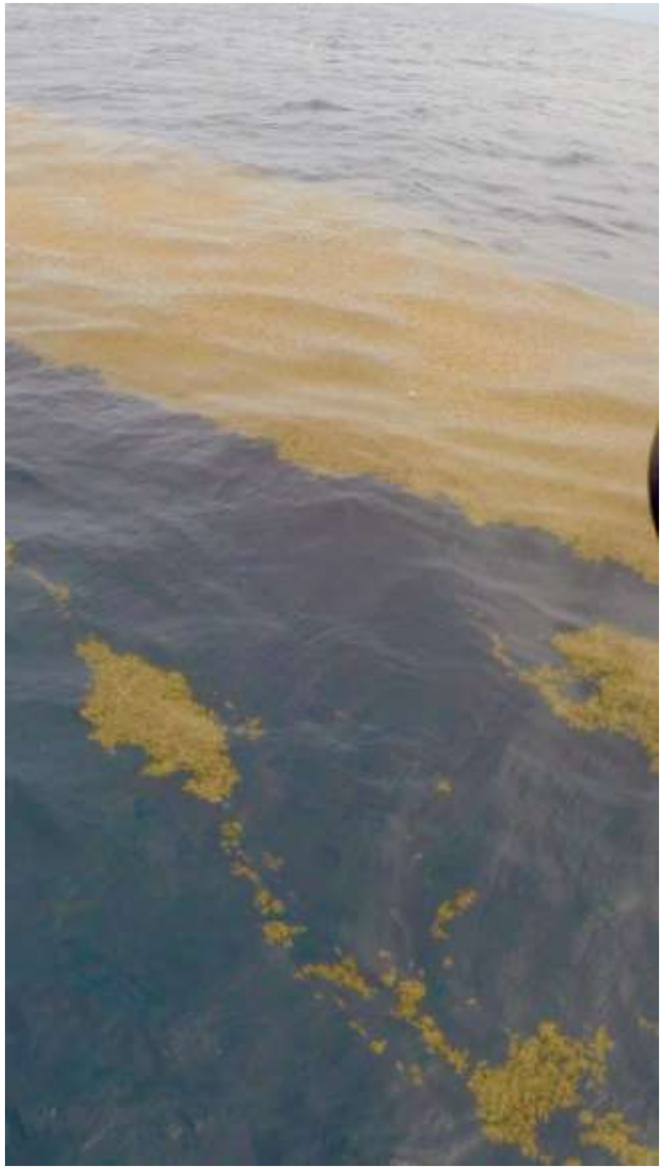

S2 Fig – 13. WEST ATLANTIC - S15 - 2017-07-03 12.39 UTC - 17°19.134'N 59°36.044'W - WS = 8.6 m.s^{-1} WD =256° SS =Moderate

MODIS-A (1km)

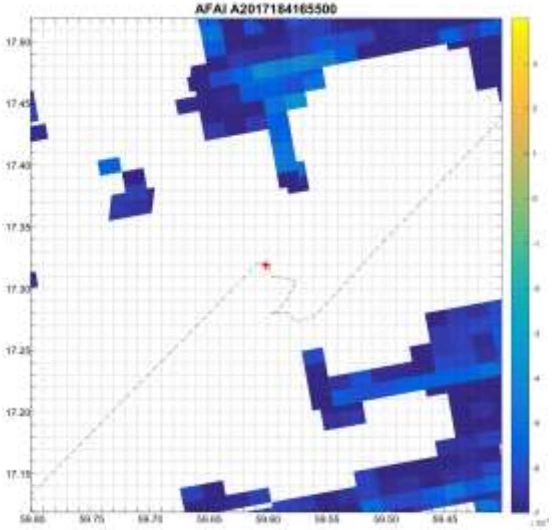

MODIS-T (1km)

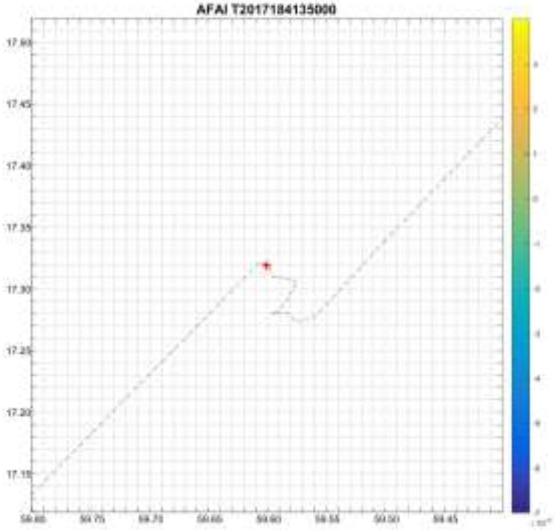

VIIRS (750 m)

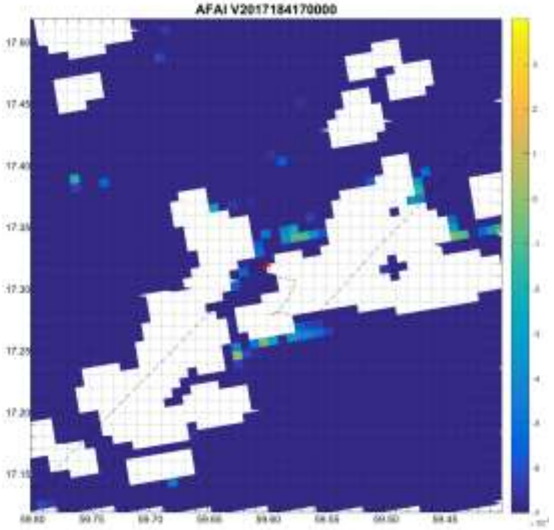

OLCI (300m) + 1 day

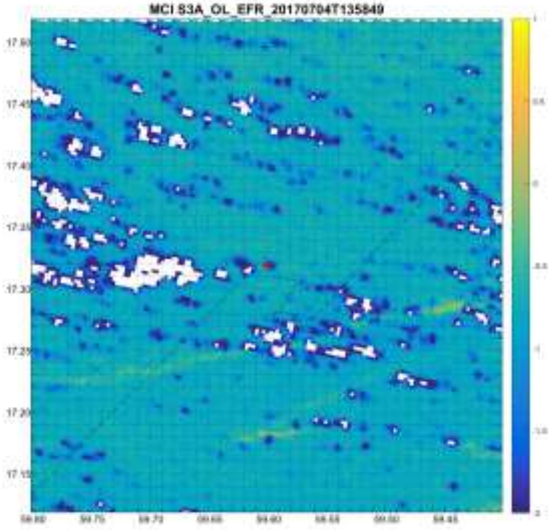

In situ - Type 3

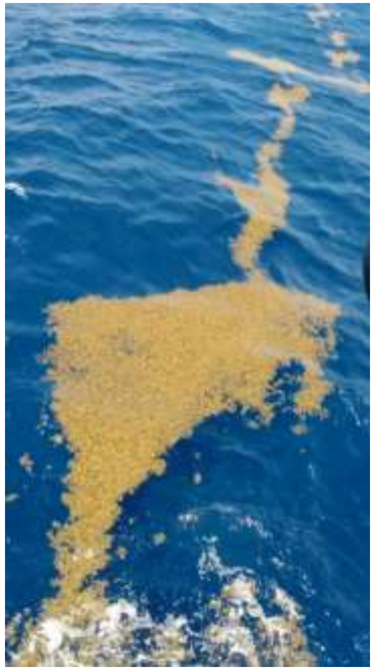

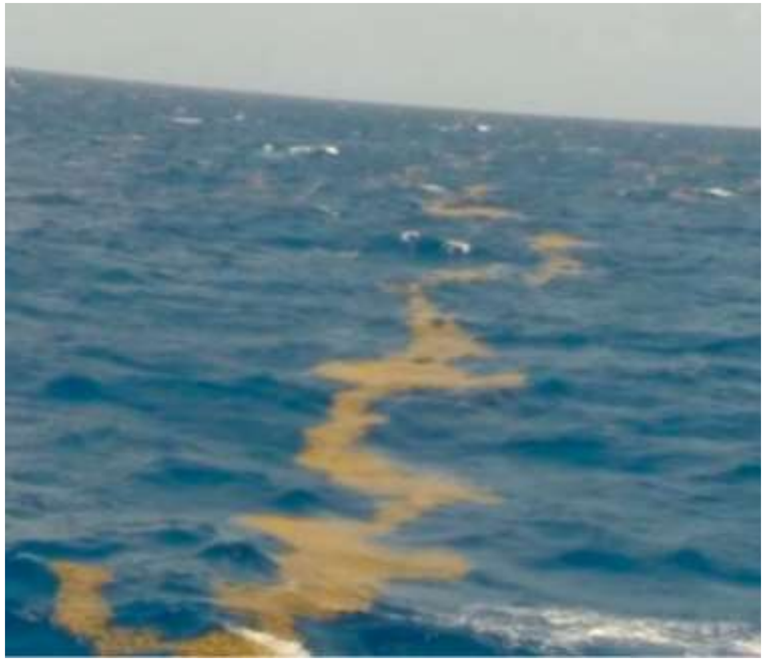

S2 Fig – 14. WEST ATLANTIC - S16 - 2017-07-04 12.02 UTC - 19°40.327'N 58°41.059'W - WS = 8.8 m.s^{-1} WD =267° SS =Moderate

MODIS-A (1km)

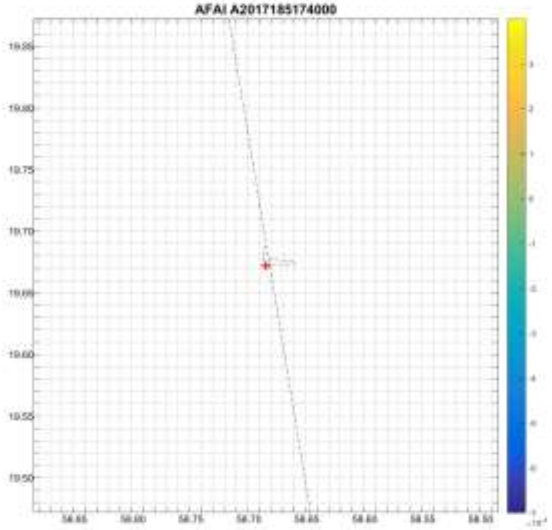

MODIS-T (1km)

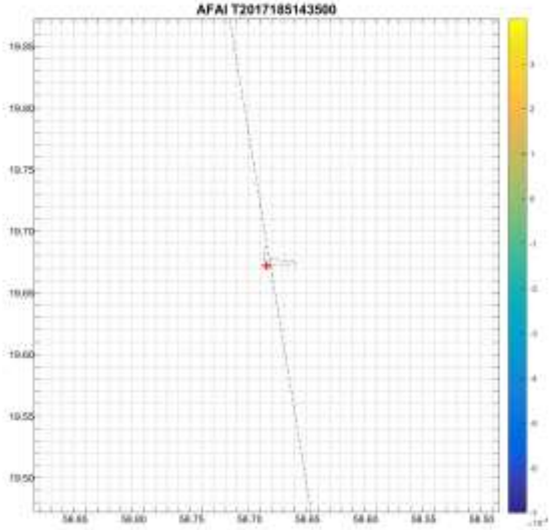

VIIRS (750 m)

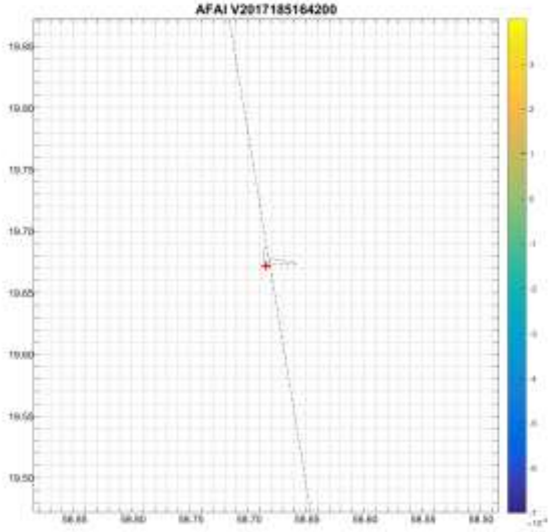

OLCI (300m)

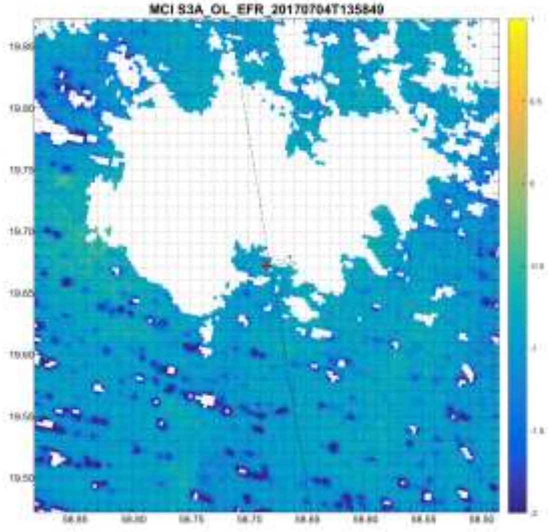

In situ - Type 2

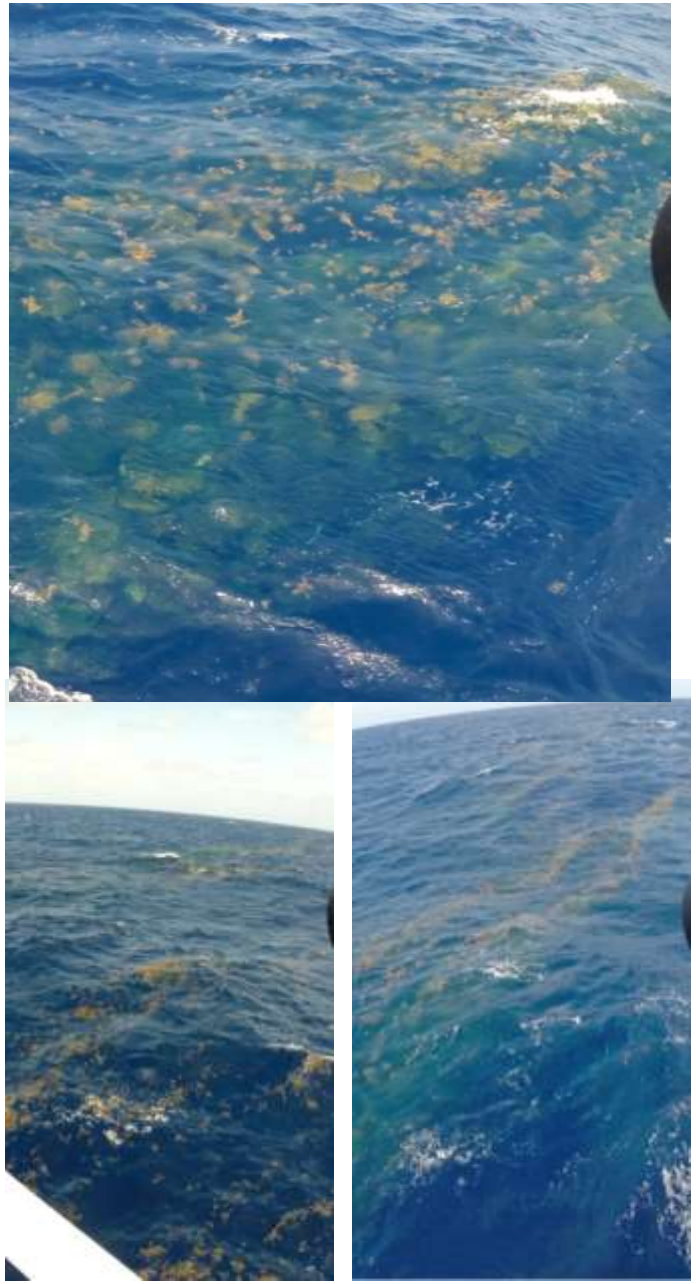

S2 Fig – 15. WEST ATLANTIC - S17 - 2017-07-04 20.39 UTC - 20°27.113'N 58°45.029'W - WS = 8.6 m.s^{-1} WD =270° SS = Moderate

MODIS-A (1km)

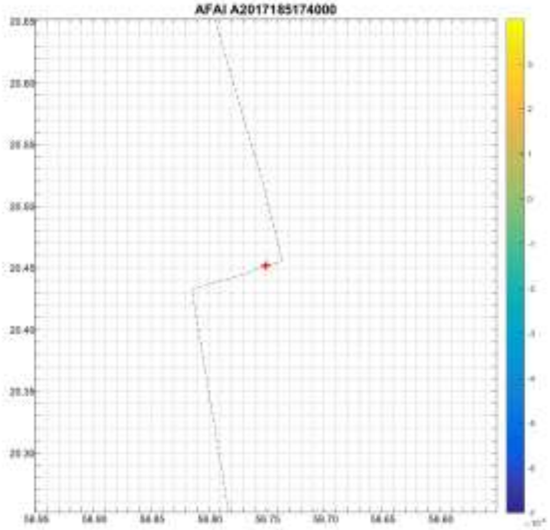

MODIS-T (1km)

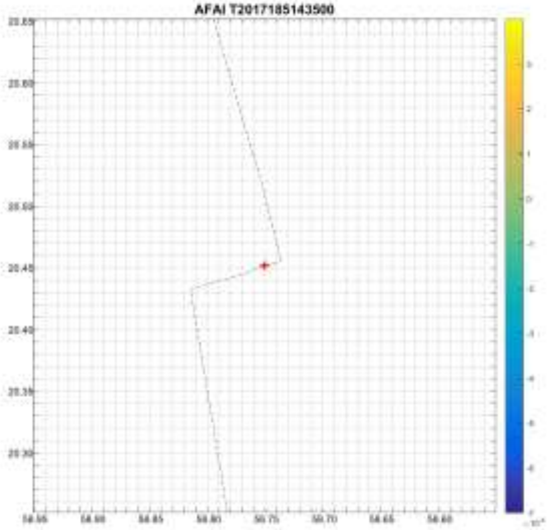

VIIRS (750 m)

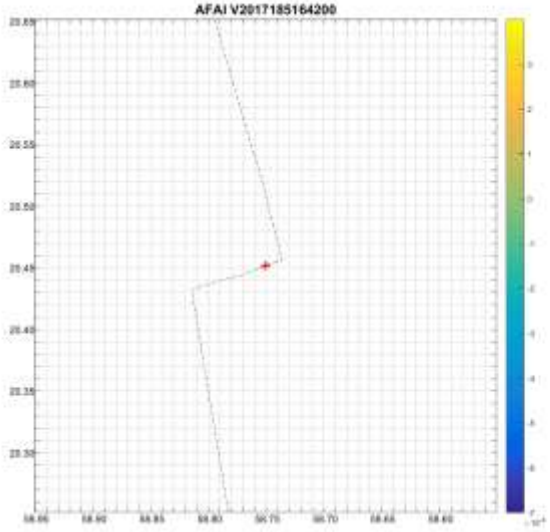

OLCI (300m)

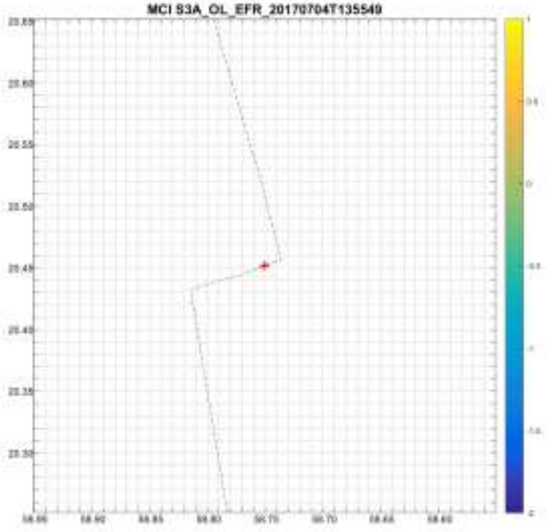

***In situ* - Type 1**

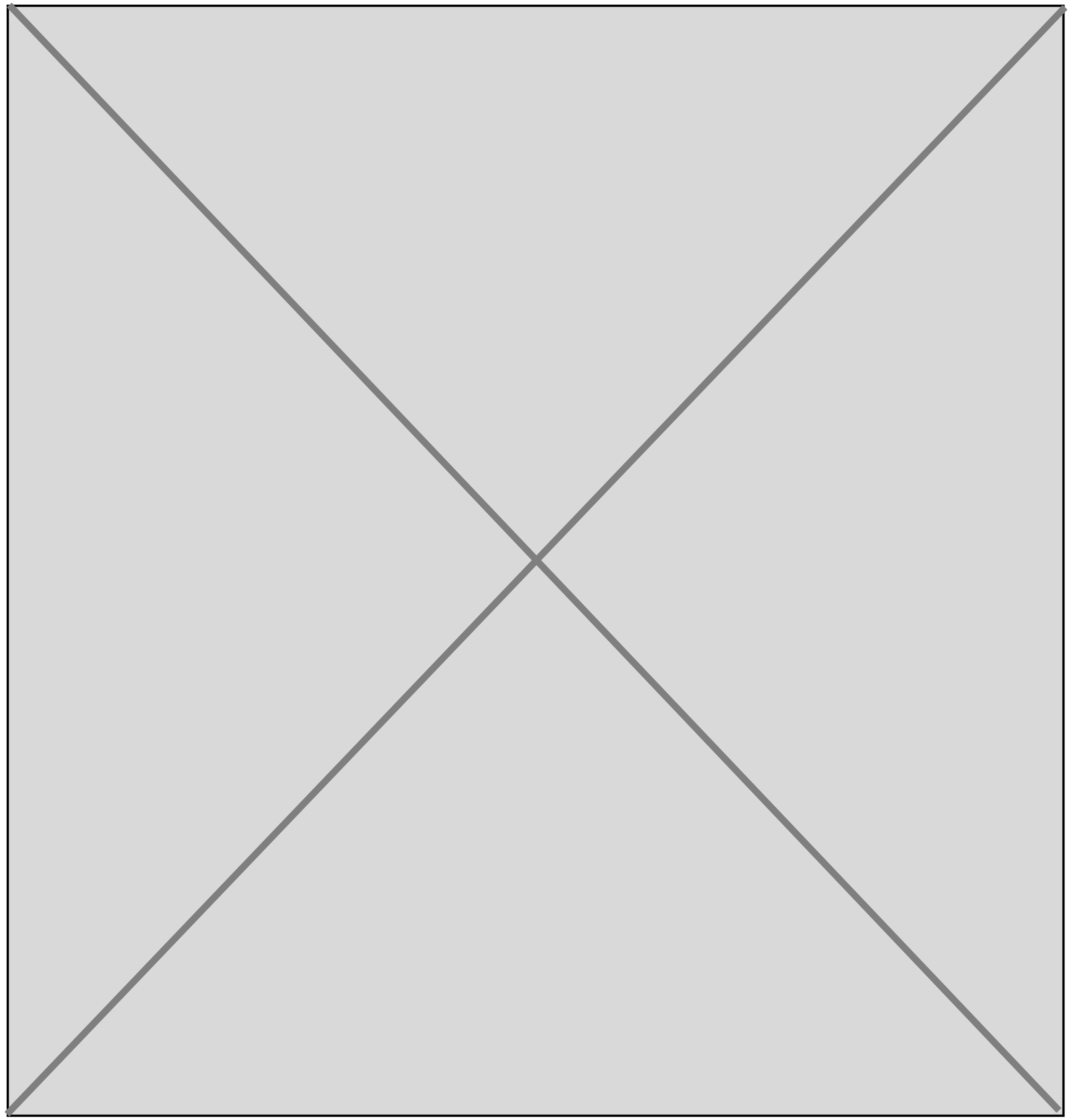

S2 Fig – 16. WEST ATLANTIC - S18 - 2017-07-05 11.32 UTC - 22°34.038'N 59°11.416'W - WS = 7.2 m.s^{-1} WD =270° SS = Slight

MODIS-A (1km)

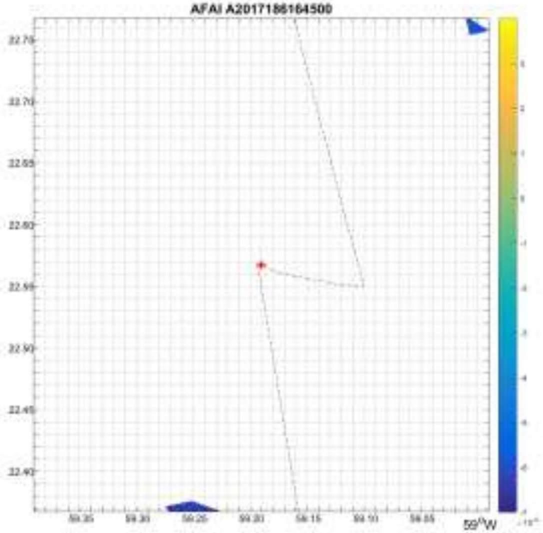

MODIS-T (1km)

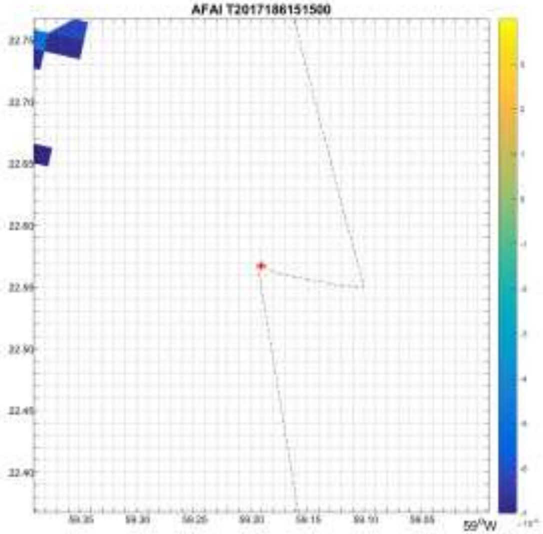

VIIRS (750 m)

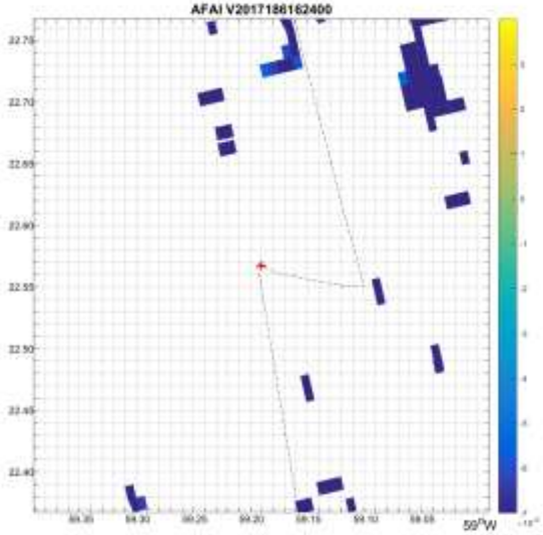

OLCI (300m) - 1 day

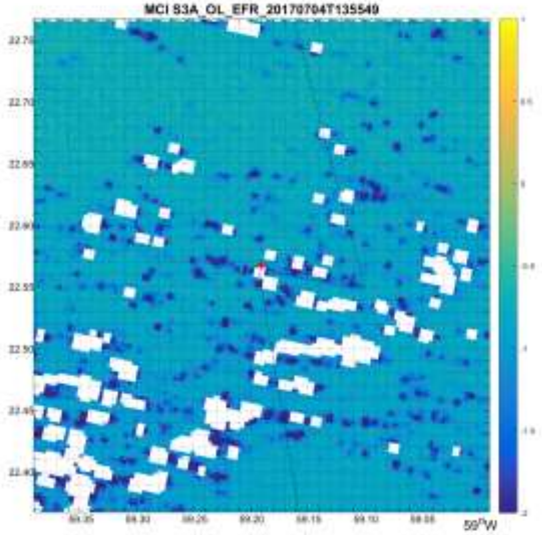

In situ - Type 1

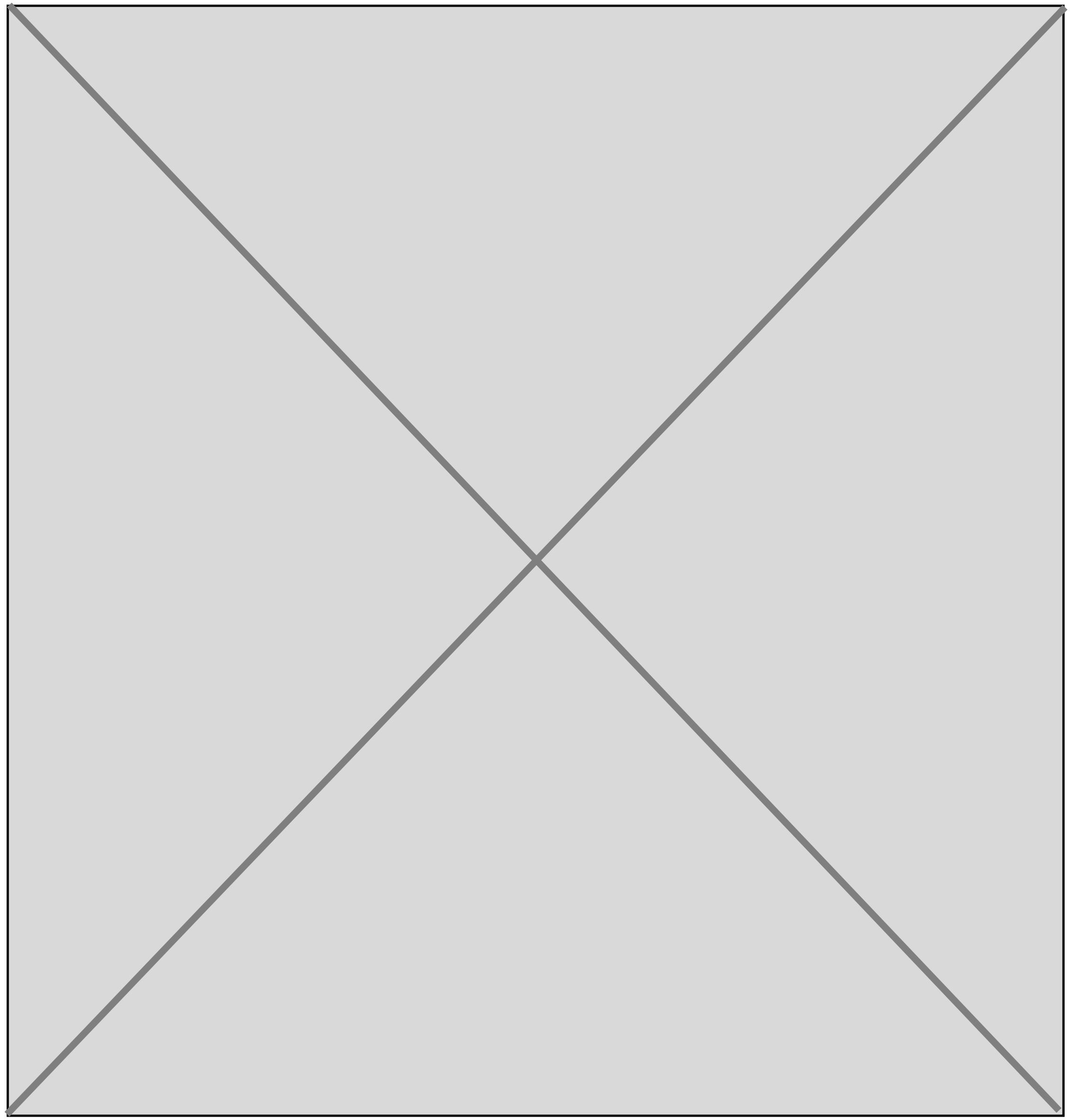

S2 Fig – 17. WEST ATLANTIC - S19 - 2017-07-05 18.35 UTC - 23°01.740'N 59°13.367'W - WS = 7.0 m.s^{-1} WD =274° SS = Slight

MODIS-A (1km)

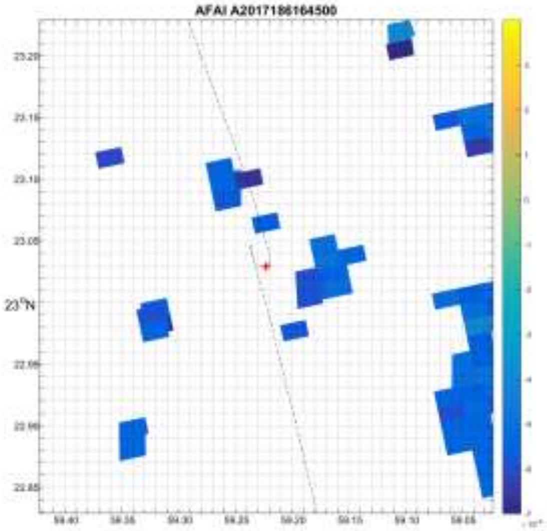

MODIS-T (1km)

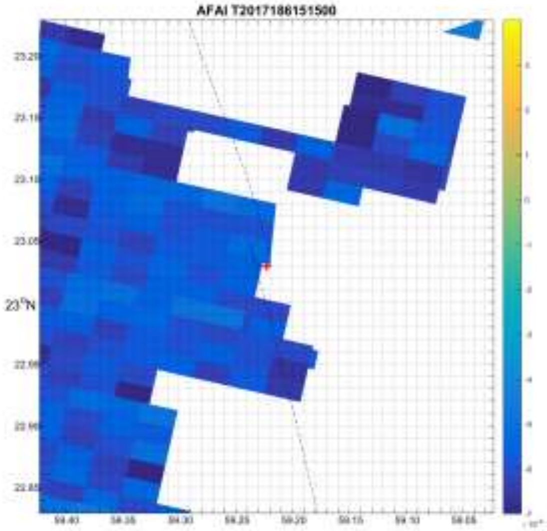

In situ - Type 2

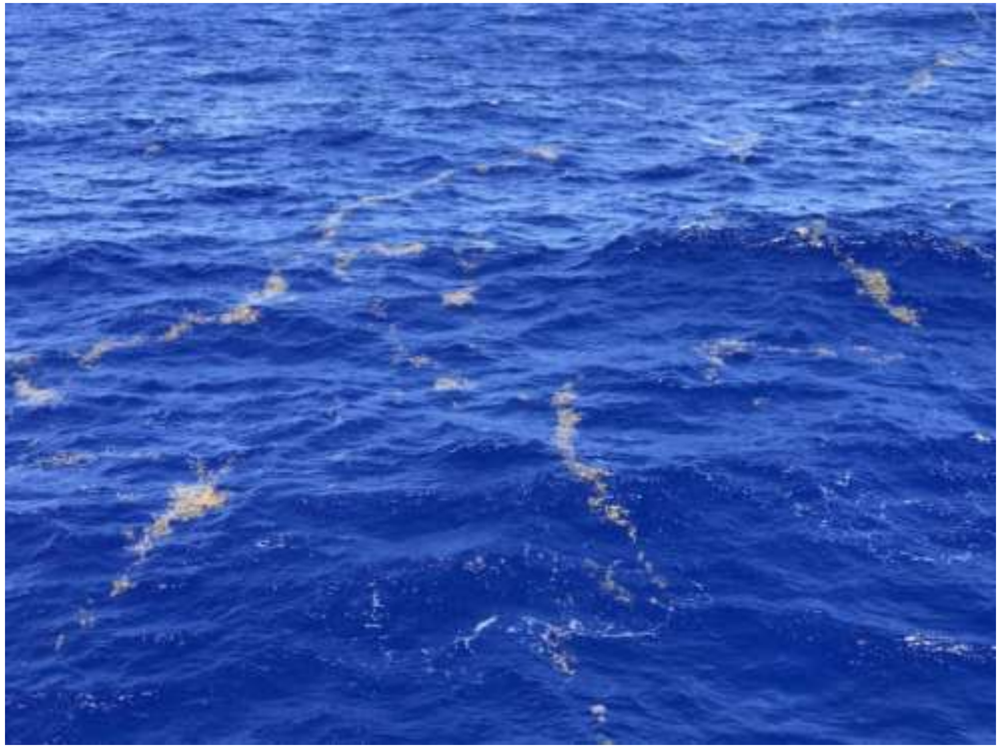

VIIRS (750 m)

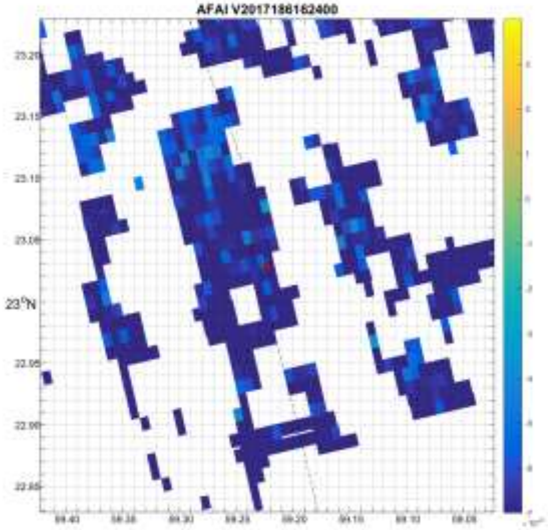

OLCI (300m) - 1 day

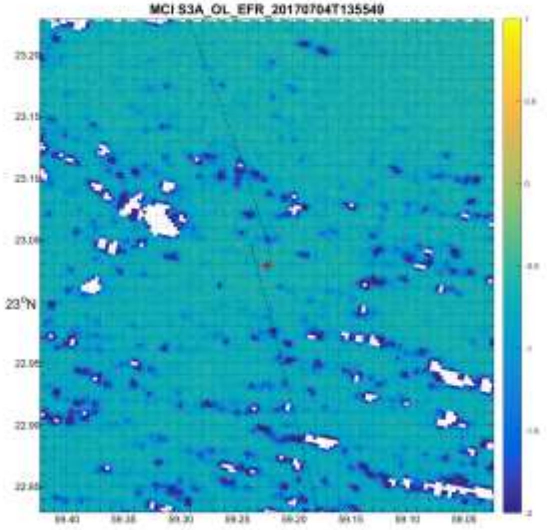

S2 Fig – 18. WEST ATLANTIC - S20 - 2017-07-06 11.40 UTC - 23°36.575'N 59°44.544'W - WS = 5.1 m.s^{-1} WD =273° SS = Slight

MODIS-A (1km)

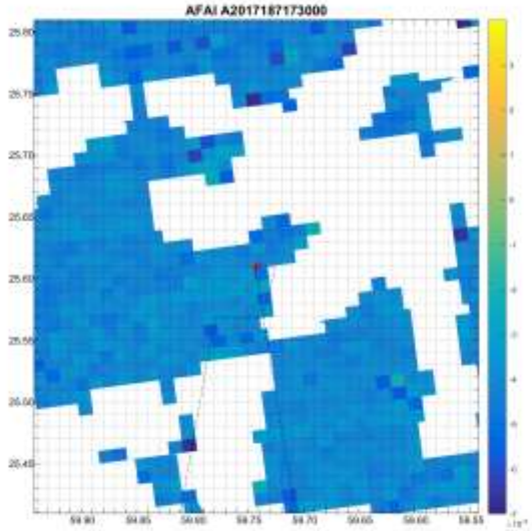

MODIS-T (1km)

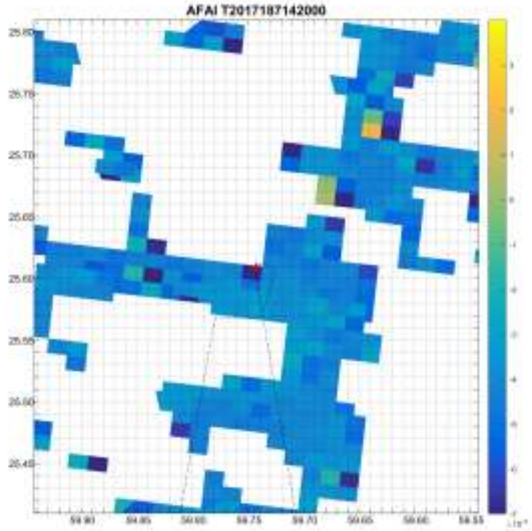

VIIRS (750 m)

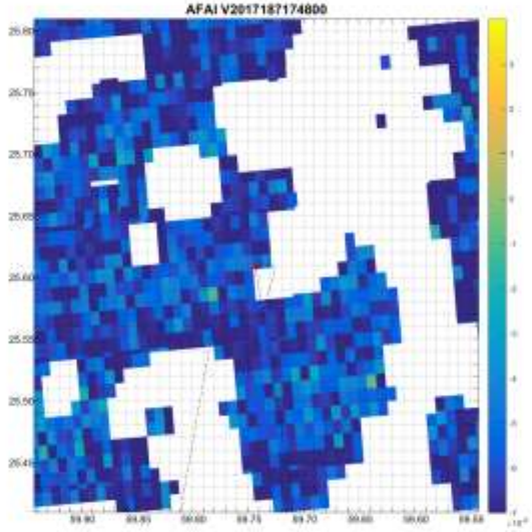

OLCI (300m) + 1 day

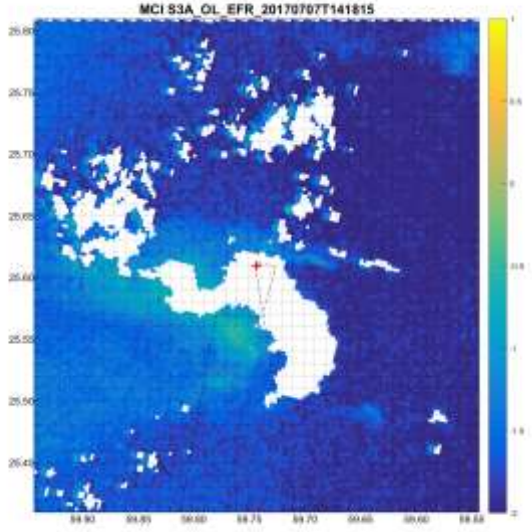

In situ - Type 1

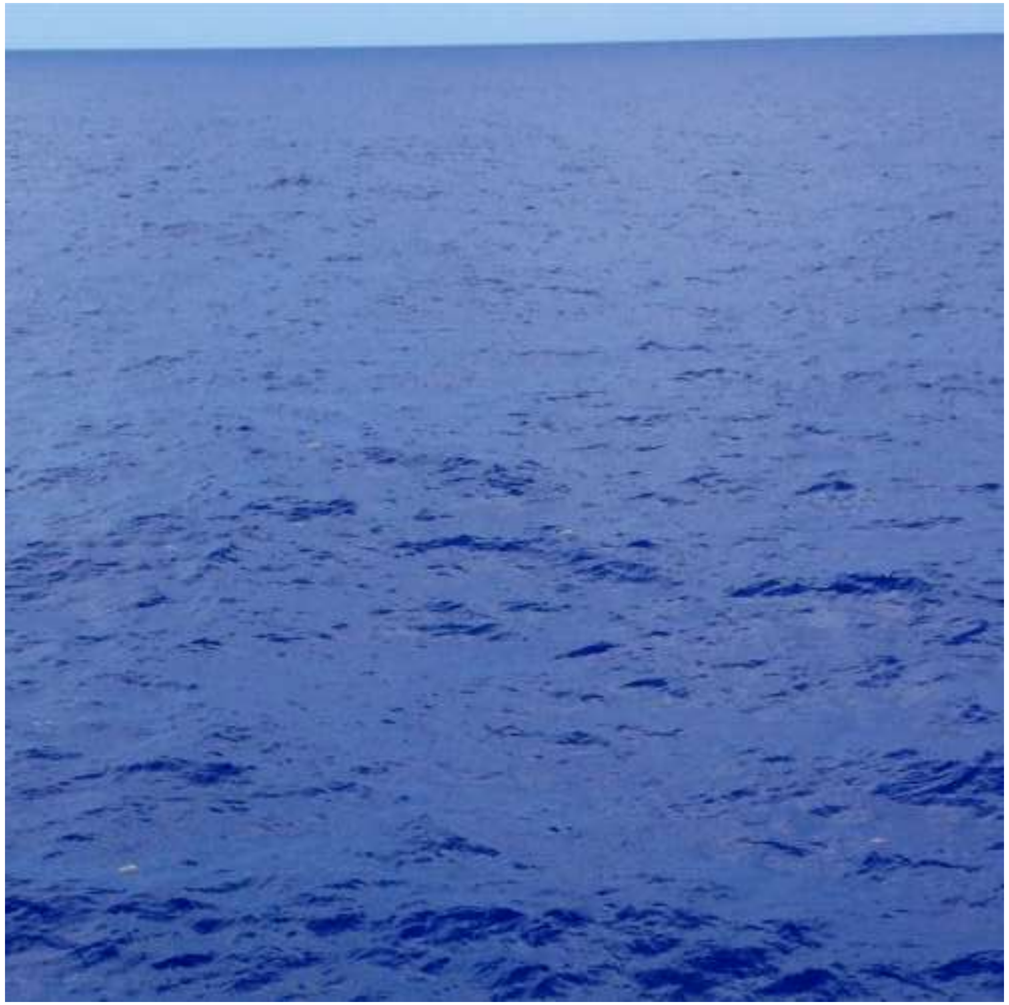

S2 Fig – 19. WEST ATLANTIC - S22 - 2017-07-07 18.02 UTC - 21°38.080'N 60°32.705'W - WS = 8.0 m.s^{-1} WD =268° SS = Moderate

MODIS-A (1km)

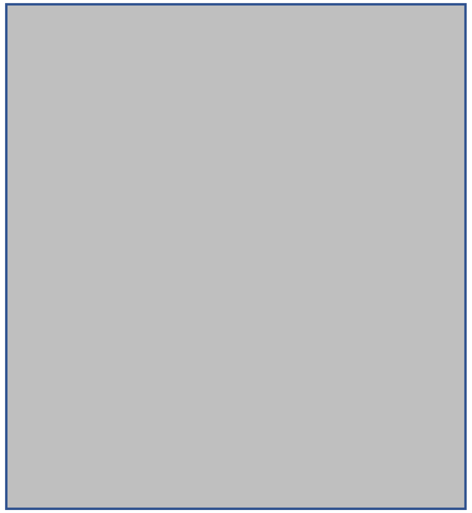

MODIS-T (1km)

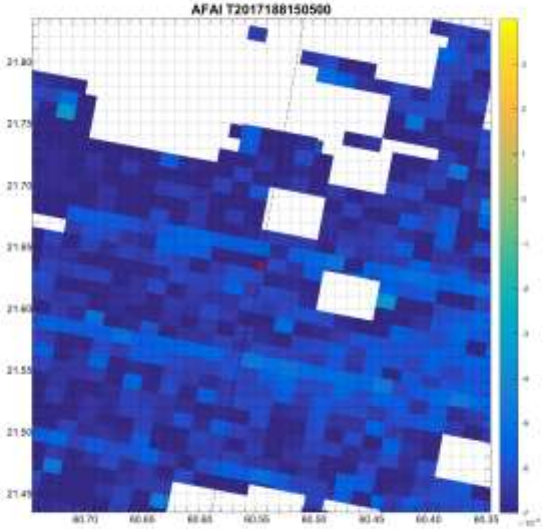

In situ - Type 2

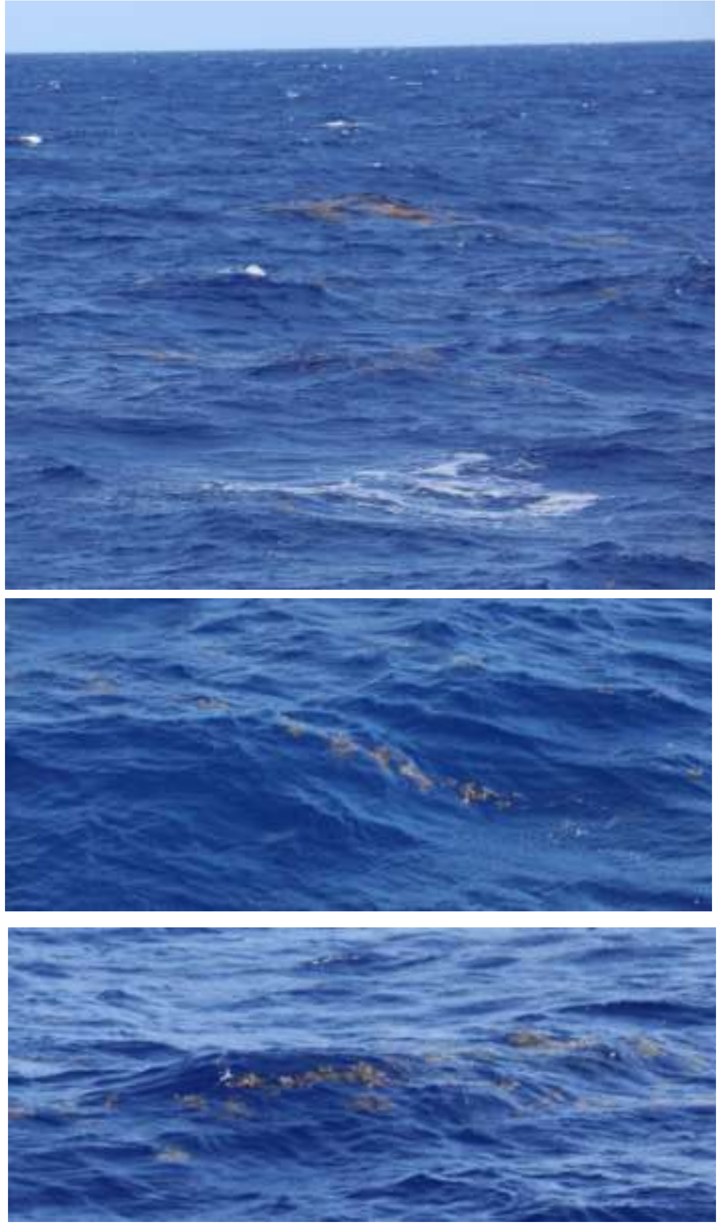

VIIRS (750 m)

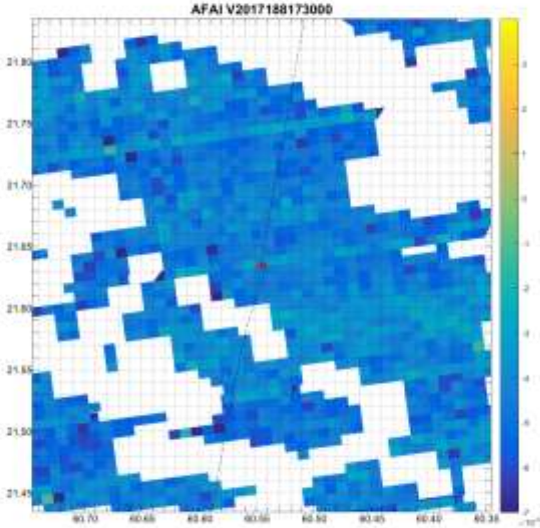

OLCI (300m)

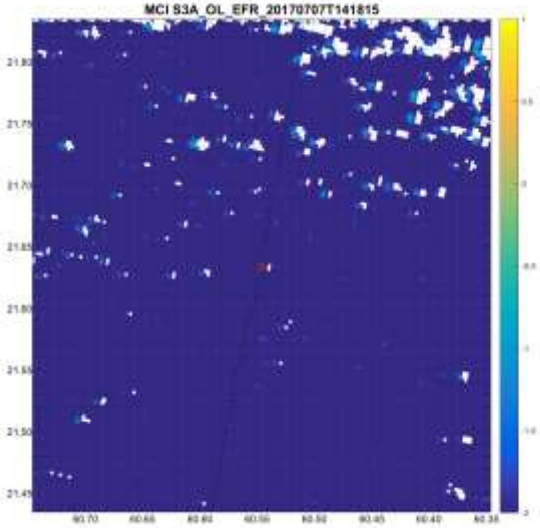

Affected by glint (diminish the MCI value)

S2 Fig – 20. WEST ATLANTIC - S23 - 2017-07-09 14.44 UTC - 21°38.080'N 60°32.705'W - WS = 3.6 $m.s^{-1}$ WD =314° SS = Smooth

MODIS-A (1km)

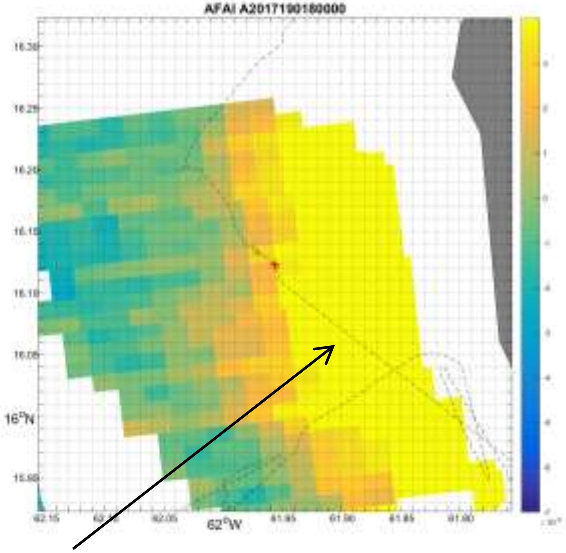

MODIS-T (1km)

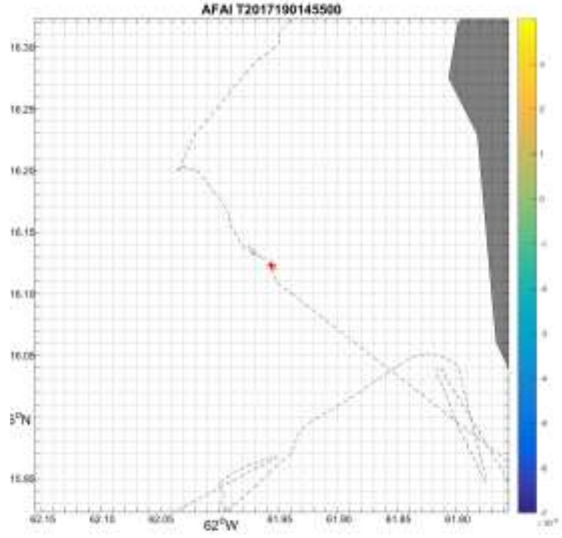

straylight contamination due to the proximity of land

VIIRS (750 m)

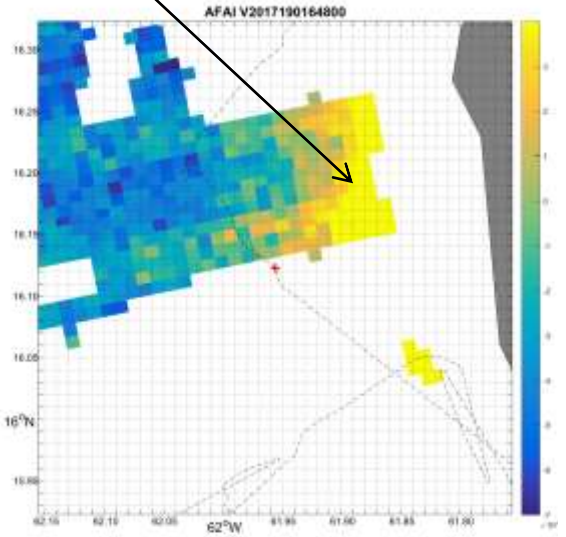

OLCI (300m) - 1 day

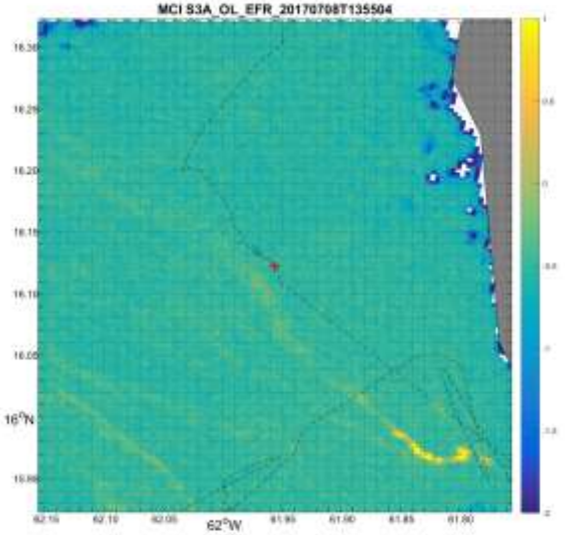

In situ - Type 4

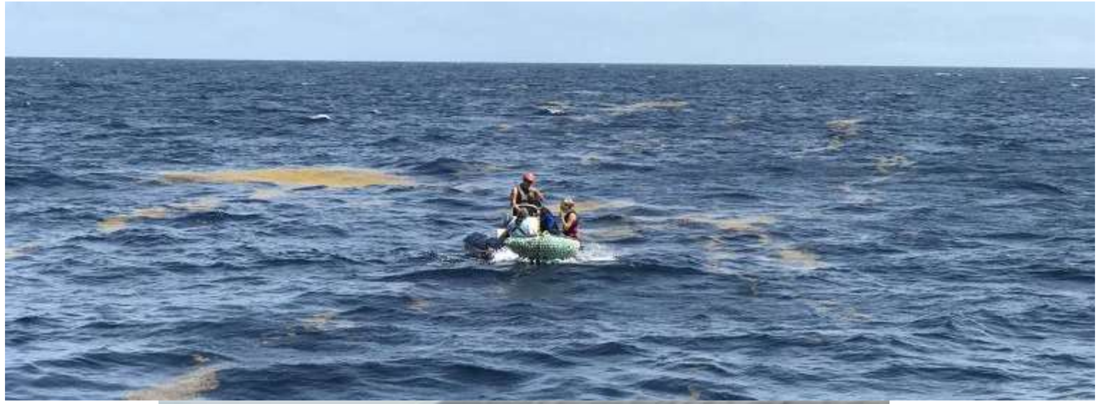

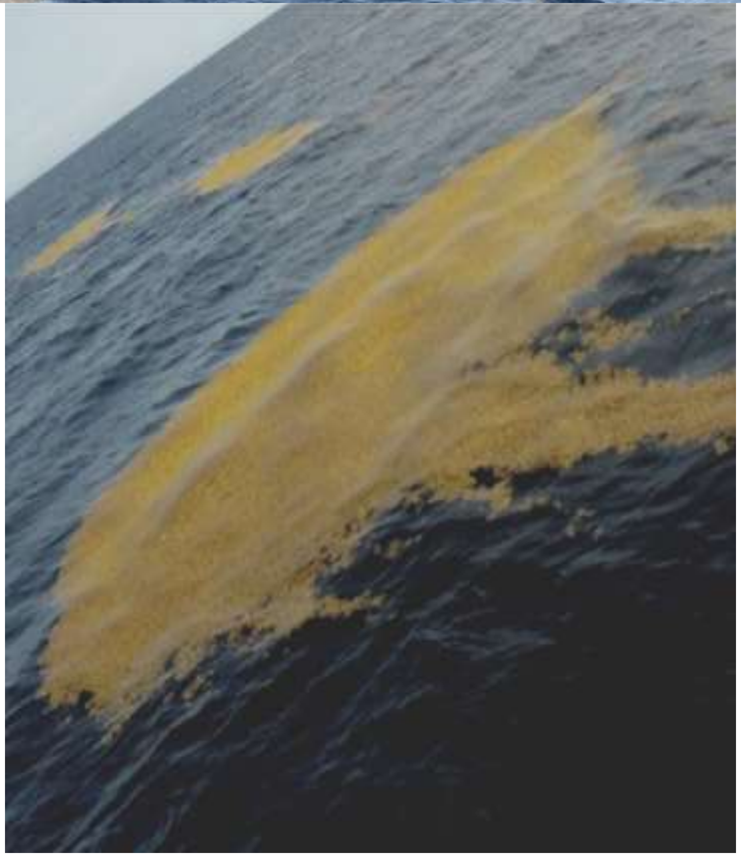

S2 Fig – 21. WEST ATLANTIC - S24a - 2017-07-09 16.30 UTC - 15°56.023'N 61°30.721'W - WS =4.1 m.s^{-1} WD =288° SS = Slight

MODIS-A (1km)

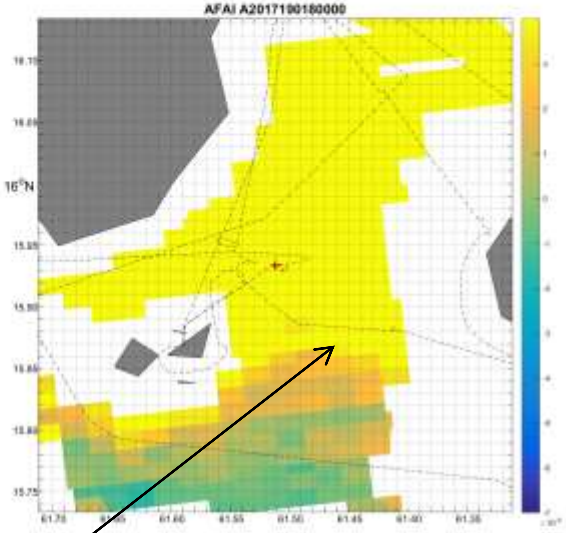

MODIS-T (1km)

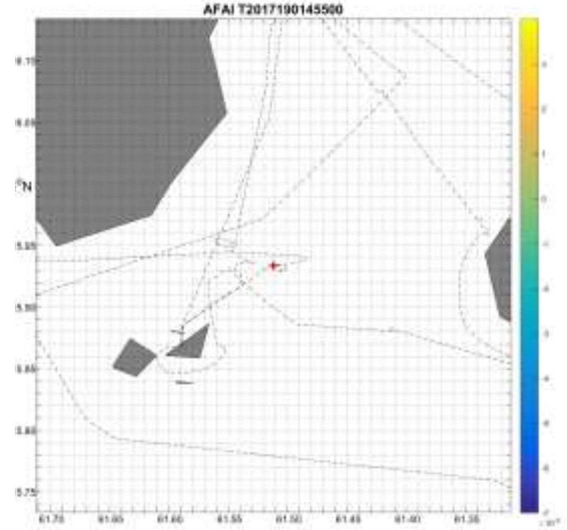

straylight contamination due to the proximity of land

VIIRS (750 m)

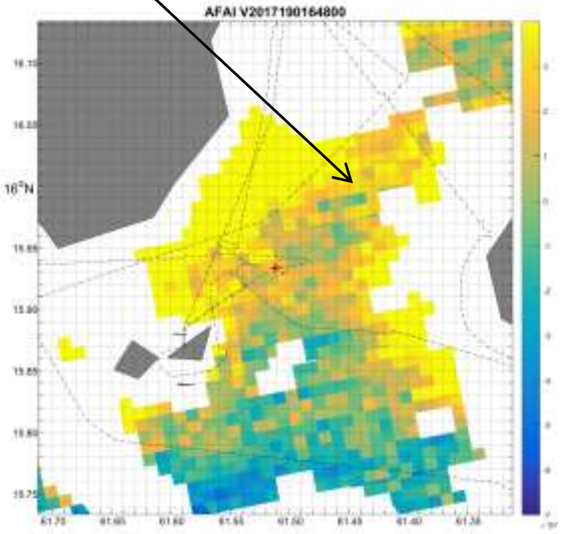

OLCI (300m) - 1 day

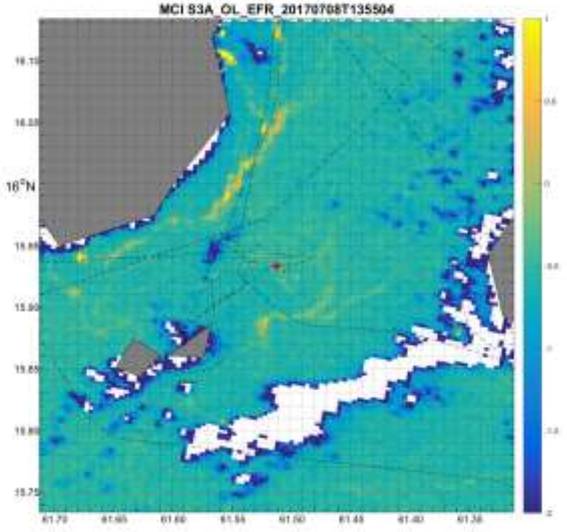

In situ - Type 5

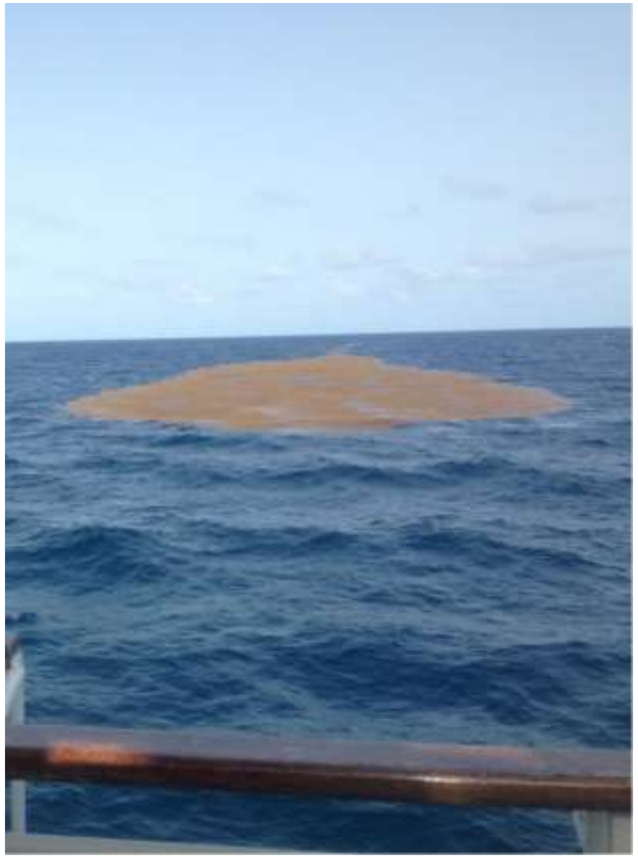

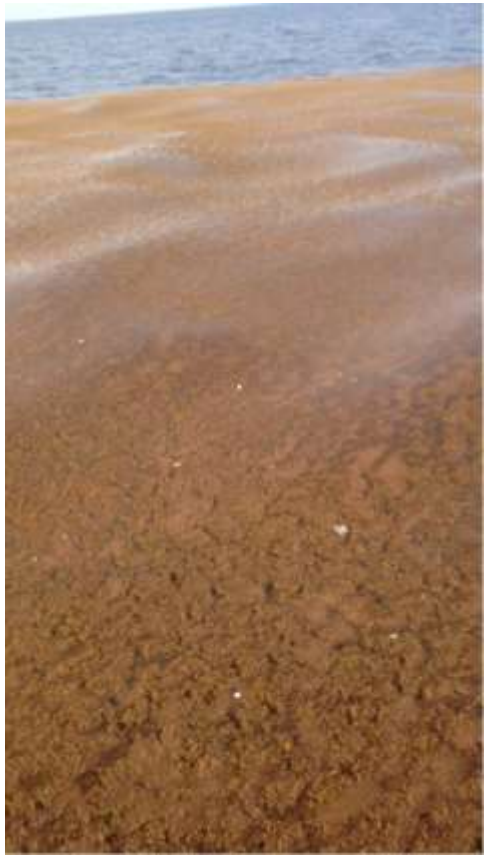

S2 Fig – 22. WEST ATLANTIC - S24b - 2017-07-10 12.54 UTC - 15°56.087'N 61°32.602'W - WS =5.5 m.s^{-1} WD =260° SS = Smooth

MODIS-A (1km)

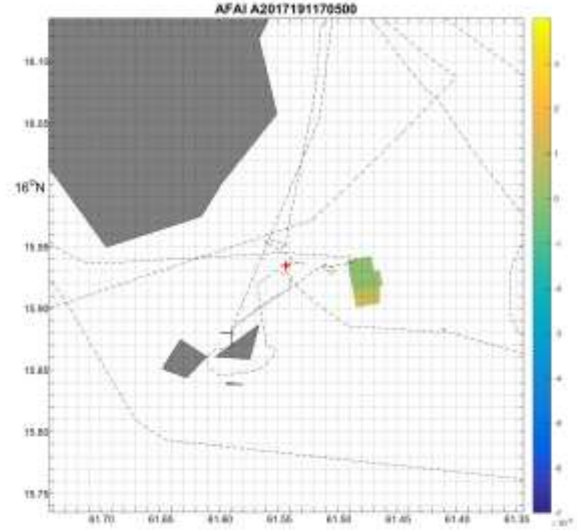

MODIS-T (1km)

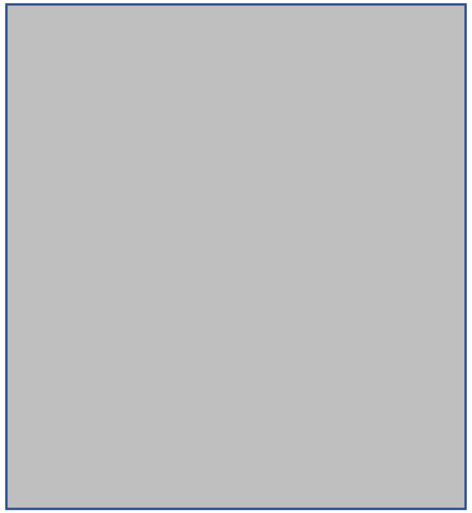

In situ - Type 4

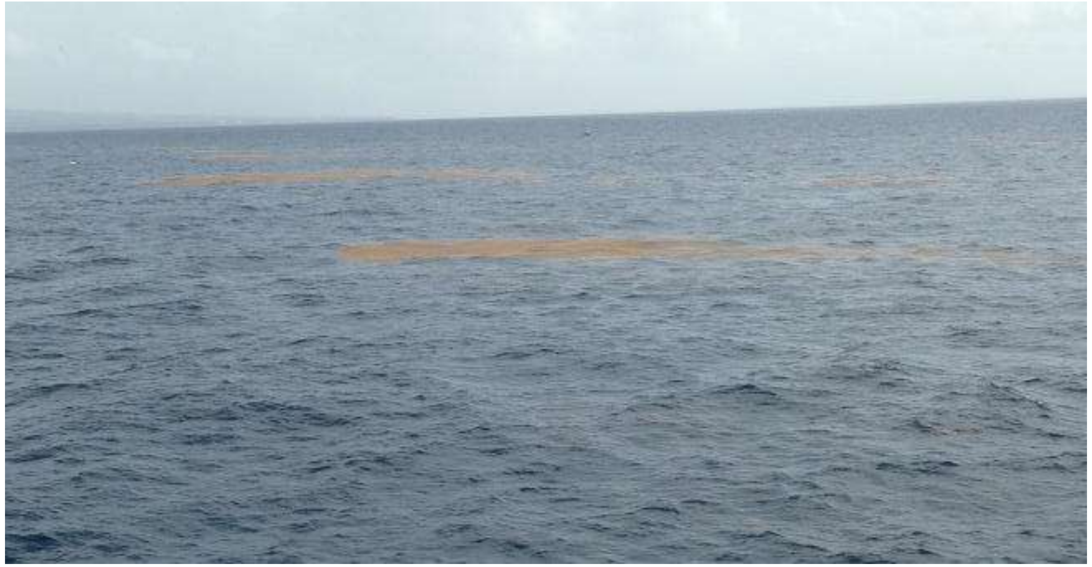

VIIRS (750 m)

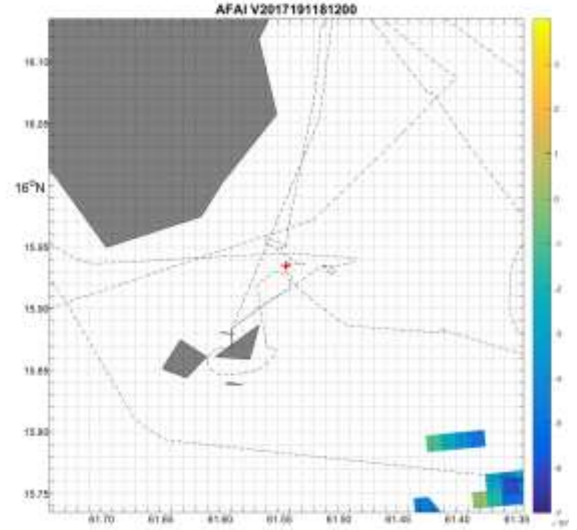

OLCI (300m) + 1 day

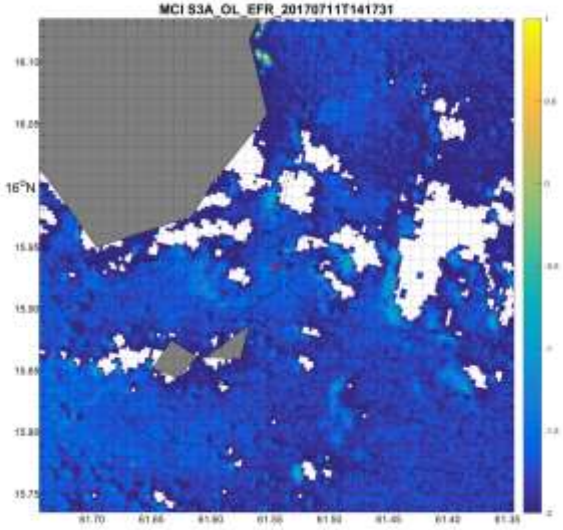

S2 Fig – 23. TRANSATLANTIC - Y01 - 2017-10-07 15.00 TU - 11°37.880'N 21°23.610'W - WS = 3.7 m.s^{-1} WD = 170° SS = Slight

MODIS-A (1km) **AFAI**

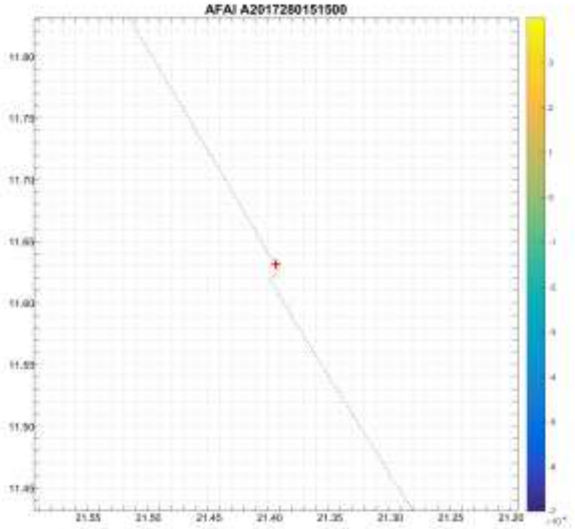

MODIS-T (1km) **AFAI**

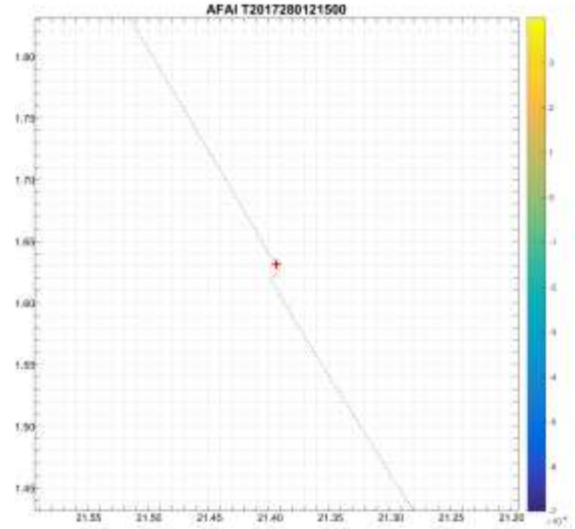

VIIRS (750 m) **AFAI**

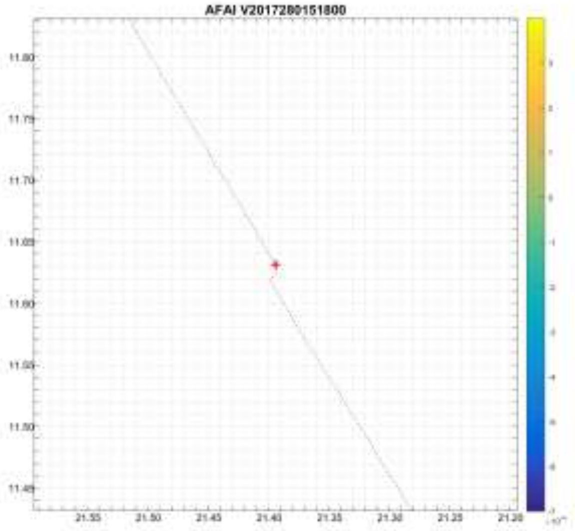

OLCI (300m) **MCI**

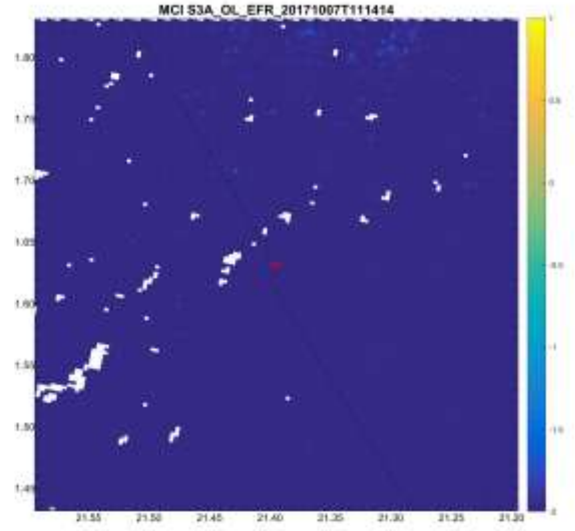

In situ - Type 1

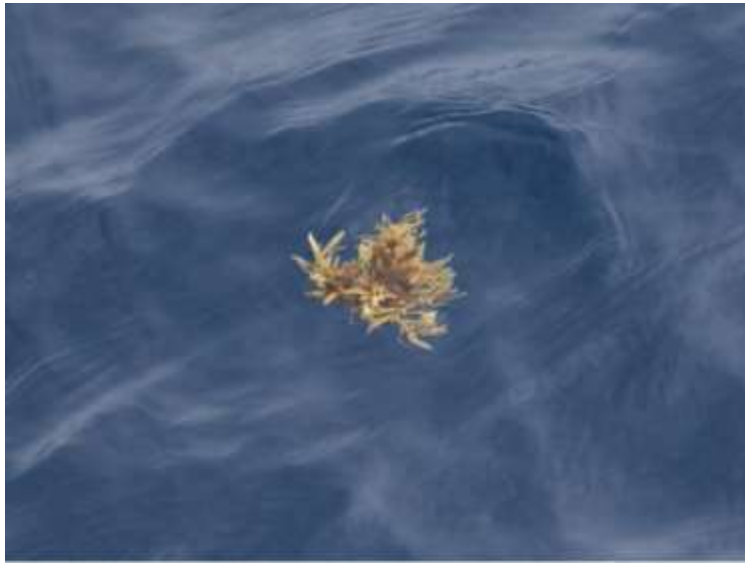

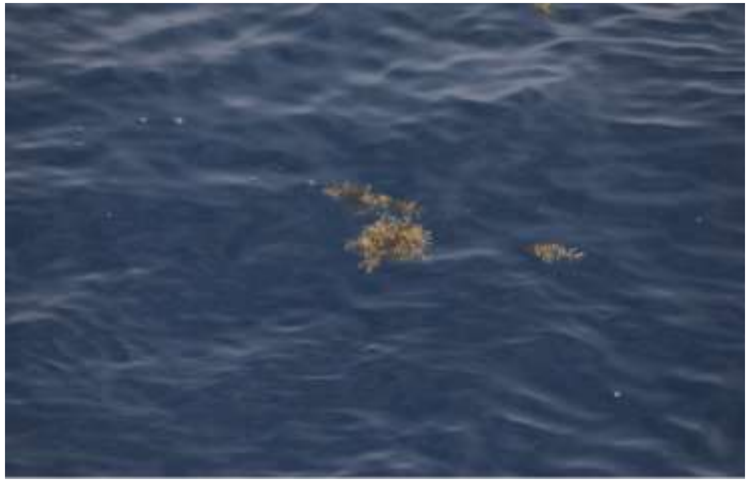

S2 Fig – 24. TRANSATLANTIC - Y02 - 2017-10-08 07.30 TU - 09°20.520'N 19°55.060'W - WS = 3.2 m.s^{-1} WD = 340° SS = Slight

MODIS-A (1km) ***AFAI***

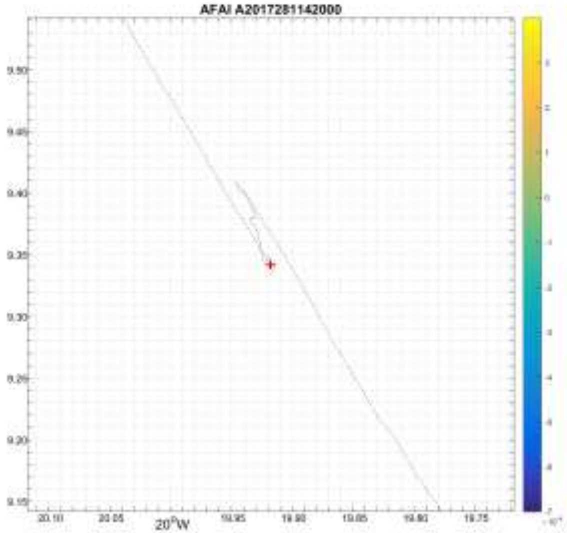

MODIS-T (1km) ***AFAI***

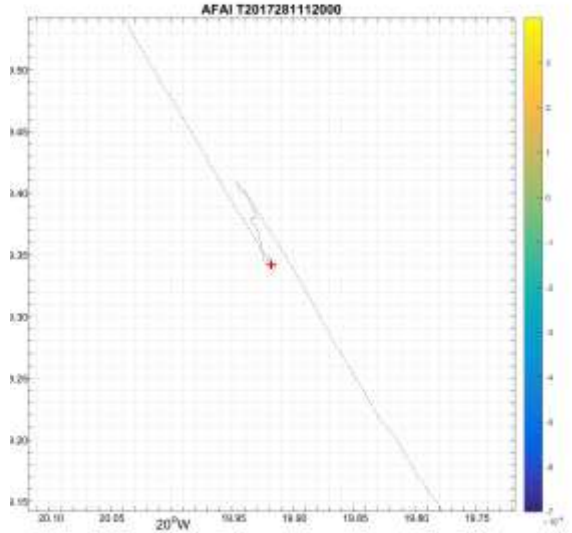

VIIRS (750 m) ***AFAI***

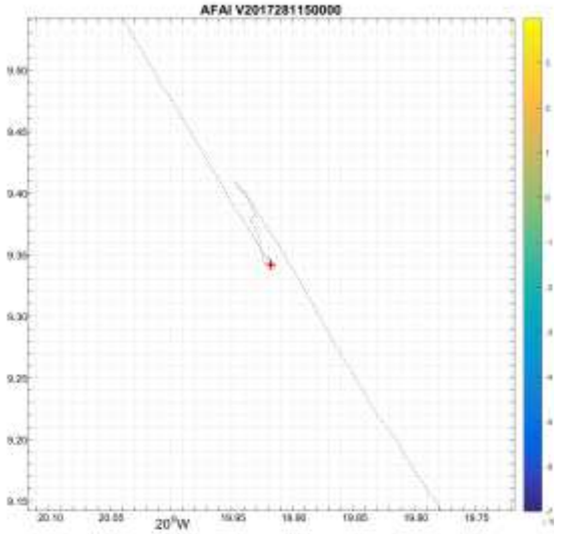

OLCI (300m) ***MCI***

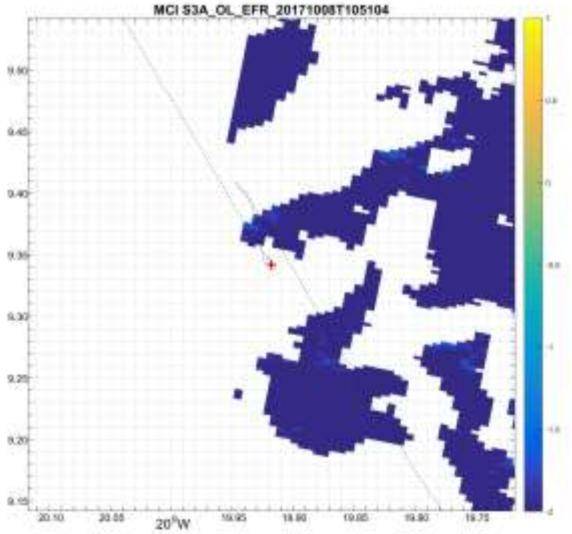

In situ - Type 5

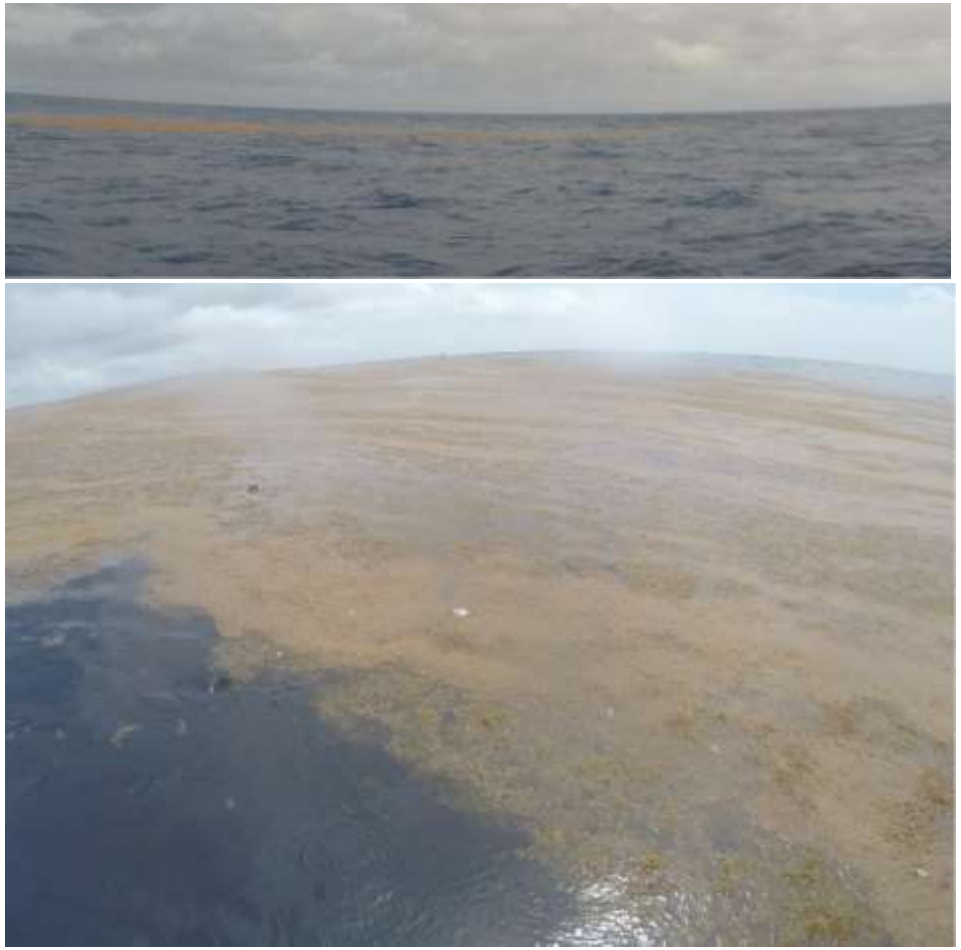

S2 Fig – 25. TRANSATLANTIC - Y03 - 2017-10-09 10.00 TU - 07°46.315'N 18°59.865'W - WS = 3.2 $m.s^{-1}$ WD = 70° SS = Slight

MODIS-A (1km) **AFAI**

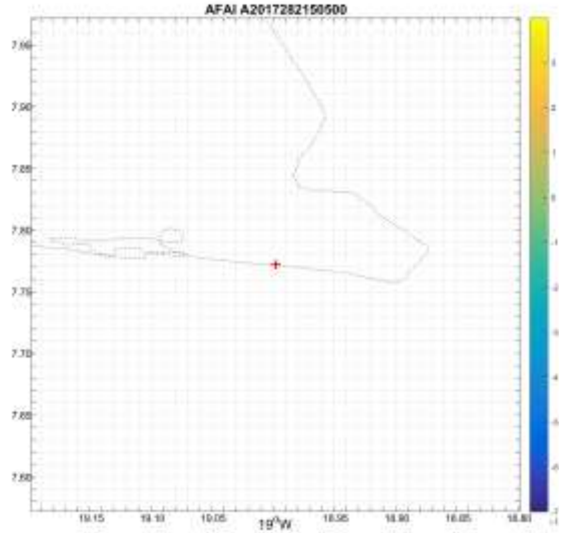

MODIS-T (1km) **AFAI**

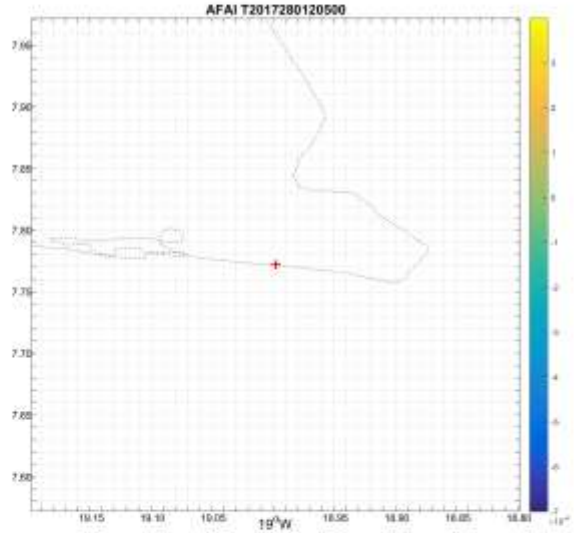

VIIRS (750 m) **AFAI**

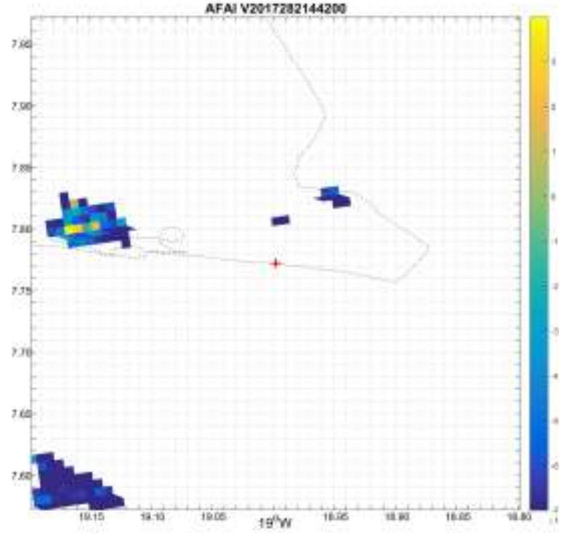

OLCI (300m) - 1 day **MCI**

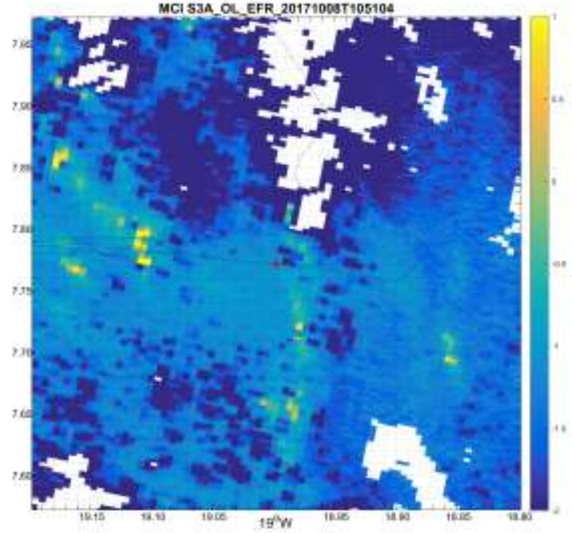

In situ - Type 3

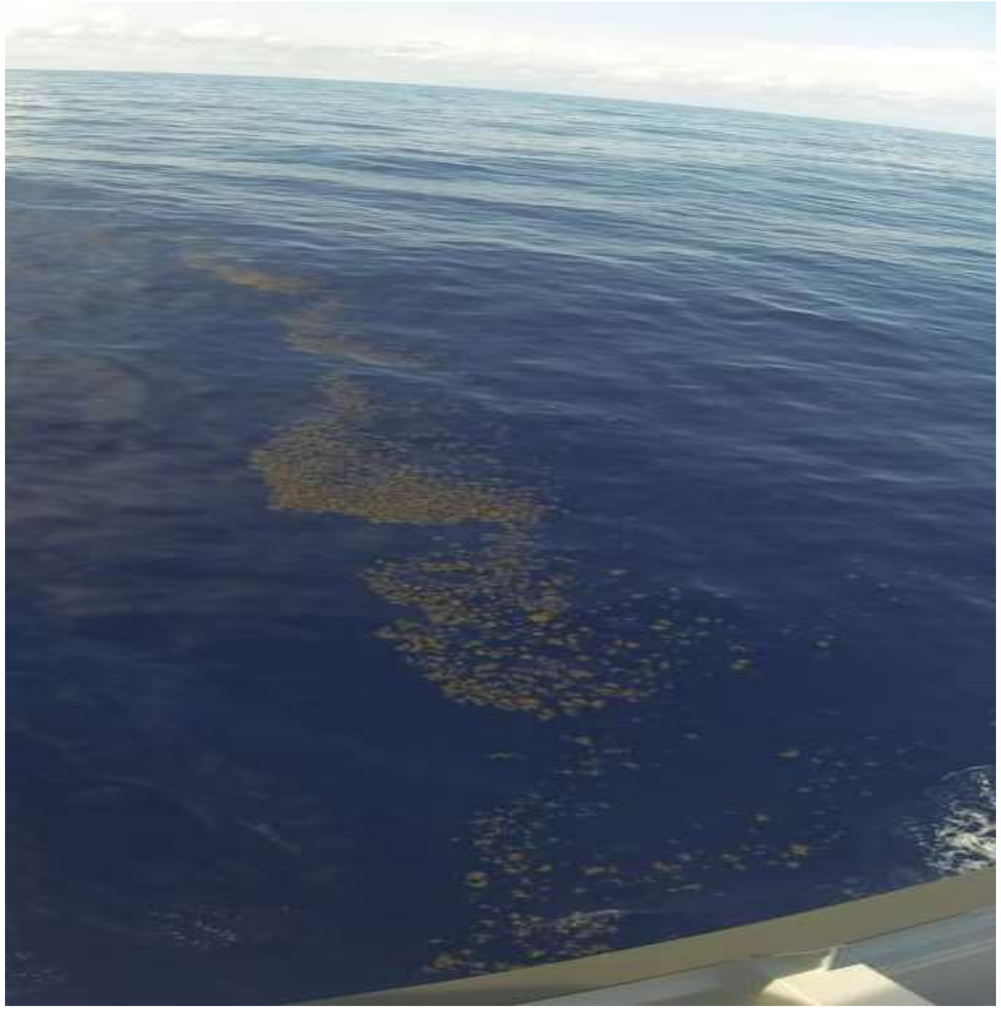

S2 Fig – 26. TRANSATLANTIC - Y03b - 2017-10-09 15.00 TU - 07°47.464'N 19°10.818'W - WS = 3.2 m.s^{-1} WD = 65° SS = Slight

MODIS-A (1km) ***AFAI***

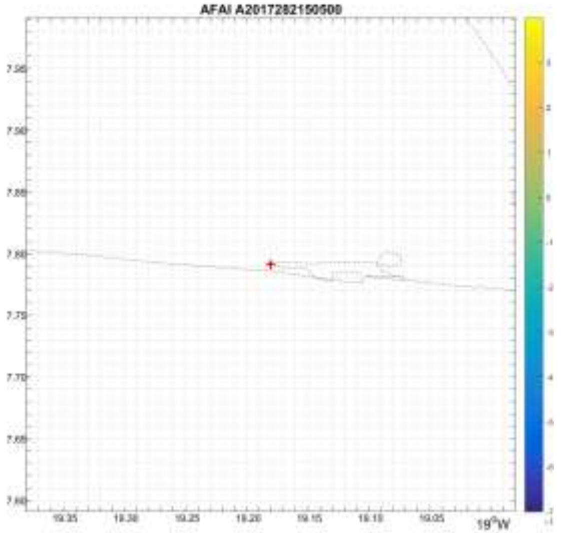

MODIS-T (1km) ***AFAI***

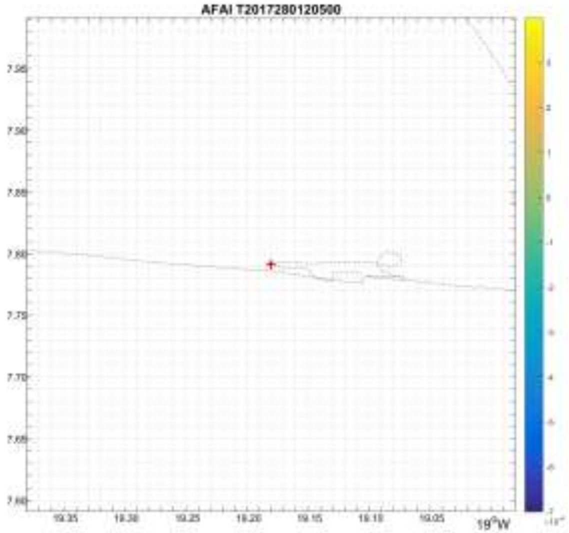

In situ - Type 4

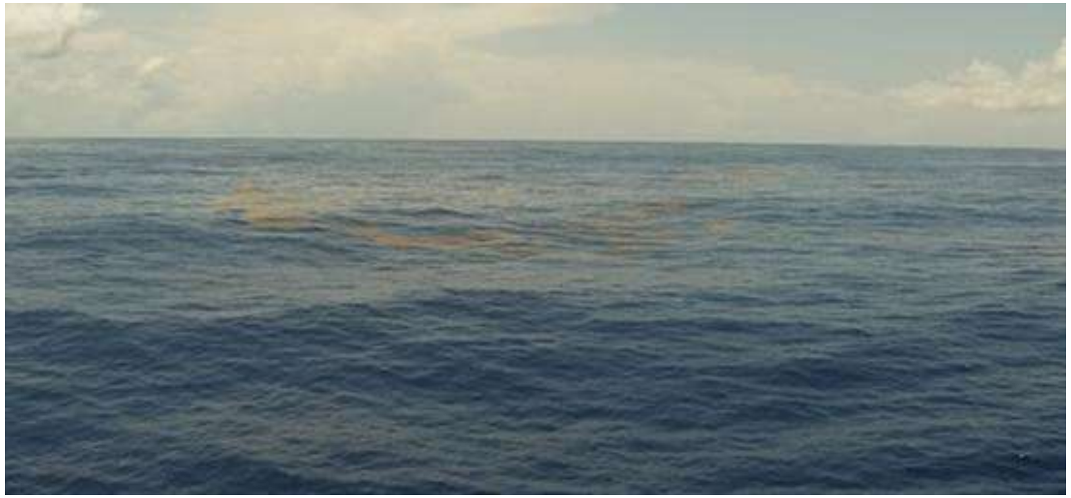

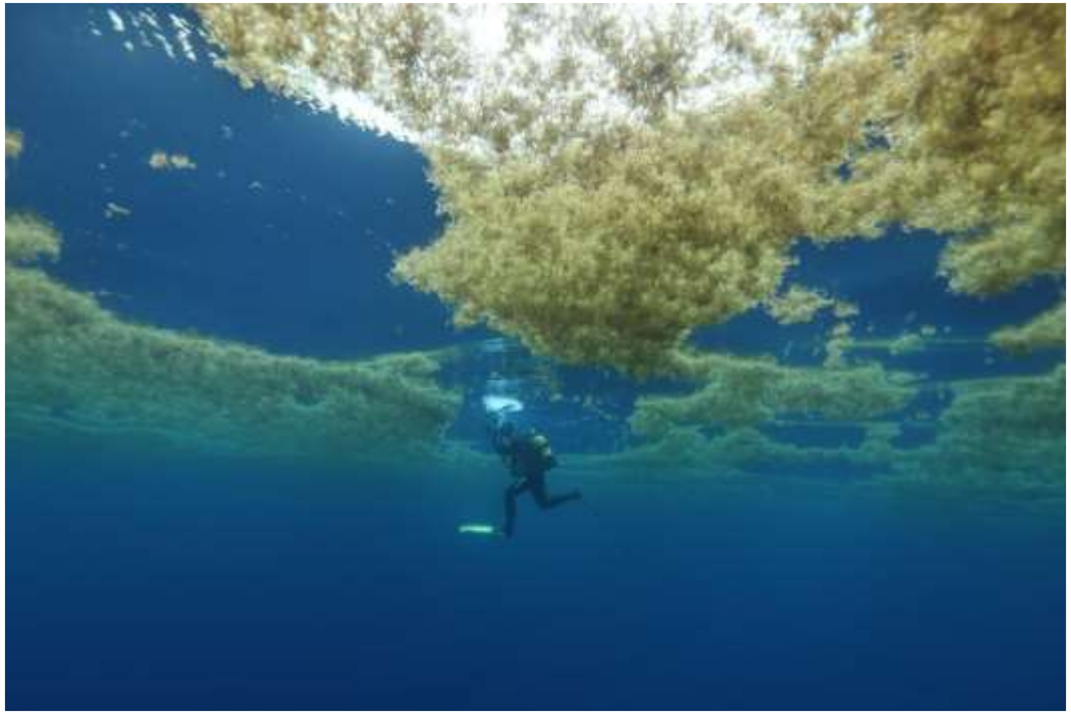

VIIRS (750 m) ***AFAI***

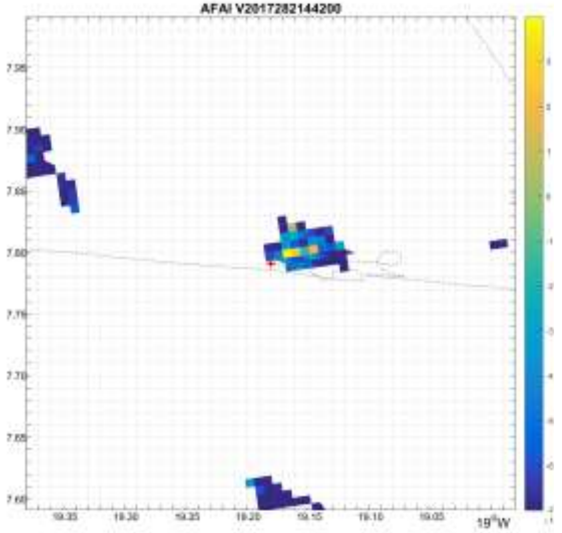

OLCI (300m) - 1 day ***MCI***

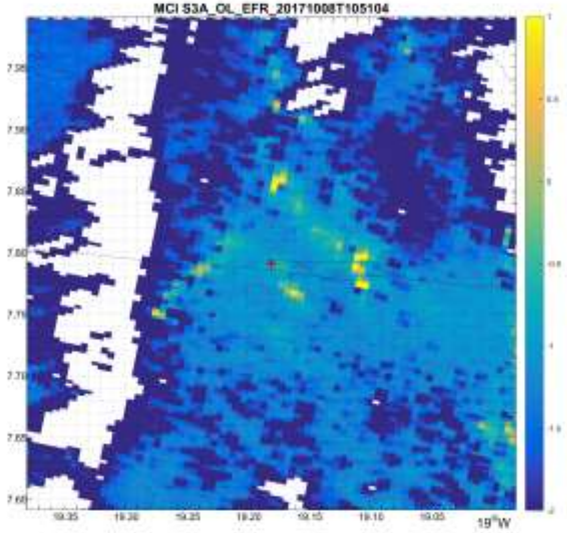

S2 Fig – 27. TRANSATLANTIC - Y04 - 2017-10-10 12.00 TU - 08°18.146'N 21°34.633'W - WS = 3.1 m.s^{-1} WD = 144° SS = Slight

MODIS-A (1km)

MODIS-T (1km)

***In situ* - Type 3**

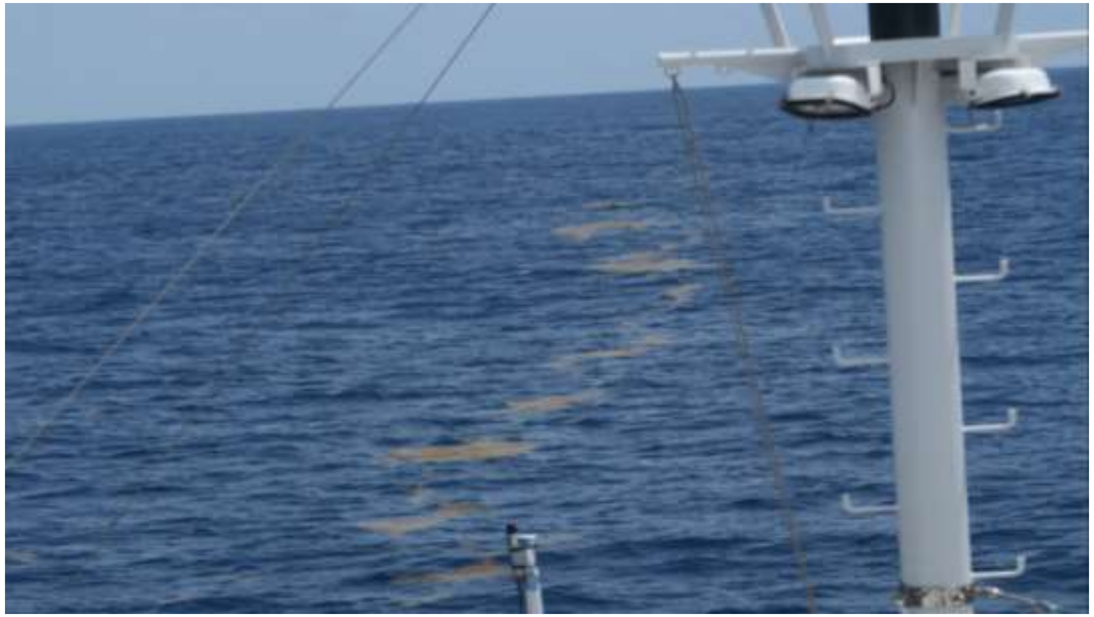

VIIRS (750 m)

AFAI

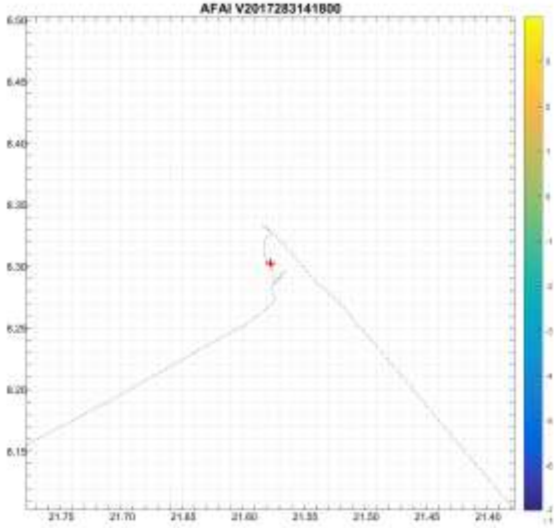

OLCI (300m)

MCI

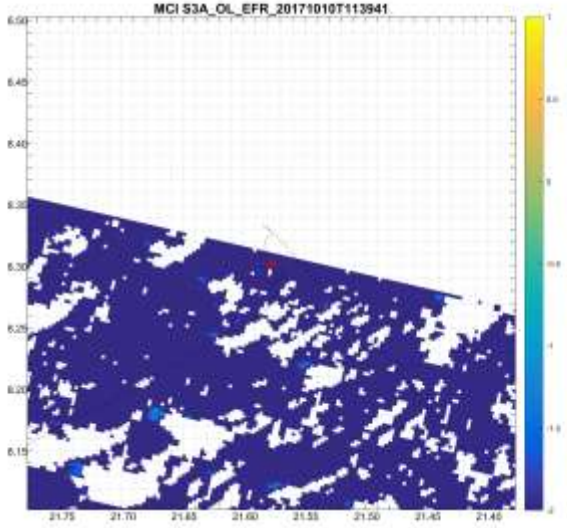

S2 Fig – 28. TRANSATLANTIC - Y04b - 2017-10-10 18.00 TU - 07°58.917'N 21°59.113'W - WS = 3.1 m.s^{-1} WD = 141° SS = Slight

MODIS-A (1km)

MODIS-T (1km)

VIIRS (750 m) **AFAI**

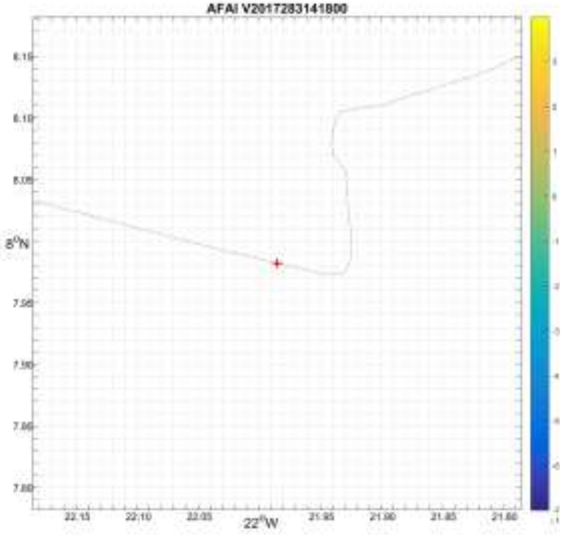

OLCI (300m) **MCI**

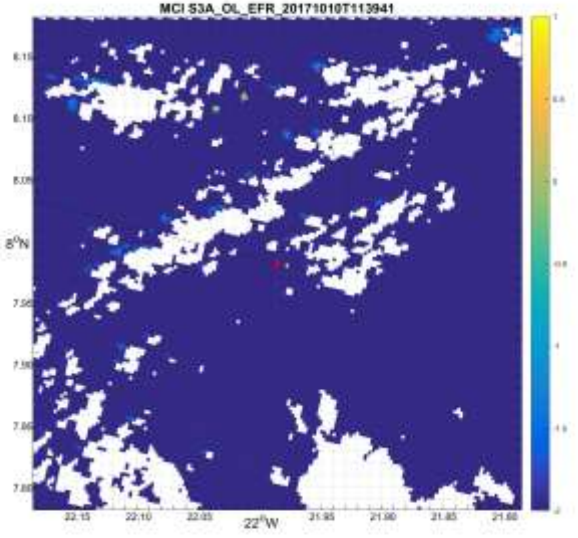

In situ - Type 5

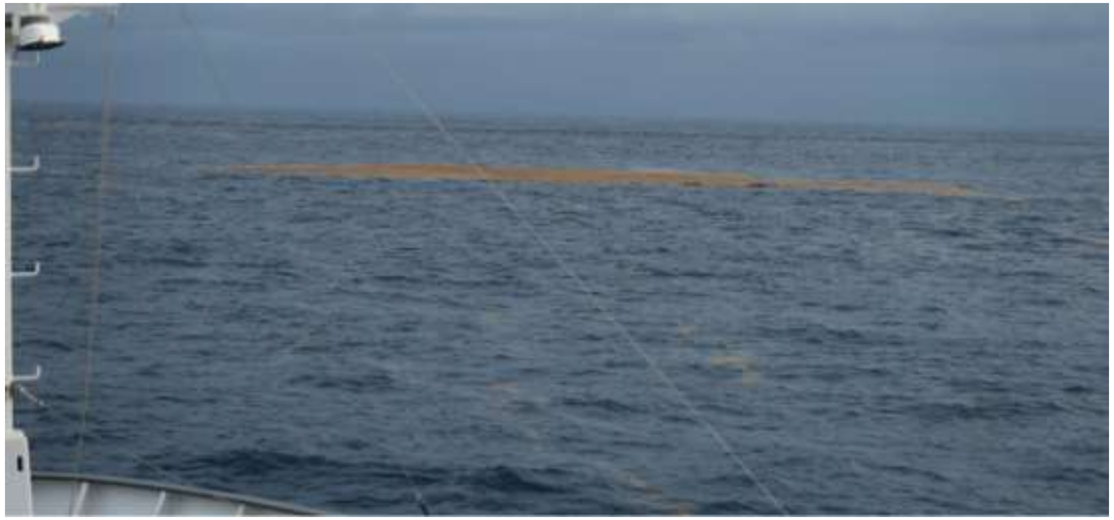

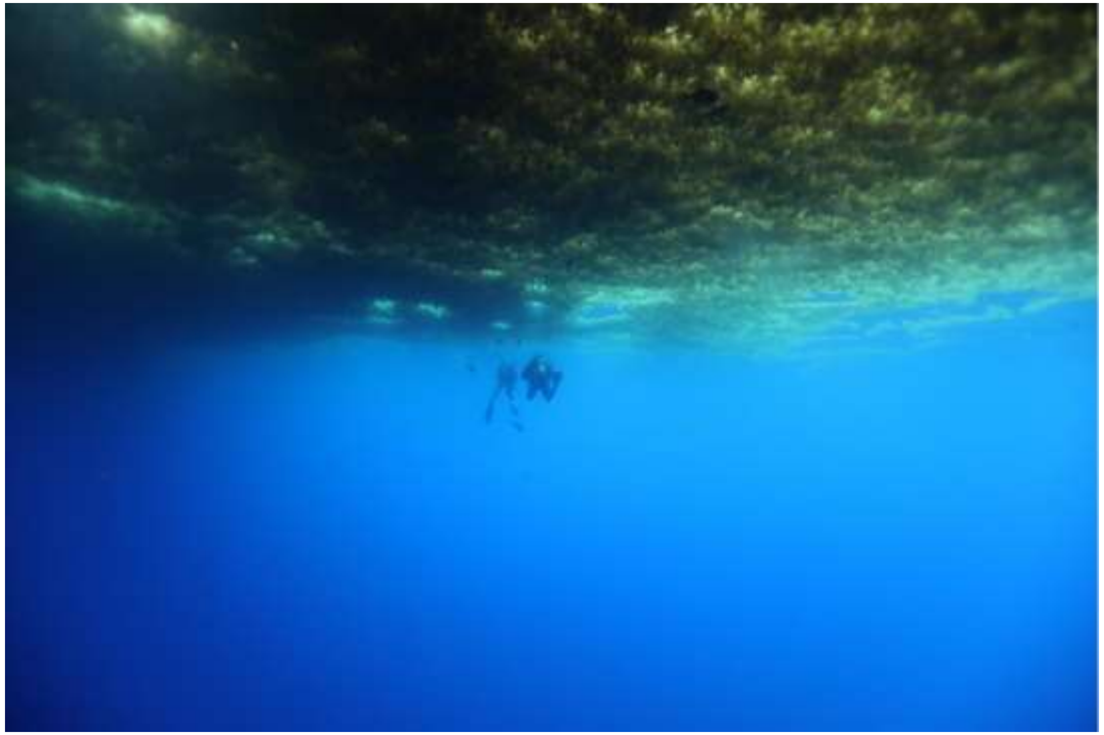

S2 Fig – 29. TRANSATLANTIC - Y05 - 2017-10-11 10.00 TU - 08°23.260'N 25°07.000'W - WS = 4.2 m.s^{-1} WD = 222° SS = Slight

MODIS-A (1km) **AFAI**

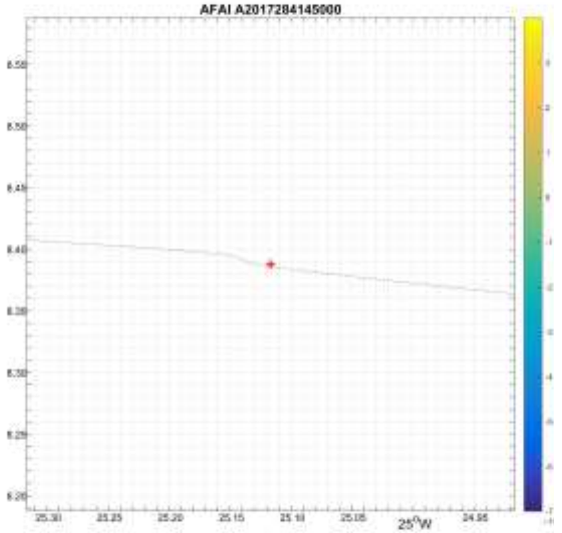

MODIS-T (1km) **AFAI**

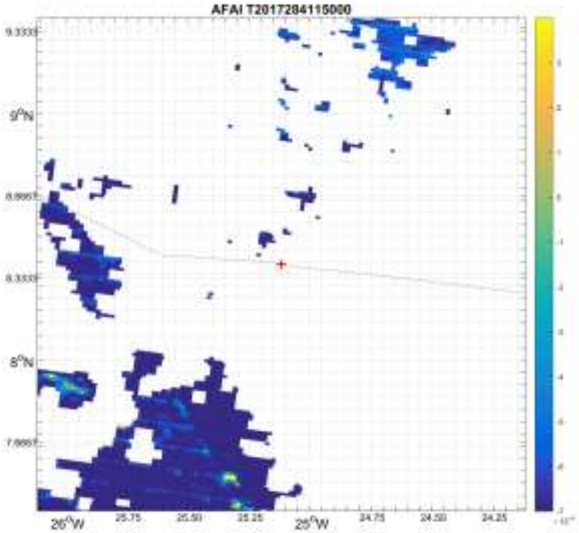

VIIRS (750 m) **AFAI**

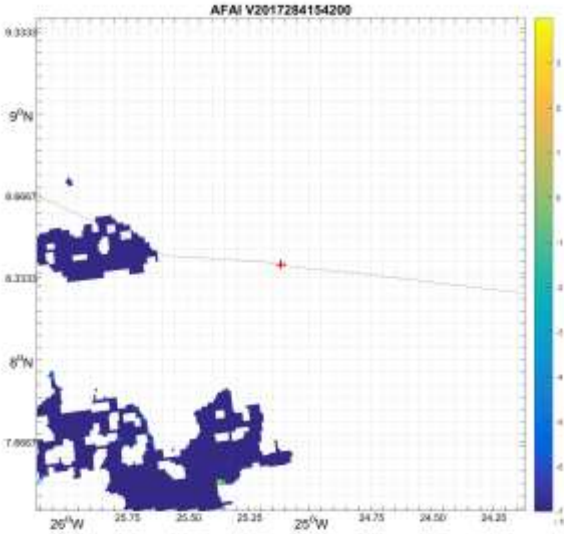

OLCI (300m) - 1 day **MCI**

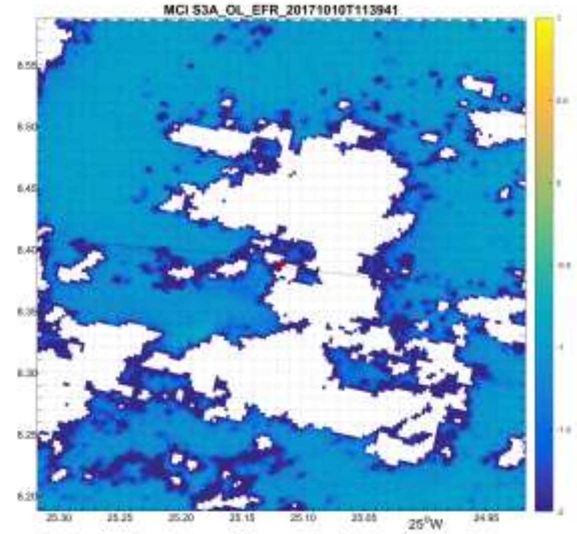

In situ - Type 1

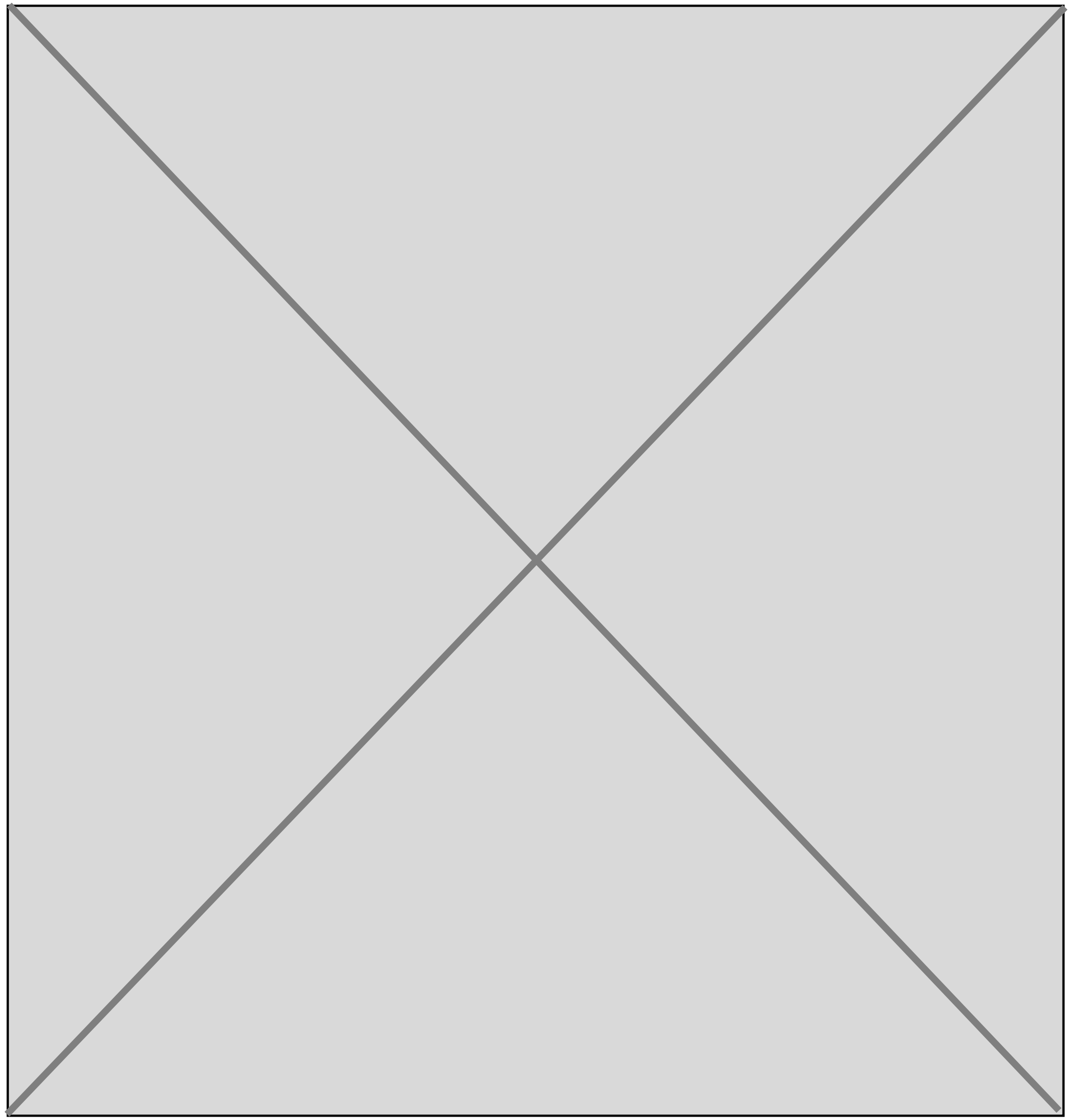

S2 Fig – 30. TRANSATLANTIC - Y06 - 2017-10-12 12.00 TU - 10°25.792'N 29°02.017'W - WS = 6.9 m.s^{-1} WD = 248° SS = Slight

MODIS-A (1km) **AFAI**

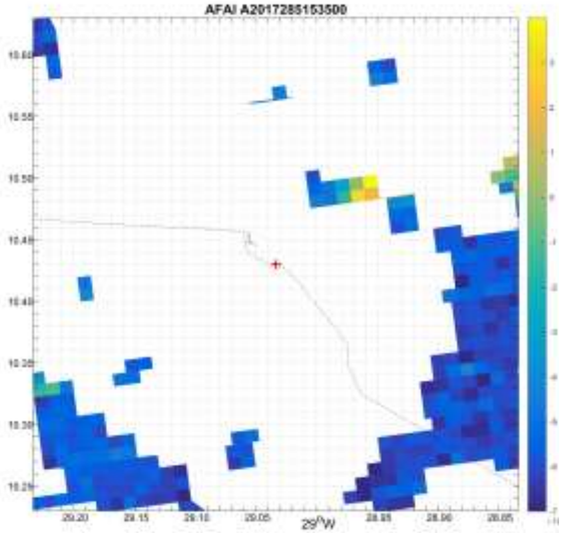

MODIS-T (1km) **AFAI**

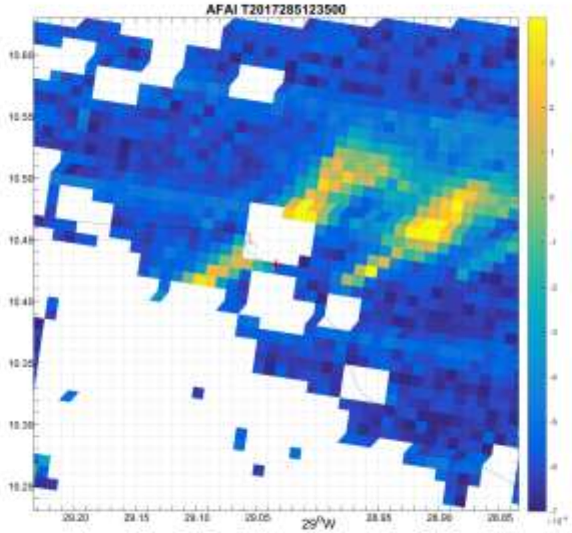

In situ - Type 4

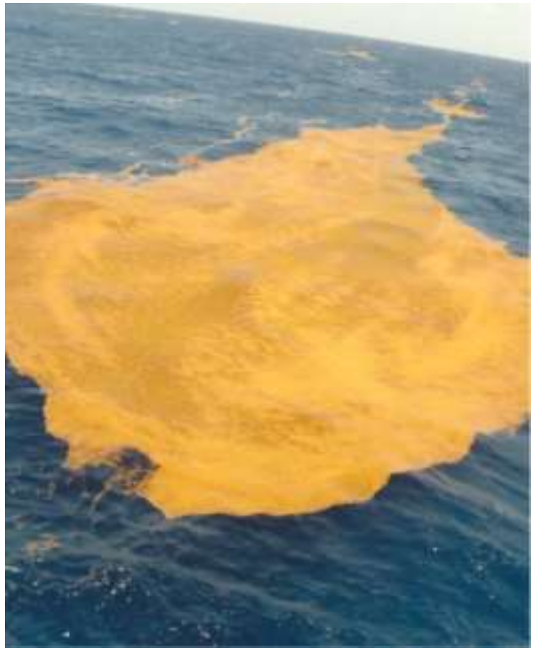

VIIRS (750 m) **AFAI**

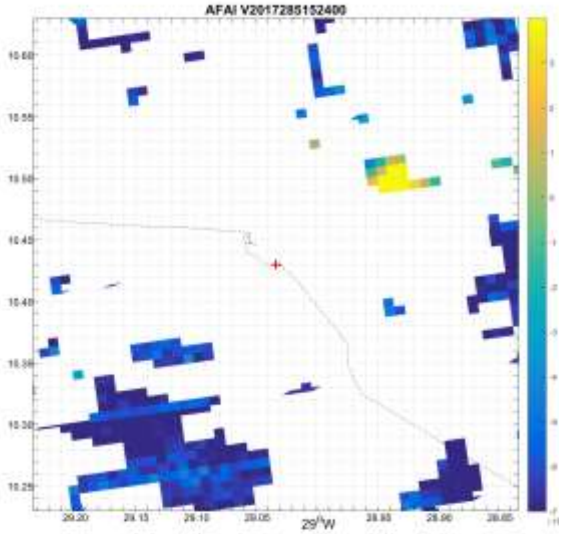

OLCI (300m) + 1 day **MCI**

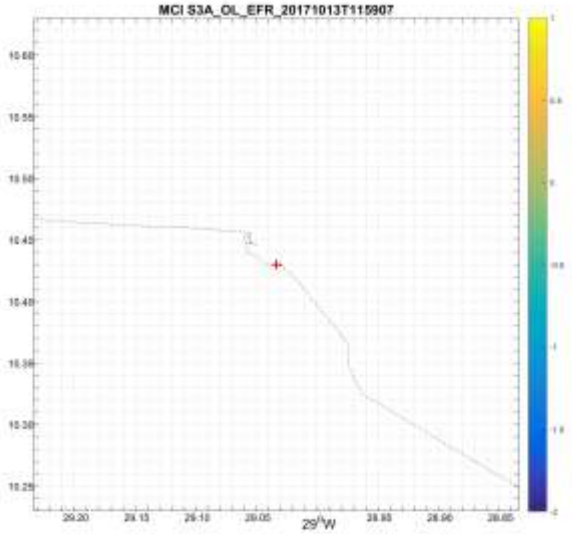

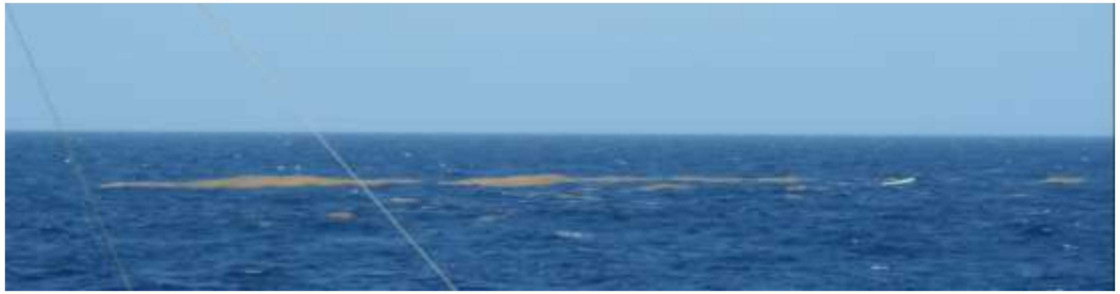

S2 Fig – 31. TRANSATLANTIC - Y07 - 2017-10-13 10.00 TU - 11°04.908'N 33°06.668'W - WS = 7.3 m.s^{-1} WD = 267° SS = Slight

MODIS-A (1km) **AFAI**

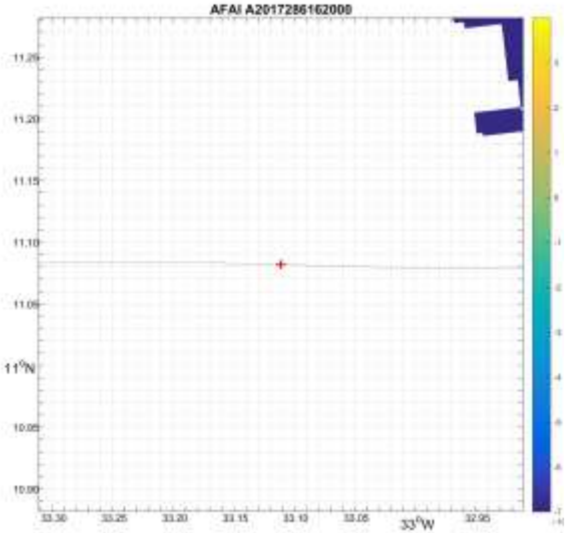

MODIS-T (1km) **AFAI**

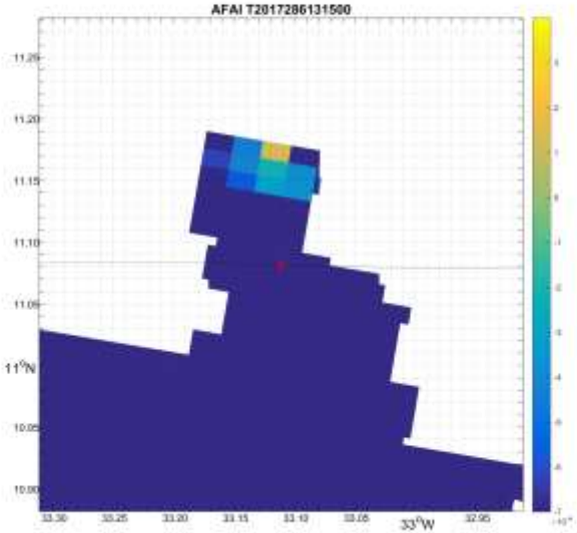

VIIRS (750 m) **AFAI**

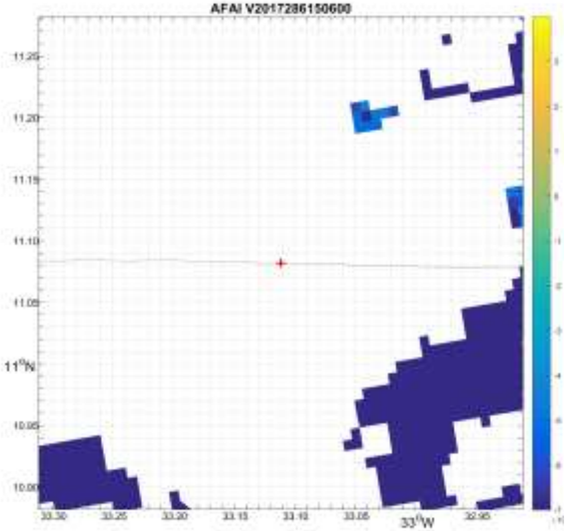

OLCI (300m) **MCI**

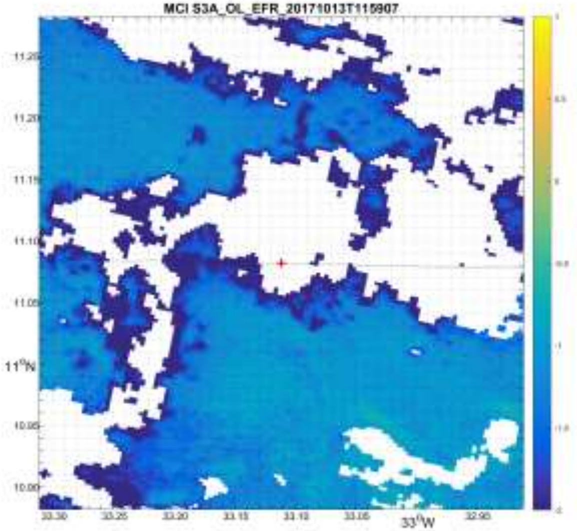

In situ - Type 2

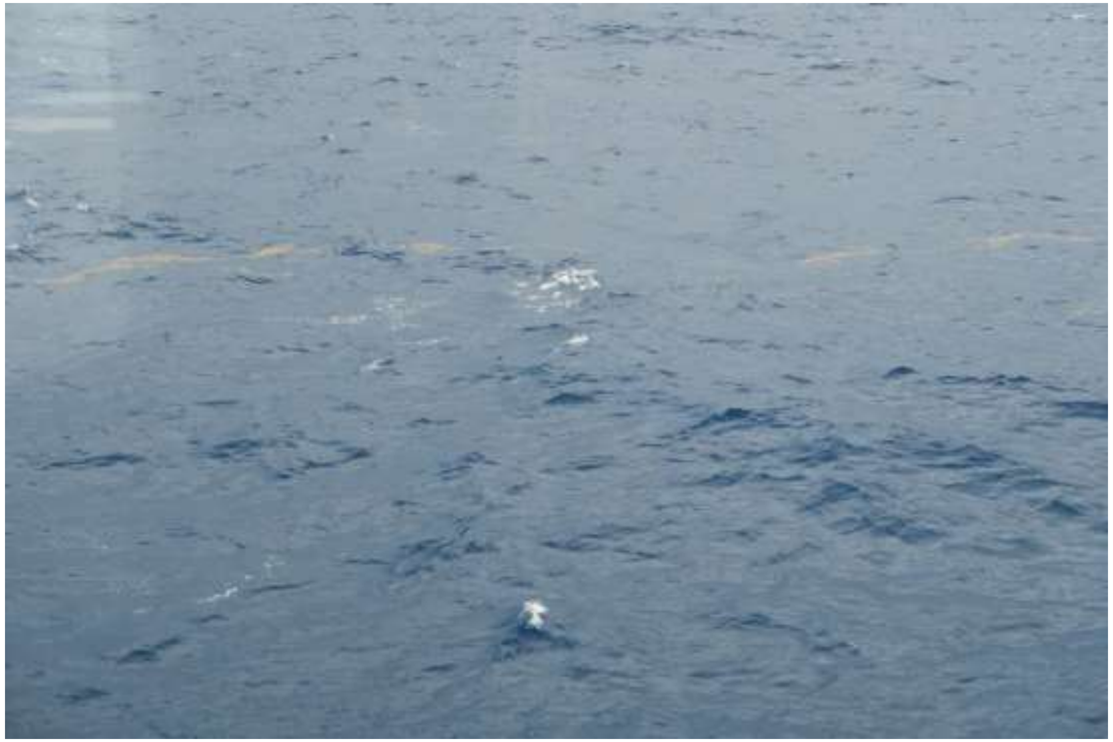

S2 Fig – 32. TRANSATLANTIC - Y07b - 2017-10-13 19.30 TU - 11°04.036'N 33°54.808'W - WS = 7.4 m.s^{-1} WD = 266°

MODIS-A (1km) **AFAI**

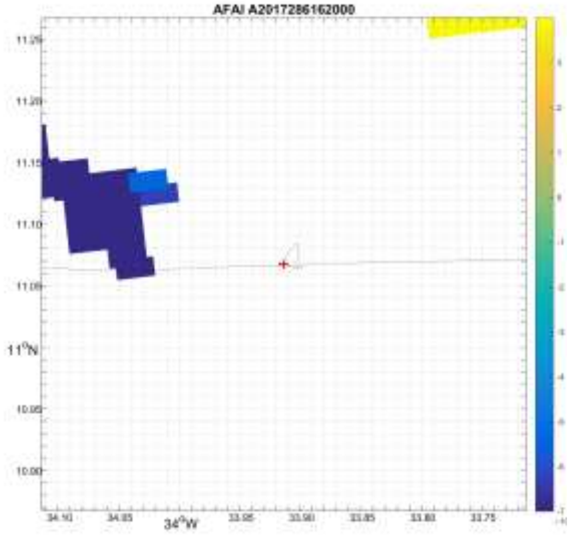

MODIS-T (1km) **AFAI**

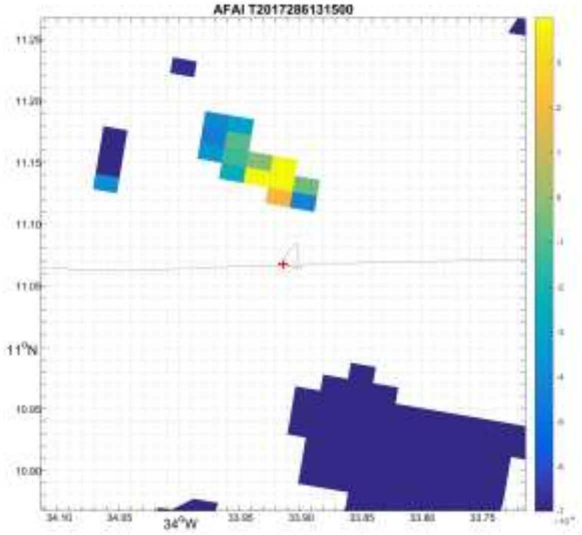

VIIRS (750 m) **AFAI**

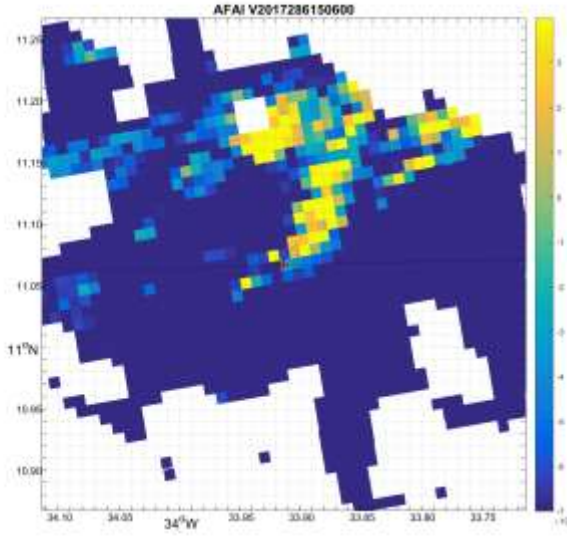

OLCI (300m) **MCI**

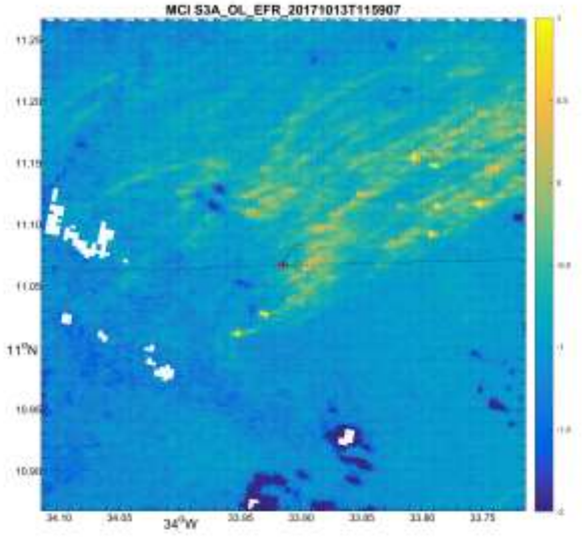

In situ - Type 5 (2 rafts)

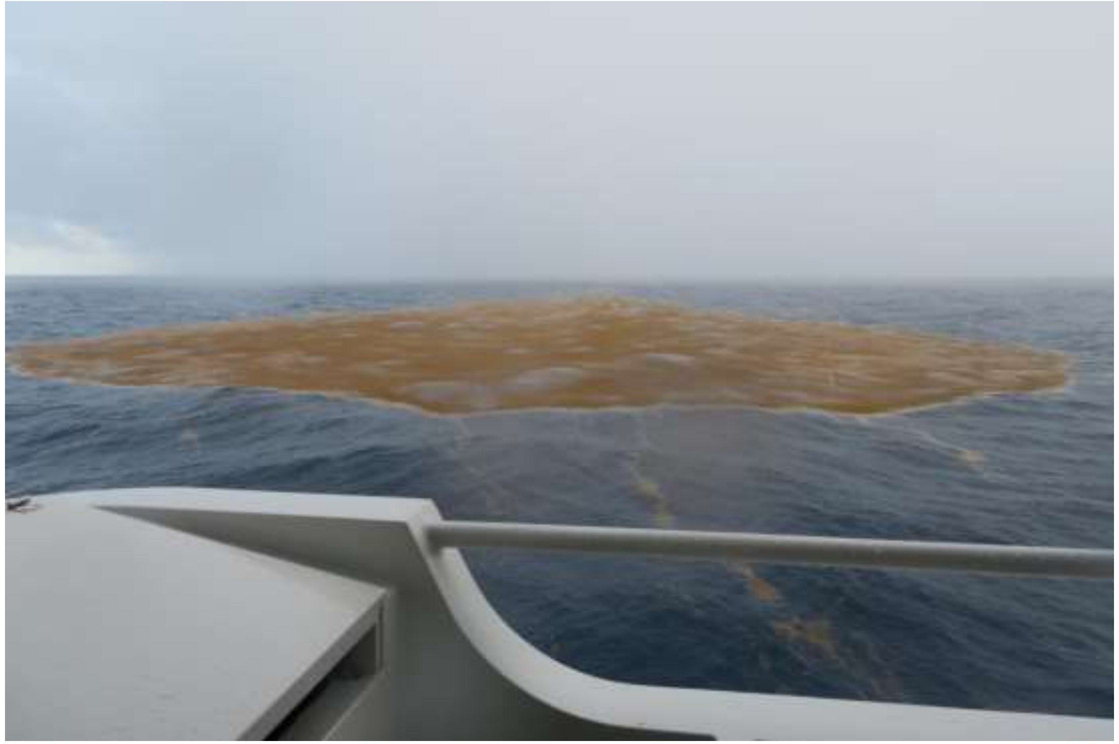

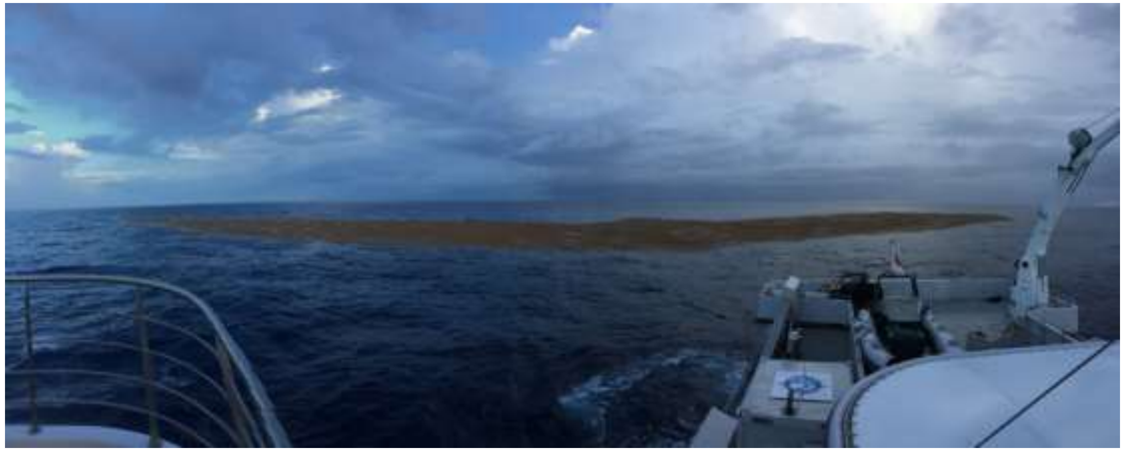

S2 Fig – 33. TRANSATLANTIC - Y08 - 2017-10-14 11.30 TU - 11°13.086'N 35°59.970'W - WS = 7.2 m.s^{-1} WD = 256° SS = Moderate

MODIS-A (1km) ***AFAI***

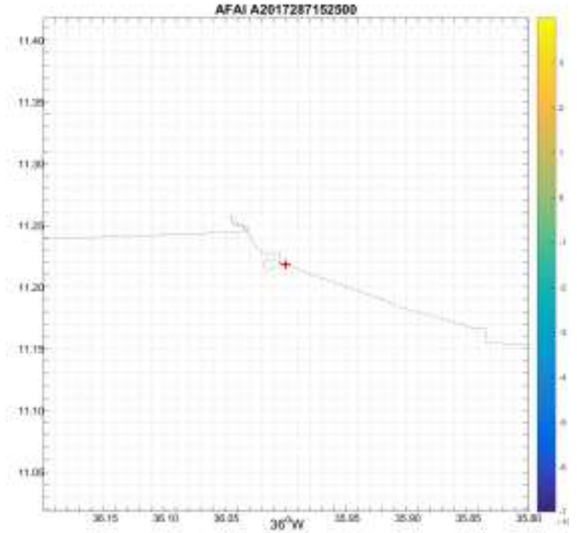

MODIS-T (1km) ***AFAI***

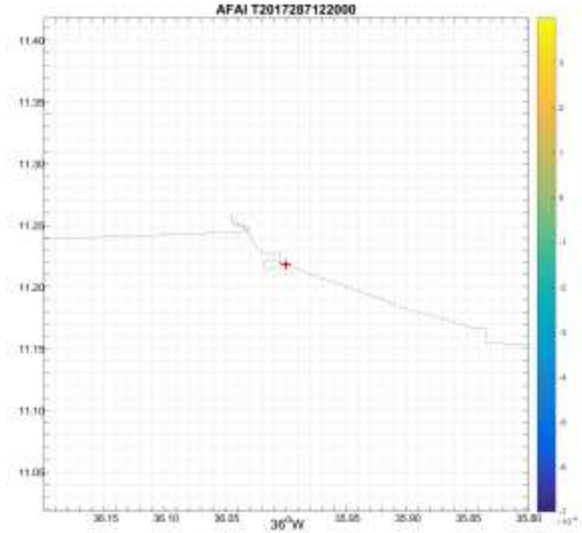

VIIRS (750 m) ***AFAI***

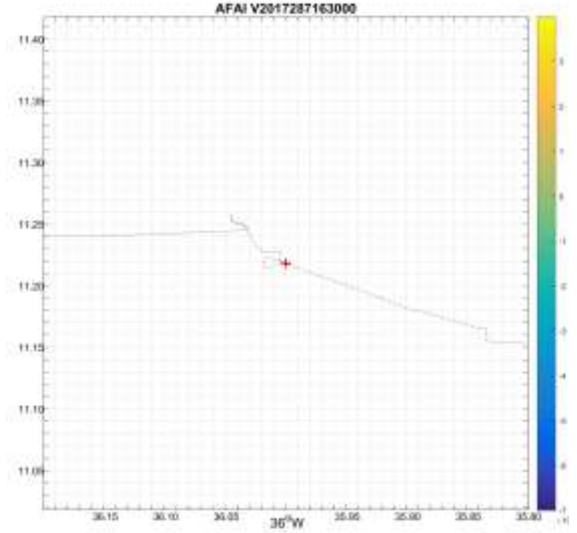

OLCI (300m) - 1 day ***MCI***

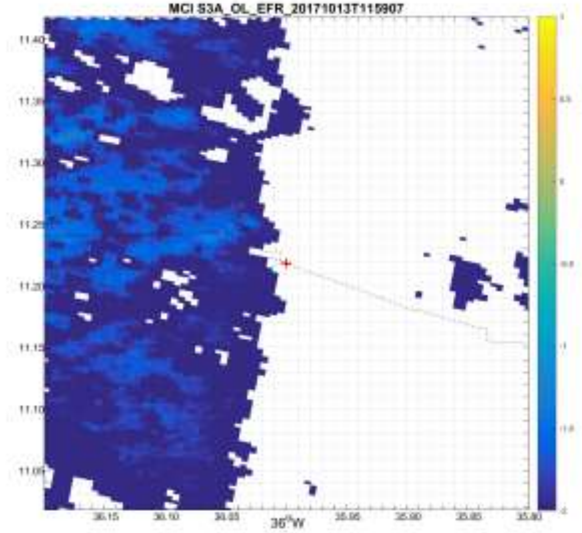

In situ - Type 2

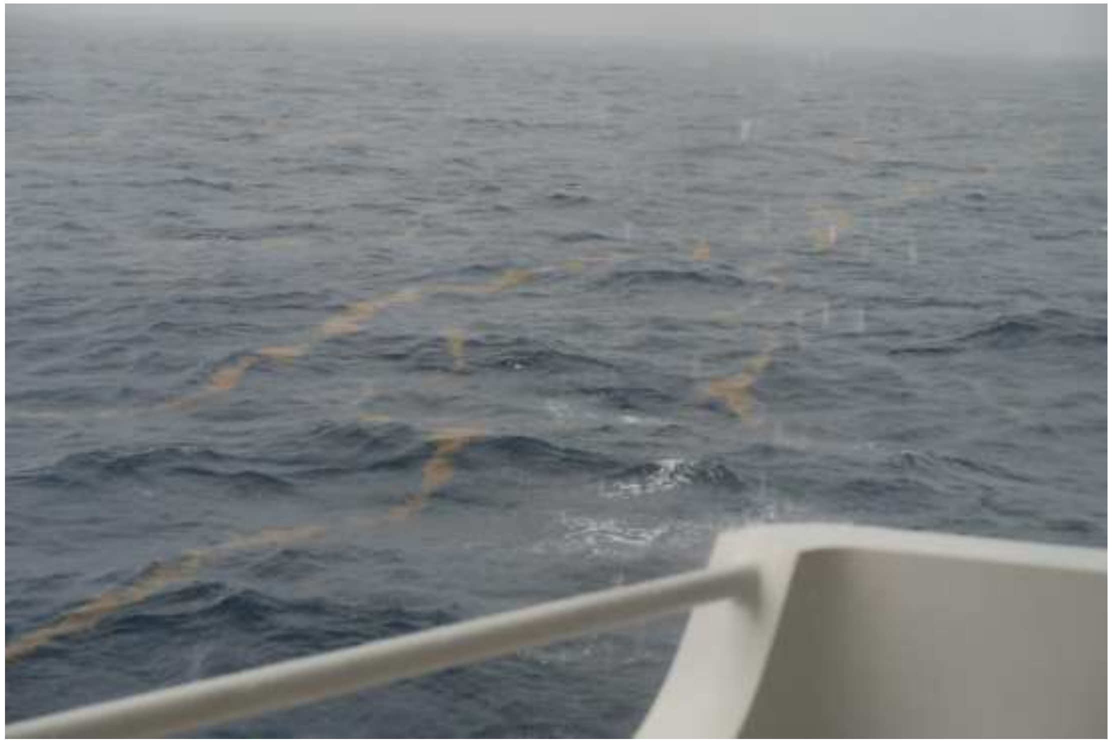

S2 Fig – 34. TRANSATLANTIC - Y09 - 2017-10-15 11.00 TU - 11°22.701'N 40°01.604'W - WS = 7.1 m.s^{-1} WD = 252° SS = Slight

MODIS-A (1km) **AFAI**

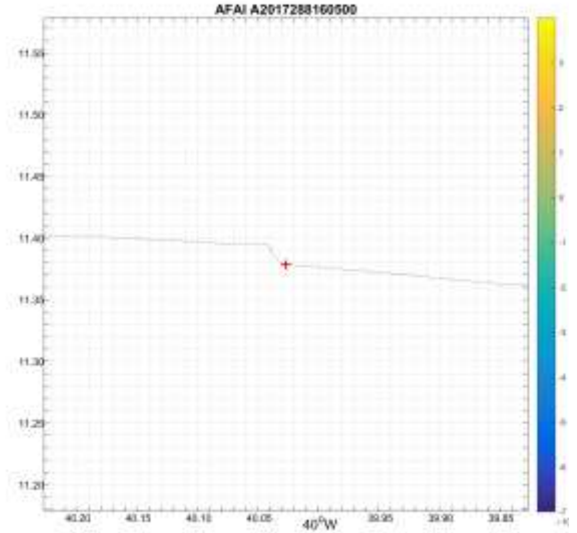

MODIS-T (1km) **AFAI**

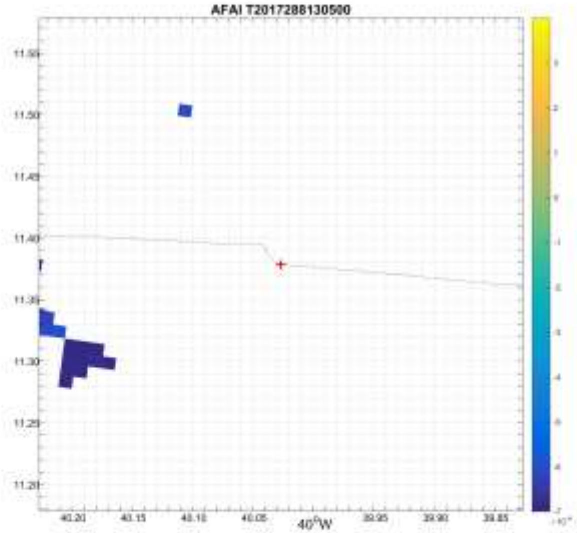

VIIRS (750 m) **AFAI**

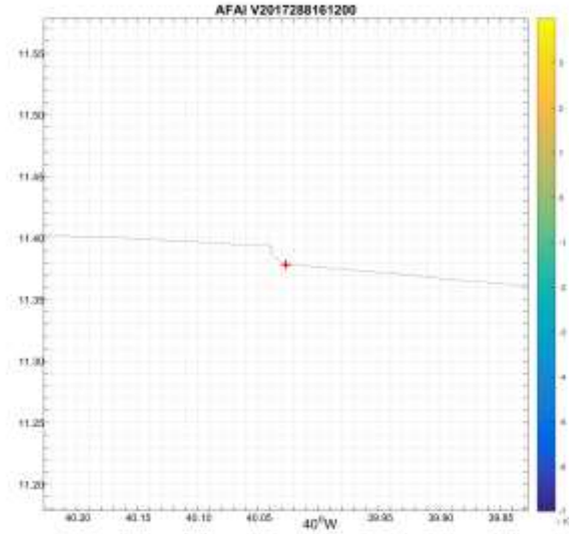

OLCI (300m) **MCI**

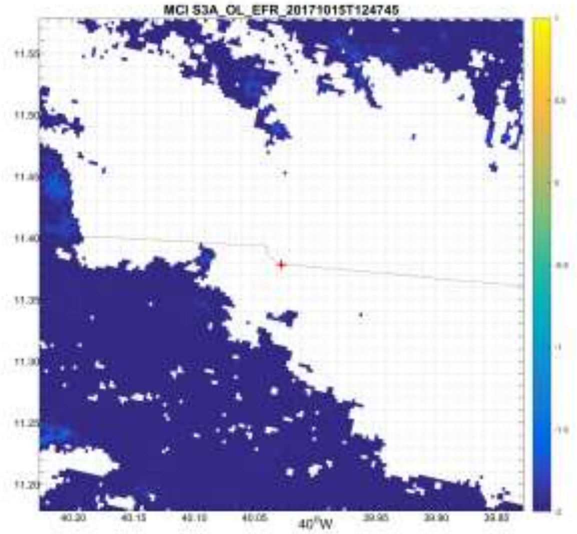

In situ - Type1

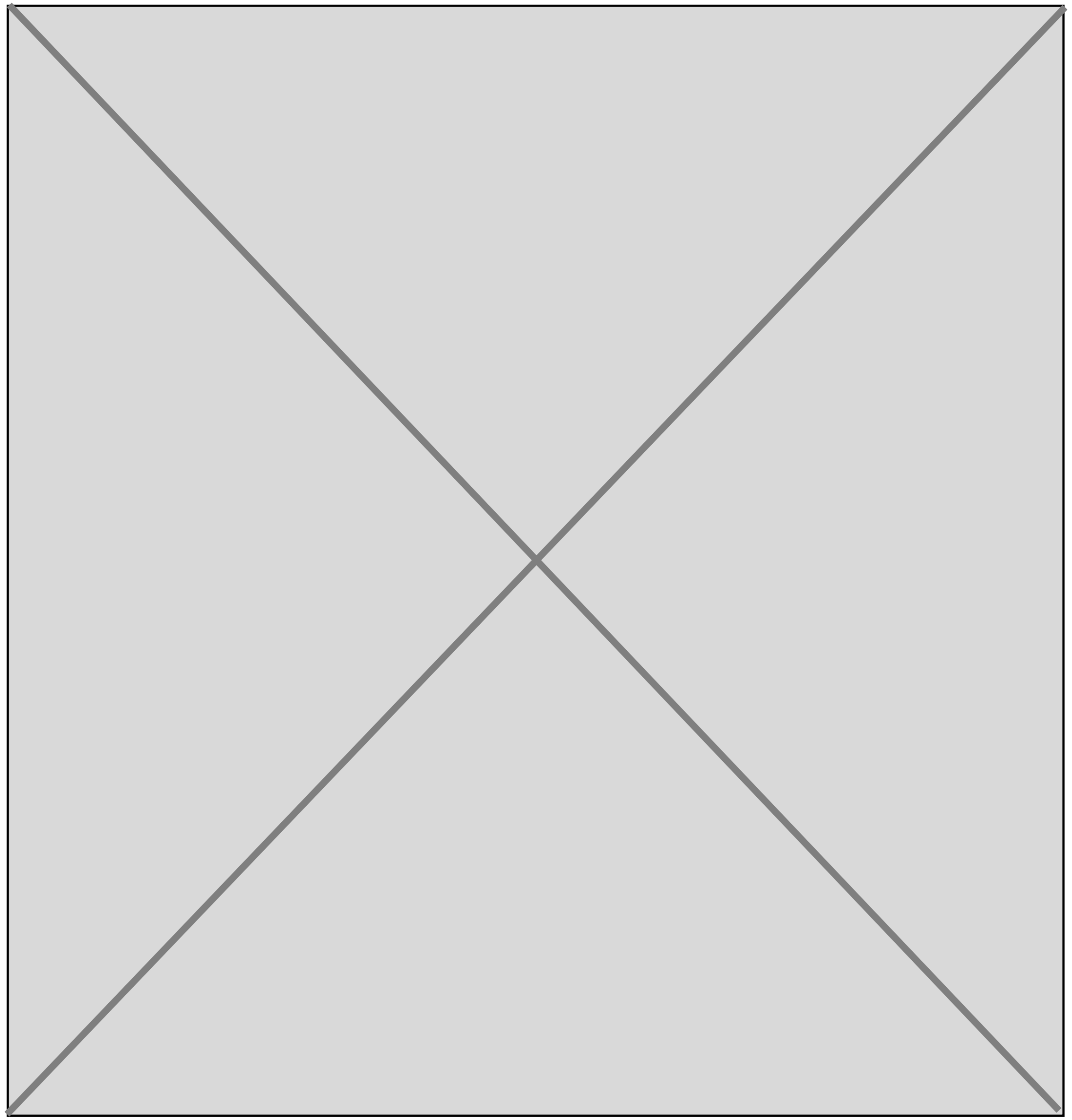

S2 Fig – 35. TRANSATLANTIC - Y10 - 2017-10-16 12.00 TU - 11°43.499'N 45°03.126'W - WS = 8.8 m.s^{-1} WD = 257° SS = Moderate

MODIS-A (1km) **AFAI**

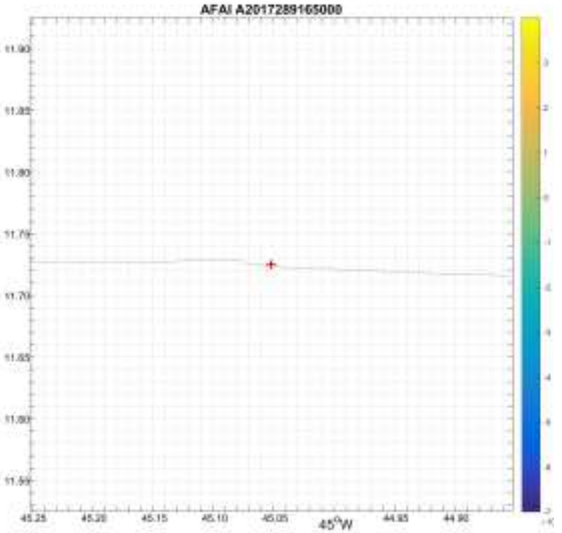

MODIS-T (1km) **AFAI**

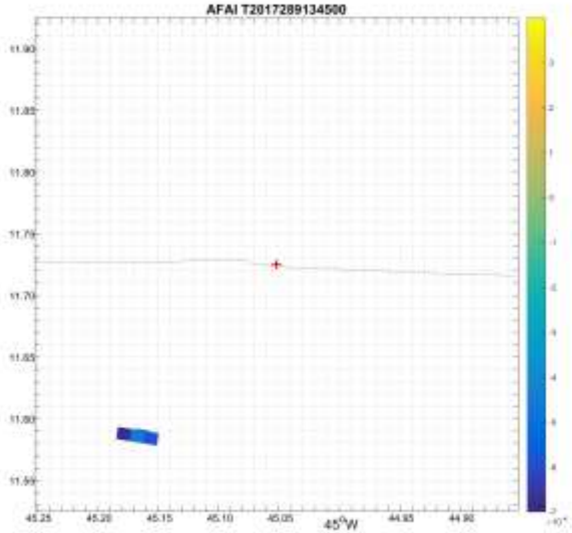

***In situ* - Absence**

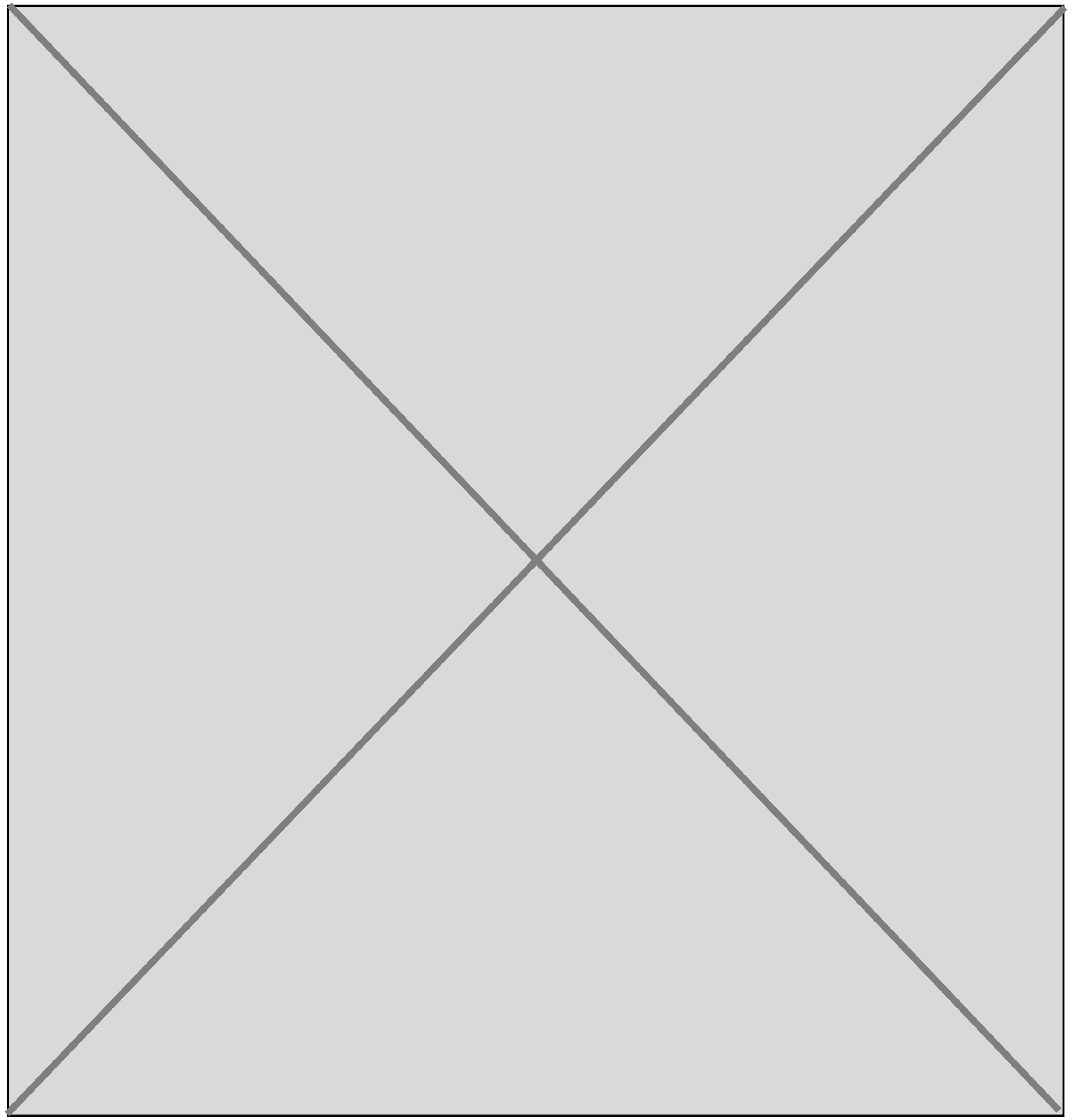

VIIRS (750 m) **AFAI**

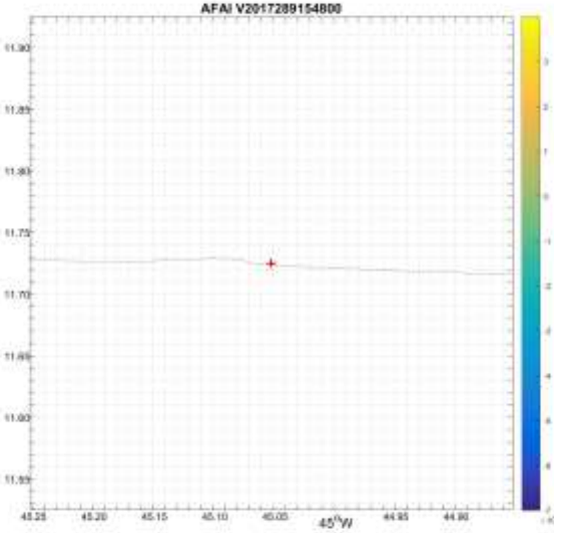

OLCI (300m) - 1 day **MCI**

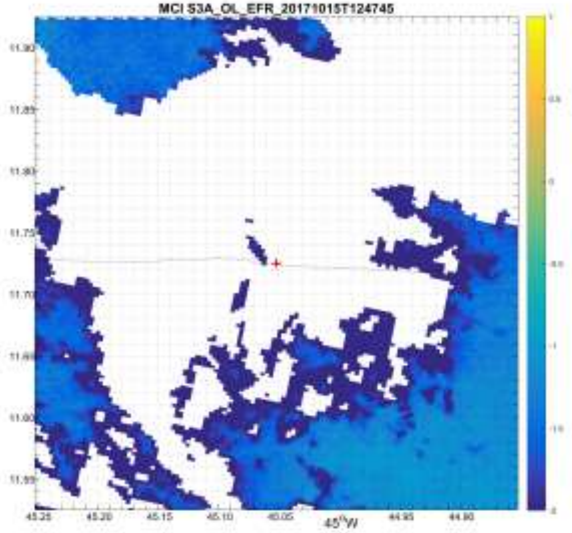

S2 Fig – 36. TRANSATLANTIC - Y11 - 2017-10-17 12.00 TU - 11°57.953'N 49°05.291'W - WS = 9.4 m.s^{-1} WD = 257° SS = Rough

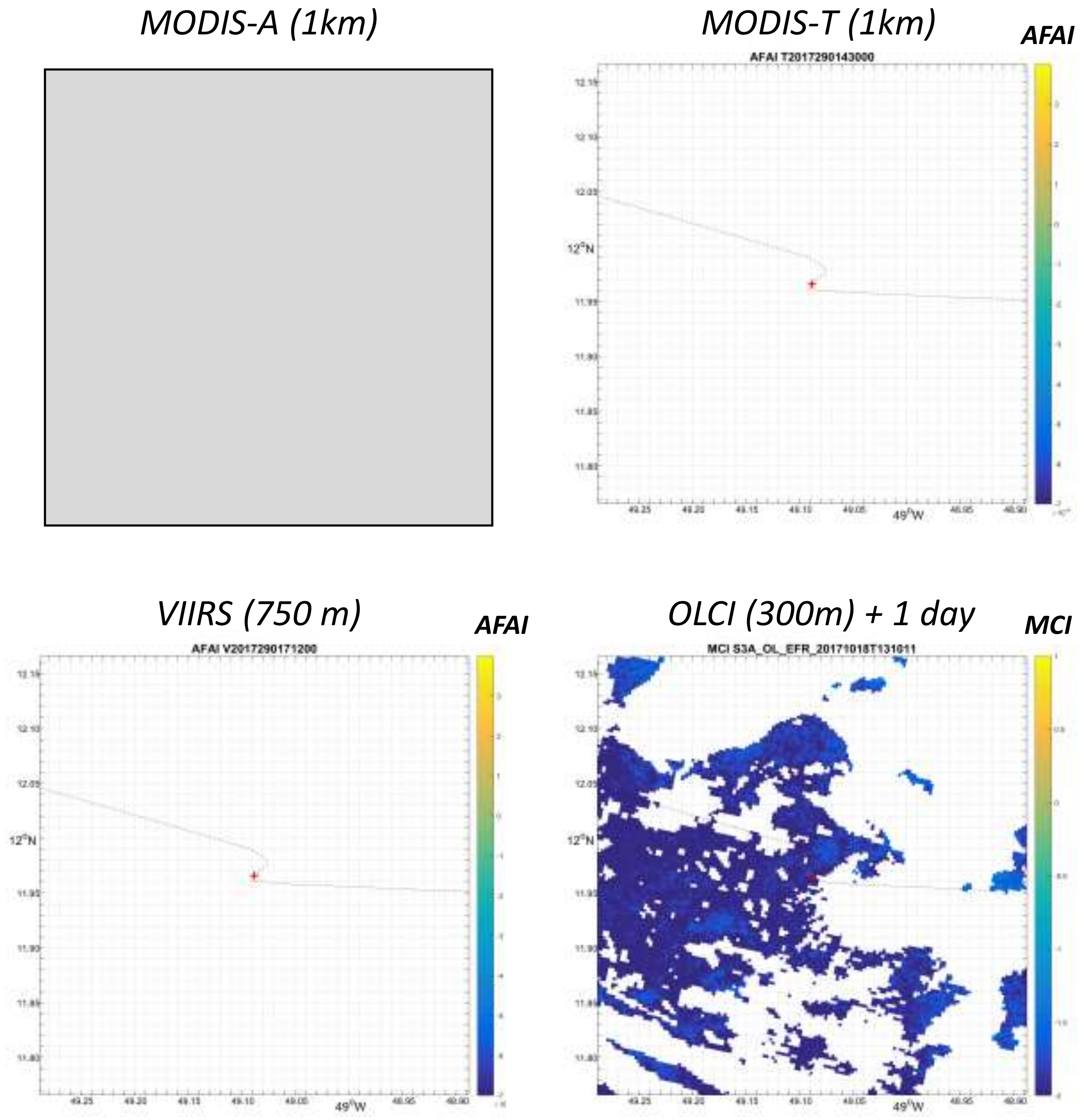

In situ - Absence

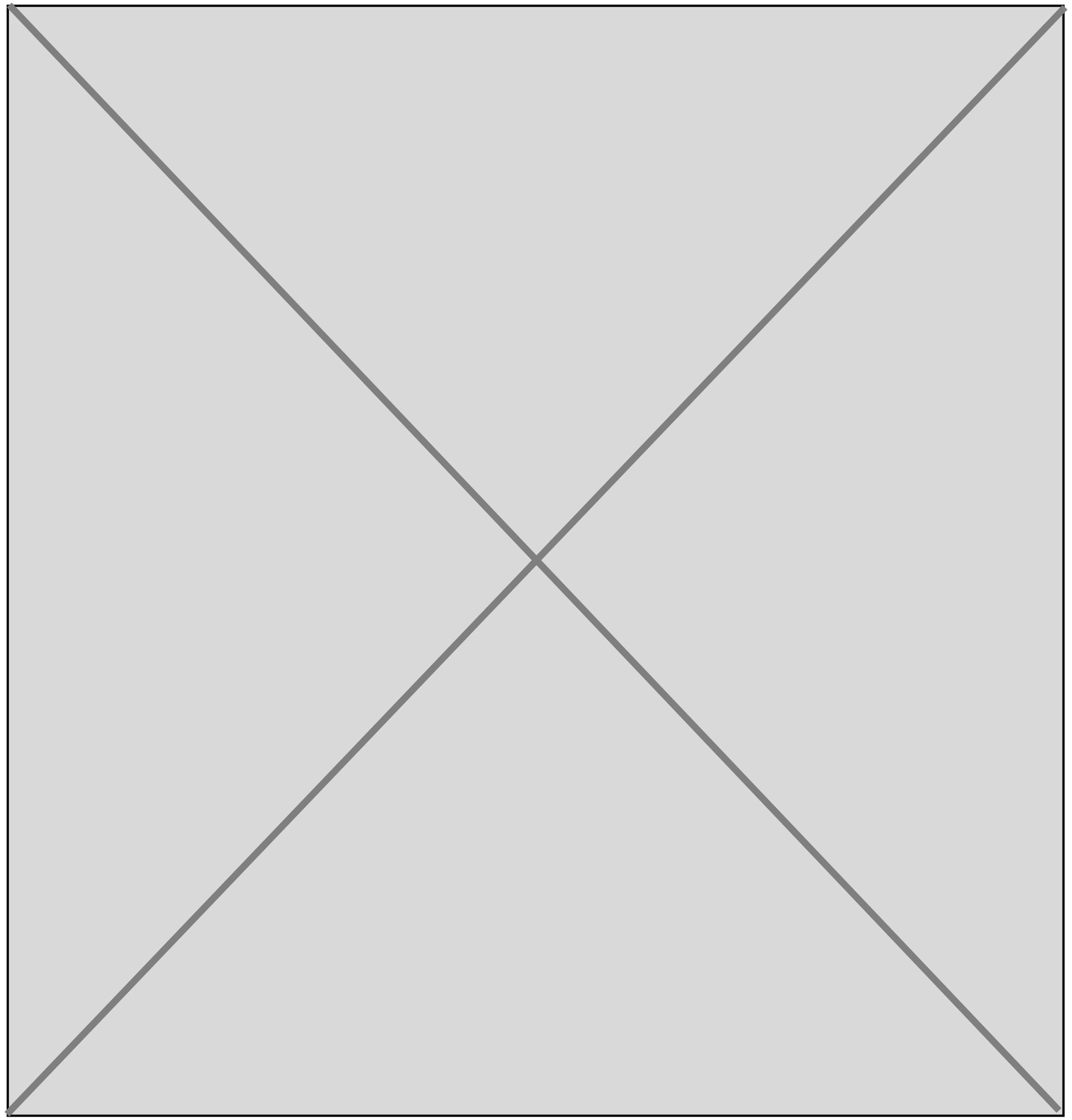

S2 Fig – 37. TRANSATLANTIC - Y11b - 2017-10-17 17.45 TU - 12°07.644'N 50°02.548'W - WS = 9.4 m.s^{-1} WD = 260° SS = Moderate

MODIS-A (1km)

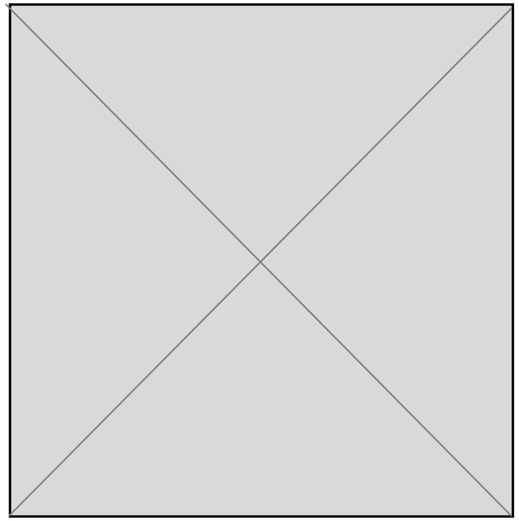

MODIS-T (1km)

AFAI

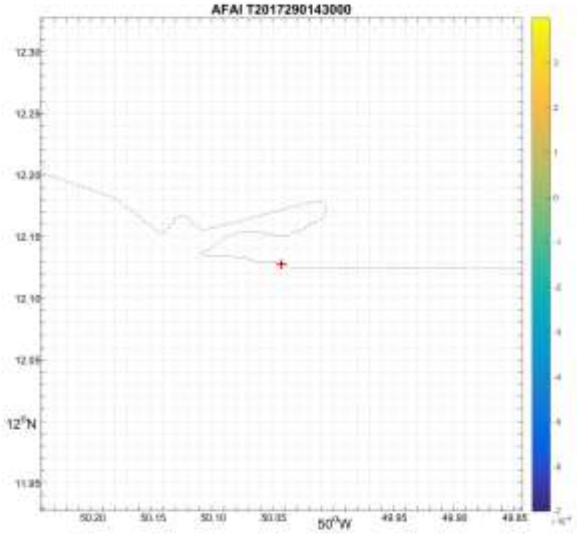

VIIRS (750 m)

AFAI

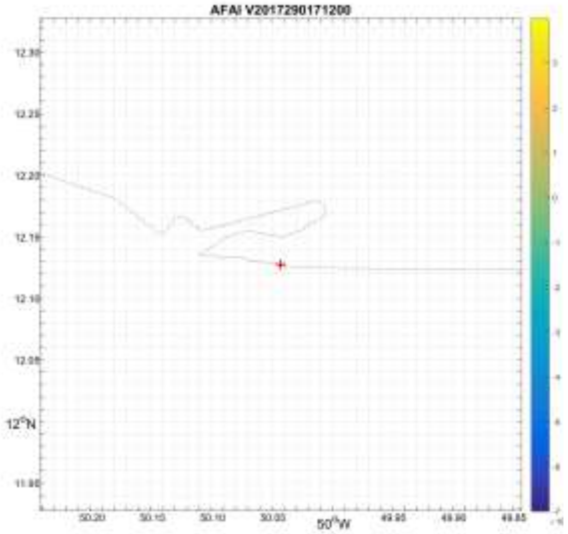

OLCI (300m) + 1 day

MCI

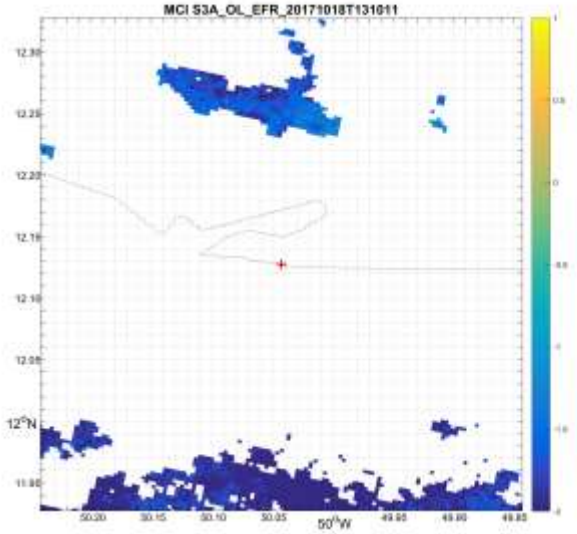

In situ - Type 2

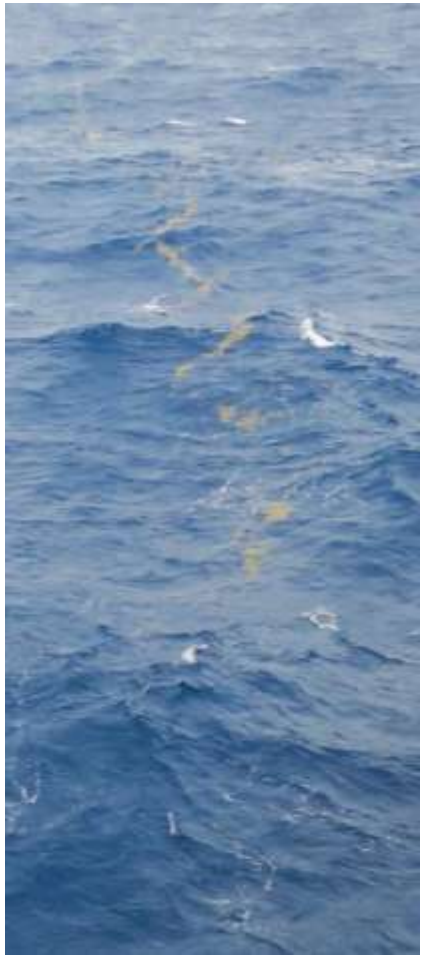

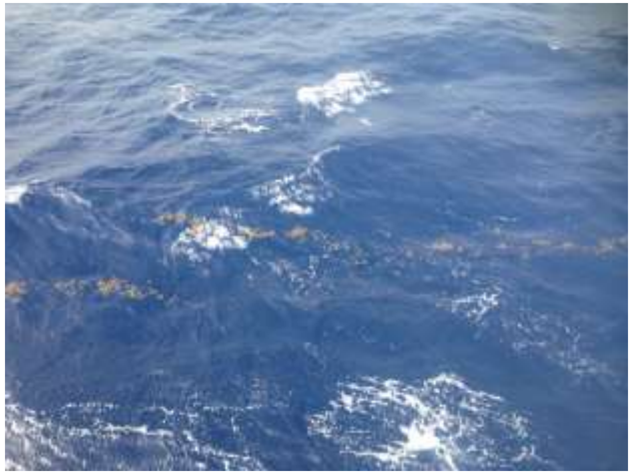

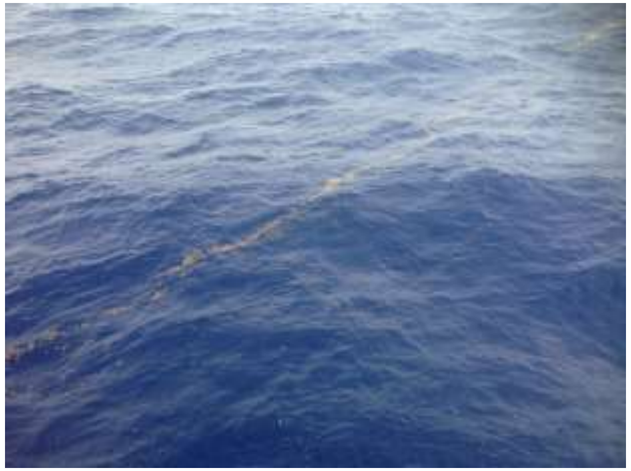

S2 Fig – 38. TRANSATLANTIC - Y12 - 2017-10-18 12.00 TU - 12°33.091'N 52°40.157'W - WS = 11m.s^{-1} WD = 263° SS = Rough

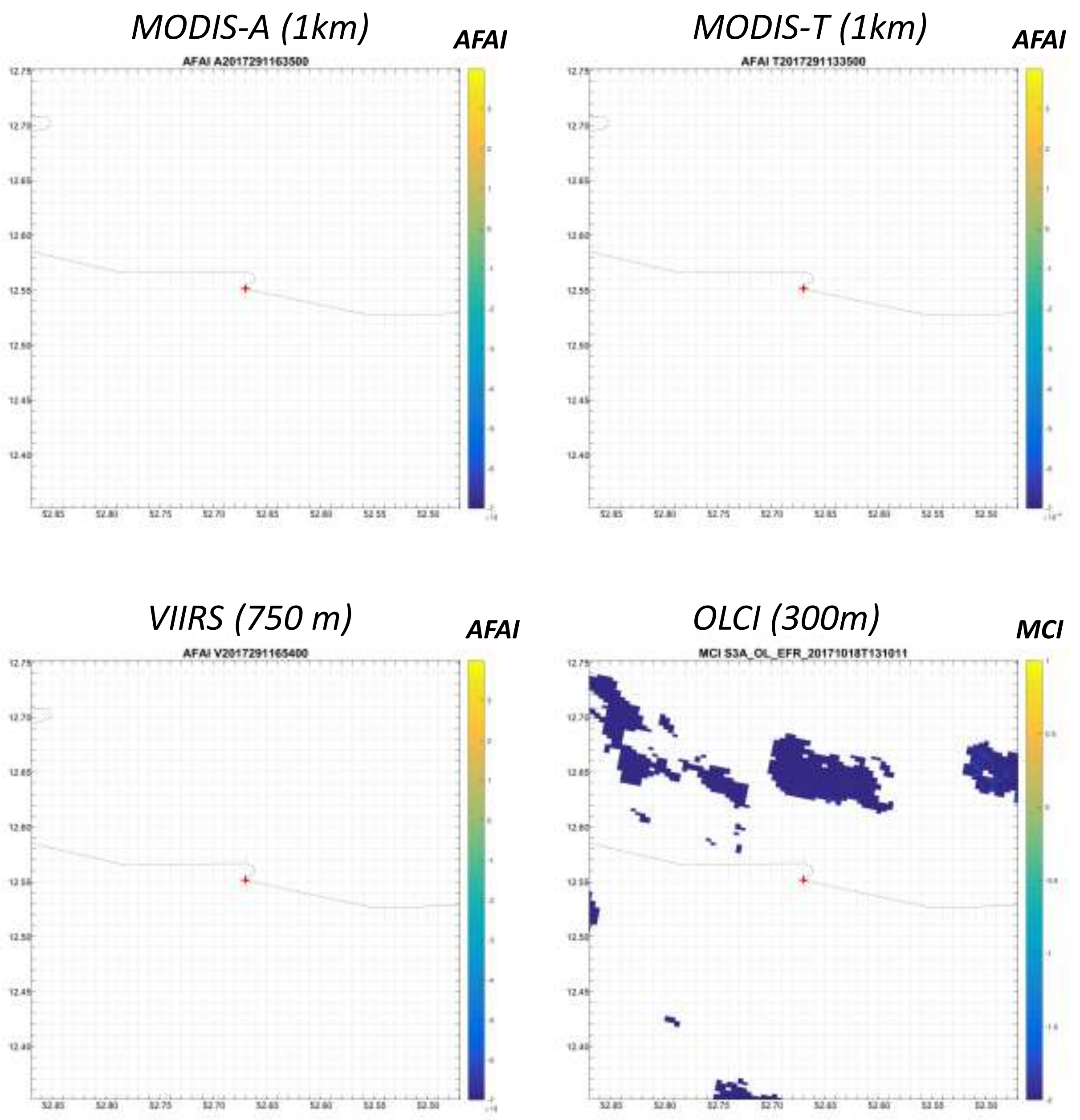

***In situ* - Type 2**

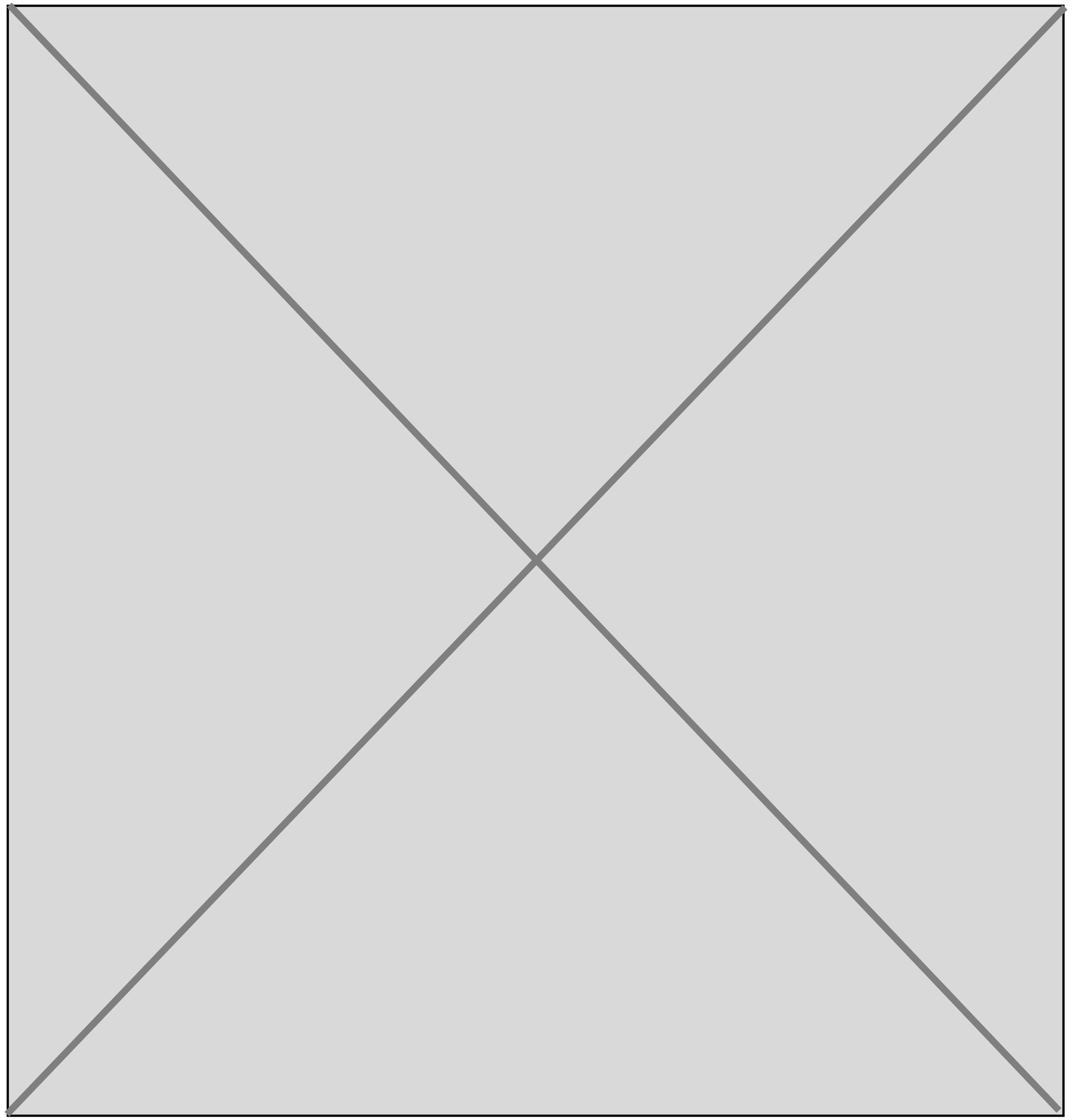

S2 Fig – 39. TRANSATLANTIC - Y12b - 2017-10-18 14.41 TU - 12°37.539'N 53°03.779'W - WS = 10.9 $m.s^{-1}$ WD = 263° SS = Rough

MODIS-A (1km) **AFAI**

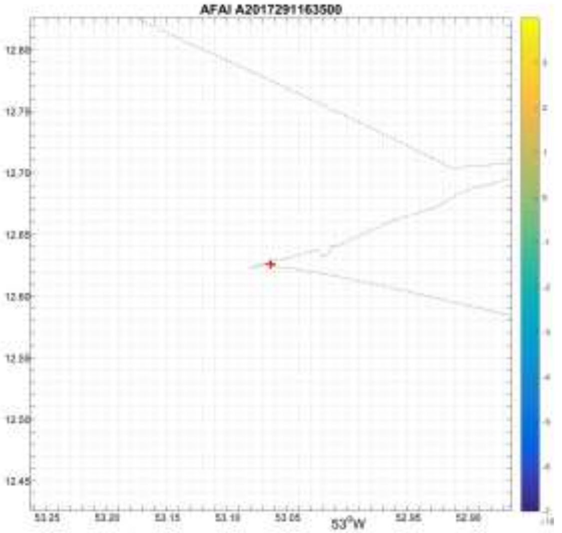

MODIS-T (1km) **AFAI**

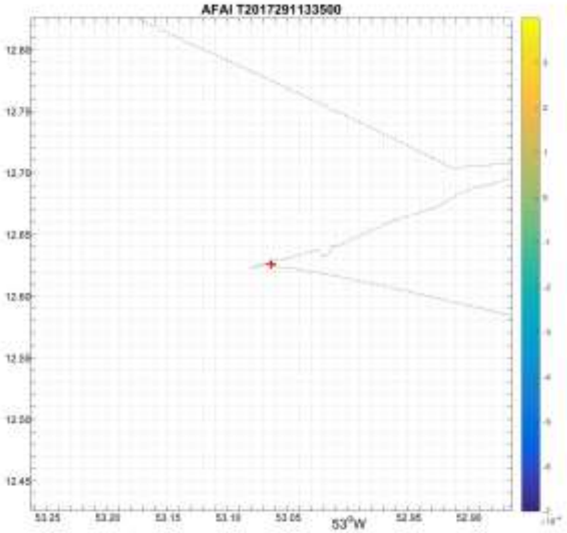

VIIRS (750 m) **AFAI**

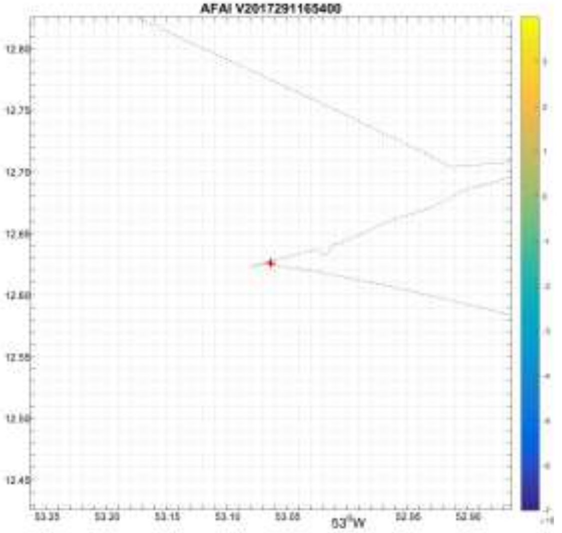

OLCI (300m) **MCI**

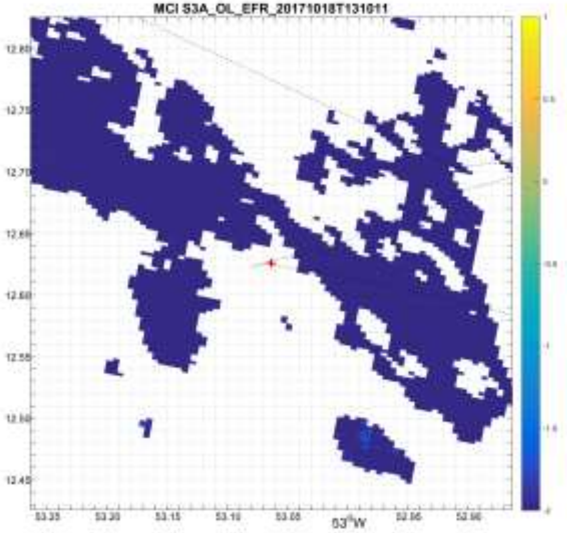

In situ - Type 3

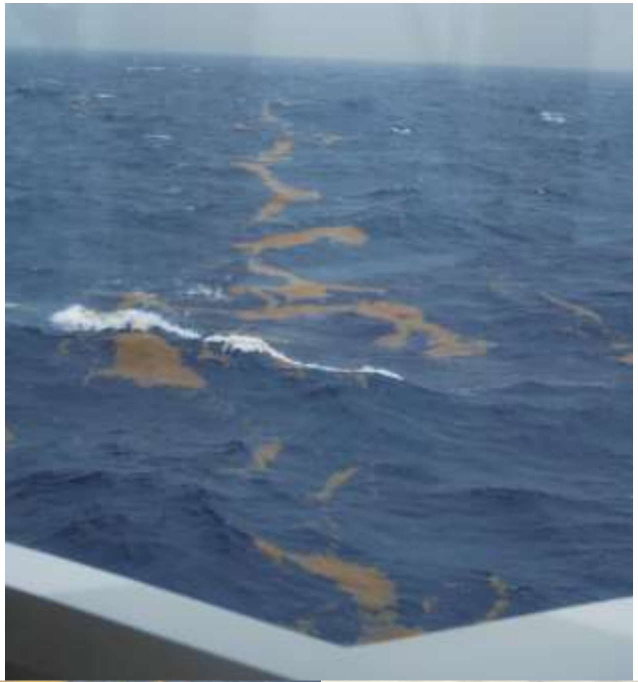

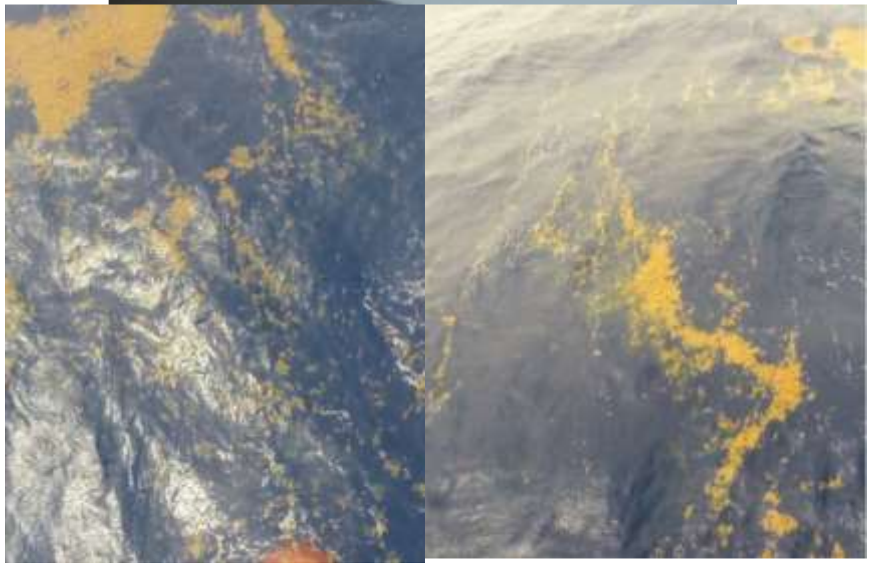

S2 Fig – 40. TRANSATLANTIC - Y13 - 2017-10-19 13.00 TU - 13°59.327'N 55°42.738'W - WS = 10 m.s^{-1} WD = 260° SS = Rough

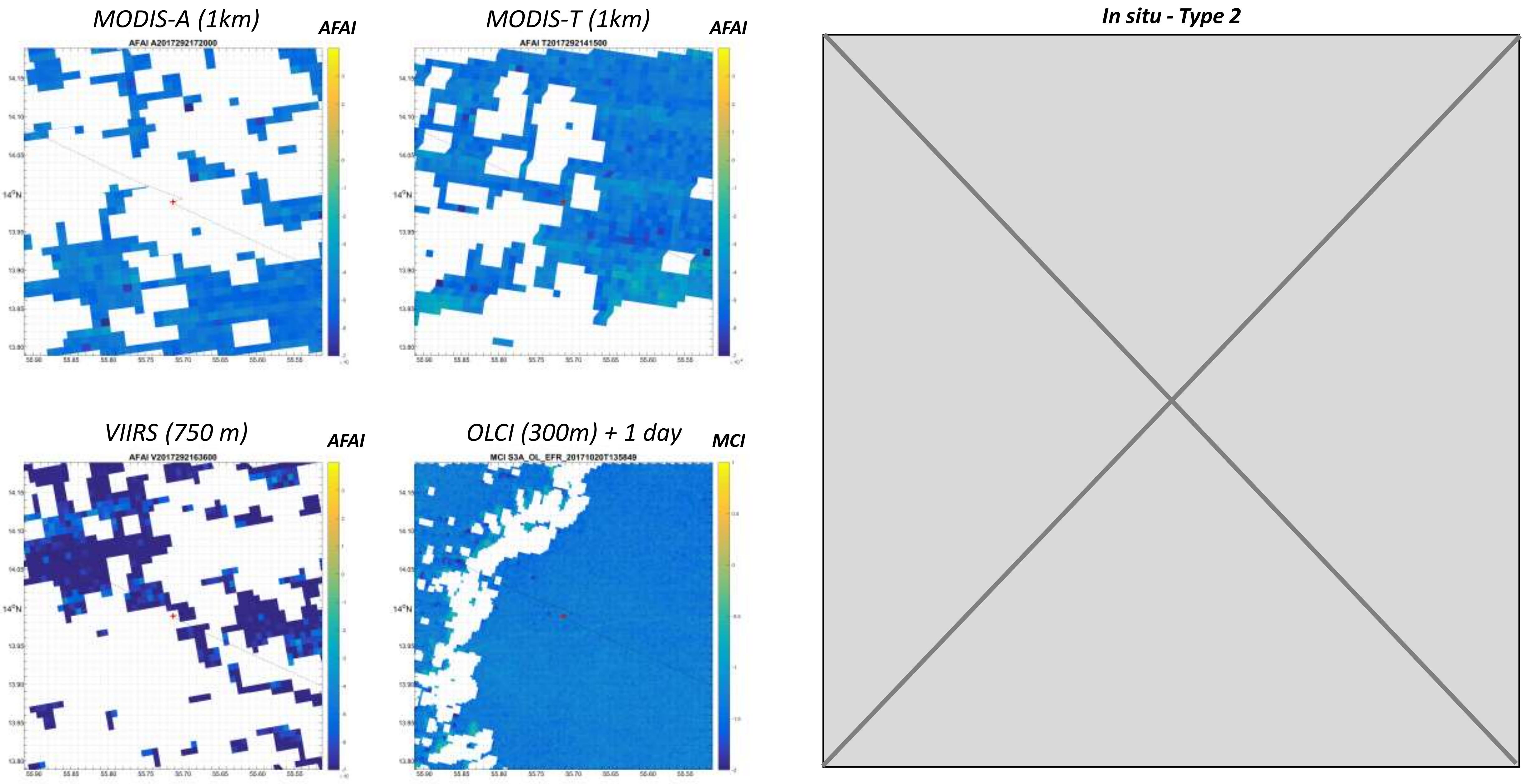

S2 Fig – 41. TRANSATLANTIC - Y14 - 2017-10-20 13.00 TU - 15°57.628'N 59°41.706'W - WS = 8.4 m.s^{-1} WD = 267° SS = Slight

MODIS-A (1km) **AFAI**

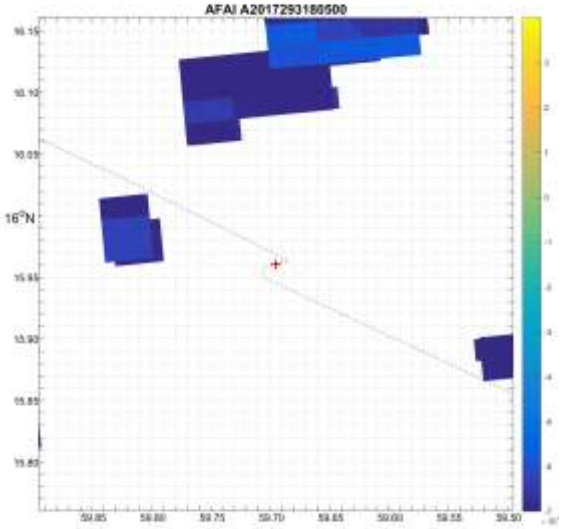

MODIS-T (1km) **AFAI**

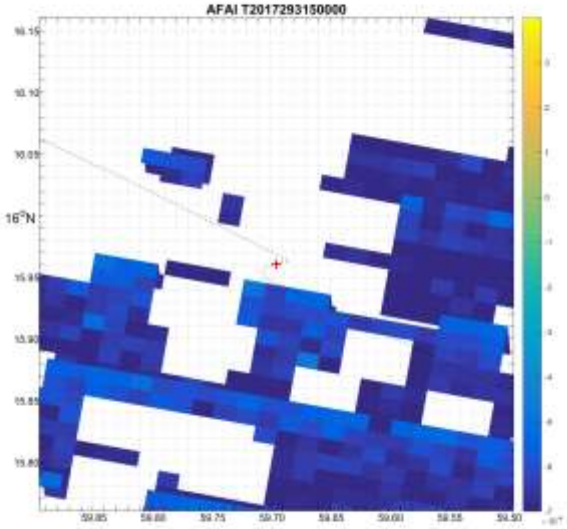

VIIRS (750 m) **AFAI**

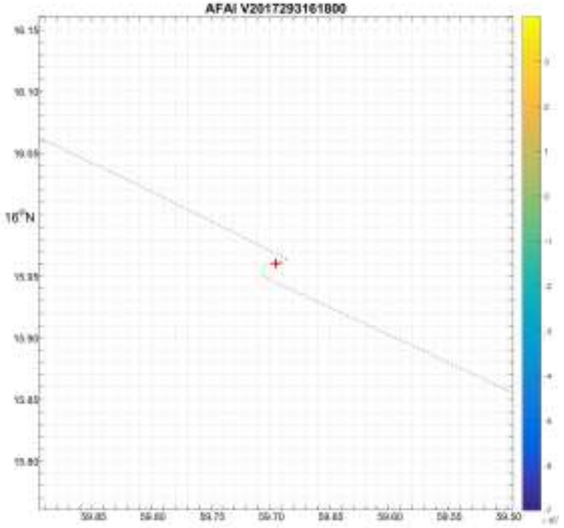

OLCI (300m) **MCI**

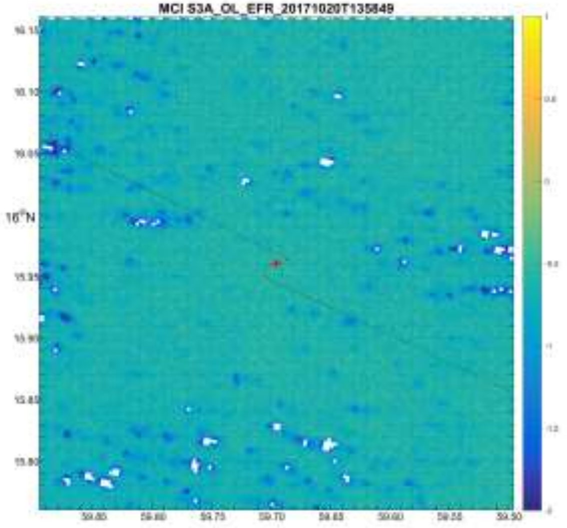

In situ - Type 2

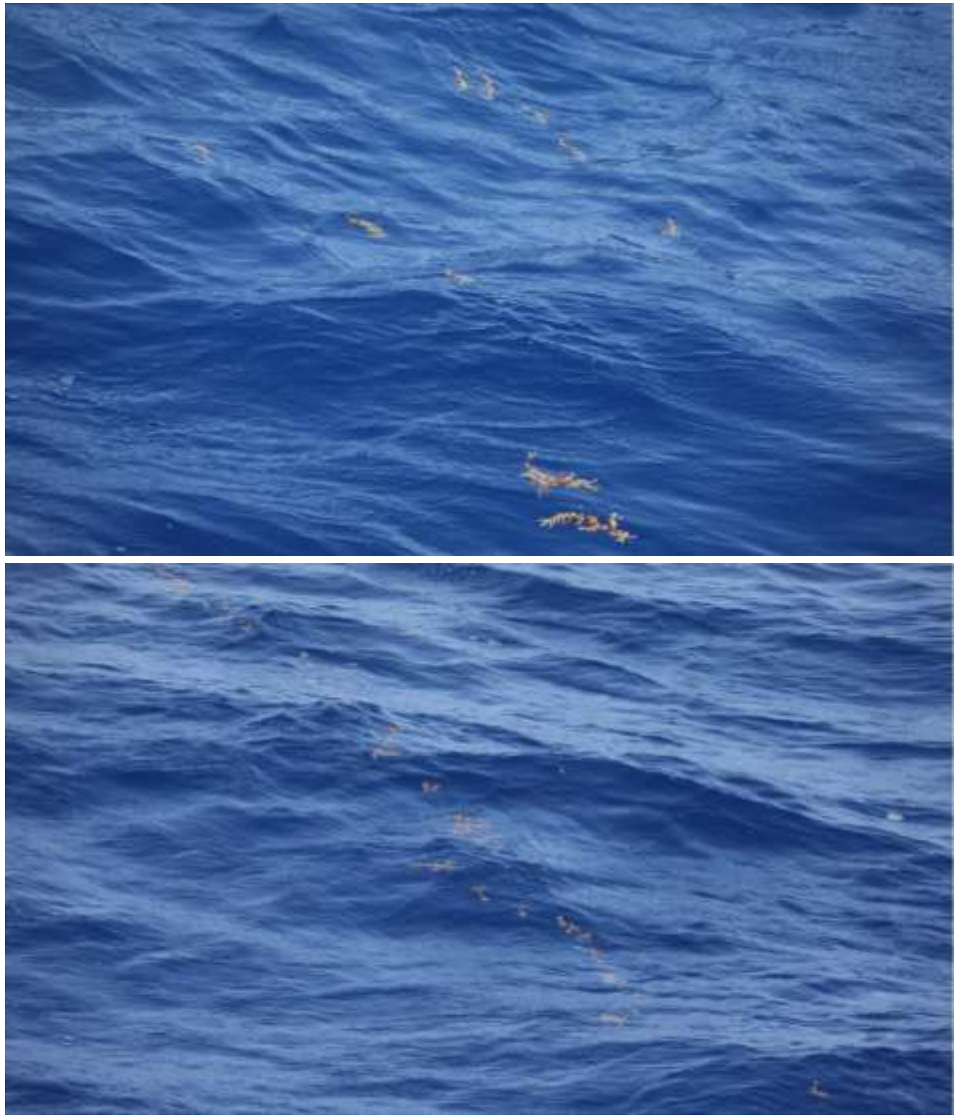

S2 Fig – 42. TRANSATLANTIC - Y15 - 2017-10-21 14.05 TU - 14°33.295'N 61°35.655'W - WS = 9.4 m.s^{-1} WD = 262° SS = Slight

MODIS-A (1km)

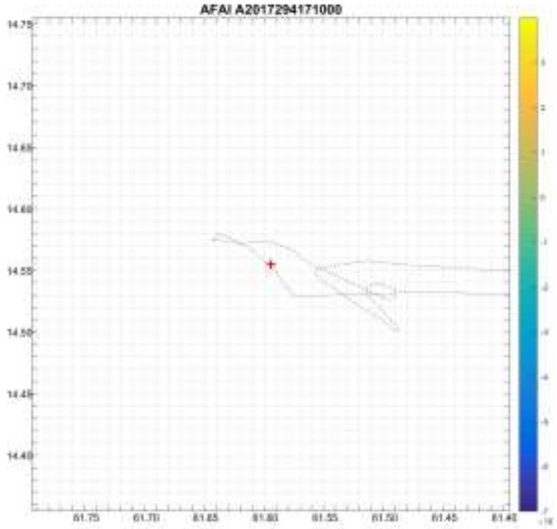

MODIS-T (1km)

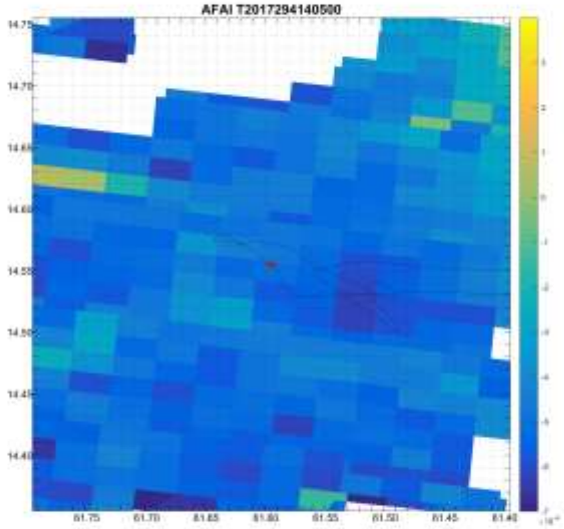

VIIRS (750 m)

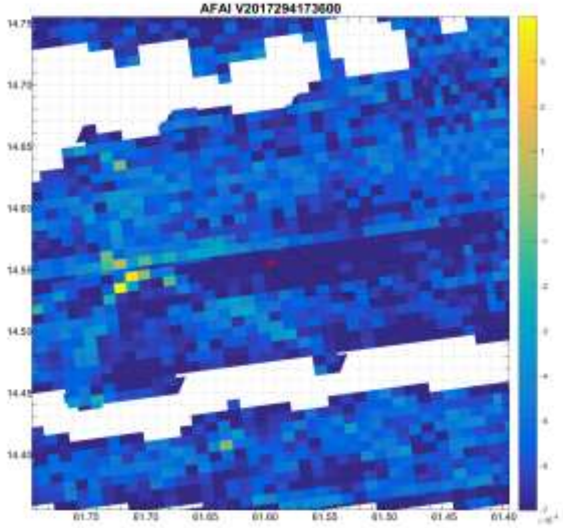

OLCI (300m) - 1 day

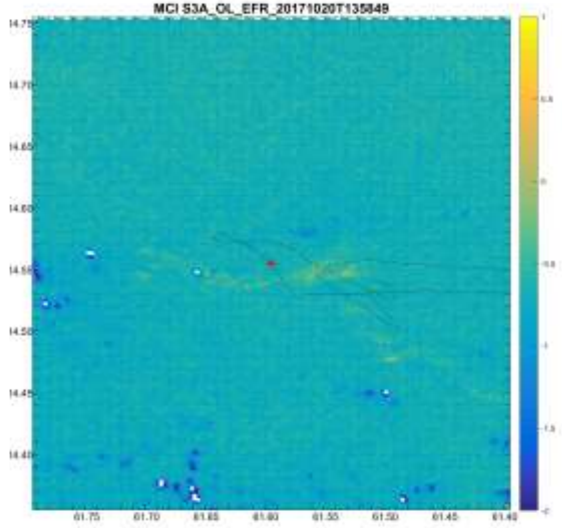

In situ - Type 3

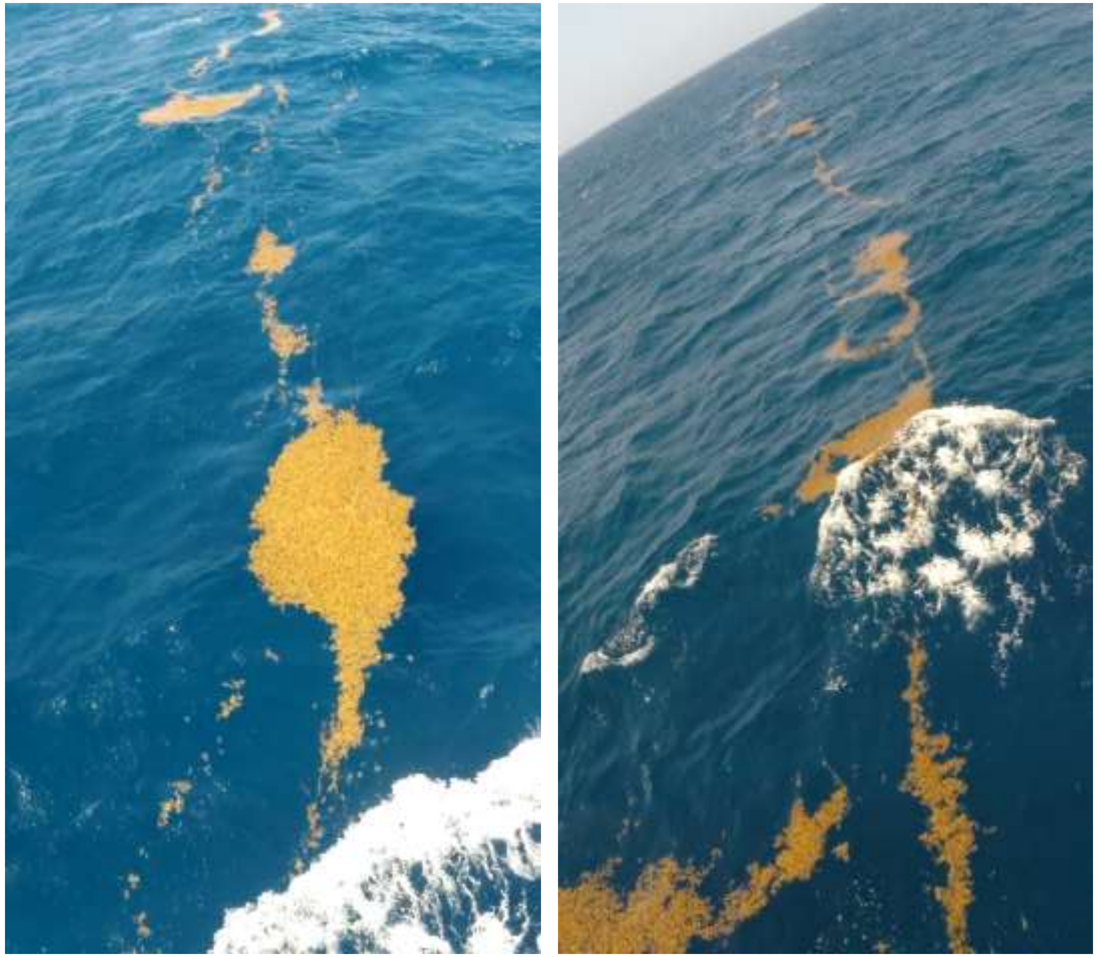

Supplement: S2 Fig — For each station, the following information is reported (see Table 2): cruise name, date, time (UTC), Latitude (°N), Longitude (°W), Wind Speed (WS) and Wind Direction (WD) and Sea State (SS). Satellite sensors maps are computed with images acquired on the day of the station for MODIS and VIIRS and for the day +/- 1 day for the OLCI sensor and in an area of 0.2° x 0.2° around the station. The date, time and name of sensor observations are reported as well as the algae index used to map Sargassum. (PDF) [file pone.0222584.s002.pdf]
